# Design, Synthesis, and Computational Insights into PKMYT1 Inhibitors for the Treatment of Breast Cancer

**DOI:** 10.3390/biomedicines13092116

**Published:** 2025-08-29

**Authors:** Jinyu Yu, Haoyu Zhang, Chuanxu Su, Shizhe Yuan, Nian Liu, Yin Sun, Yixiang Sun, Zixuan Gao, Dongmei Zhao, Maosheng Cheng

**Affiliations:** Key Laboratory of Structure-Based Drug Design and Discovery, Ministry of Education, School of Pharmaceutical Engineering, Shenyang Pharmaceutical University, 103 Wenhua Road, Shenhe District, Shenyang 110016, China

**Keywords:** CADD, PKMYT1 inhibitors, synthetic lethal

## Abstract

**Background**: Membrane-associated tyrosine-threonine protein kinase 1 (PKMYT1), which is identified as a synthetic lethal partner of *CCNE1*, emerged as a promising therapeutic target in oncology. **Methods**: A series of novel PKMYT1 inhibitors were designed by employing a pharmacophore fusion strategy. The underlying mechanisms were investigated by means of pharmacological experiments and molecular simulations. **Results**: Compound MY-14 demonstrated optimal kinase inhibition (IC_50_ = 0.002 μM) and significant anti-proliferative efficacy against *CCNE1*-amplified cells (IC_50-HCC1569_ = 1.06 μM and IC_50-OVCAR3_ = 0.80 μM). Furthermore, MY-14 induced concentration-dependent apoptosis, inhibited colony formation, and effectively arrested cell-cycle progression at the S-phase through synthetic lethality. Molecular dynamics simulations, Hirshfeld surface analysis, dynamic cross-correlation matrix (DCCM), and MM/GBSA calculations elucidated the molecular mechanism underlying MY-14’s interaction with PKMYT1. **Conclusions**: MY-14 emerged as a promising compound for the development of a novel PKMYT1 inhibitor.

## 1. Introduction

Dysregulation of cell-cycle control represents a core hallmark of tumorigenesis, with aberrant G1/S and G2/M checkpoints being critically involved in cellular homeostasis [1]. As a key member of the WEE kinase family [2,3], PKMYT1 (membrane-associated tyrosine/threonine-protein kinase 1) phosphorylates Thr14 and Tyr15 [4,5] residues on cyclin-dependent kinase 1 (CDK1), thereby suppressing the CDK1–cyclin B complex activity and exerting essential control over the G2/M transition [6,7]. Studies have demonstrated synthetic lethality between *CCNE1* amplification and PKMYT1 kinase inhibition [8]. Notably, tumors overexpressing *CCNE1* exhibit this synthetic lethality through dual impairment of these regulatory pathways, providing a mechanistic basis for PKMYT1-targeted anticancer strategies. In contrast to WEE1, which primarily localizes within the nucleus and phosphorylates solely Tyr15 [9,10], PKMYT1 displays predominant localization to the endoplasmic reticulum and Golgi apparatus, indicating broader subcellular distribution [11]. This unique subcellular compartmentalization establishes PKMYT1 as a promising therapeutic target [12,13].

**Figure 1:**
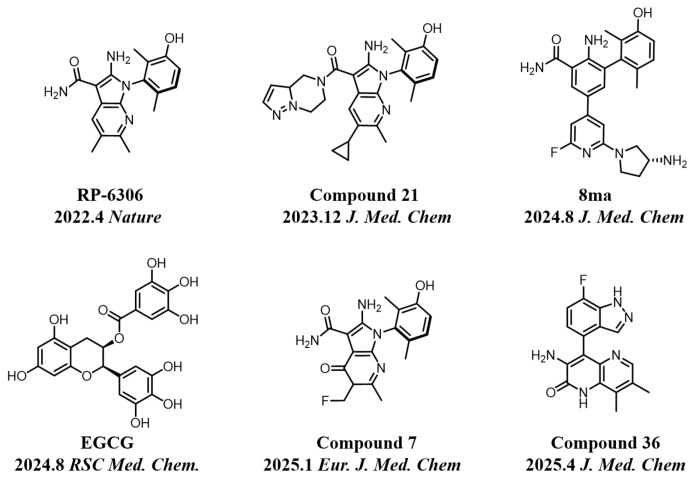
Some representative chemical structures of recent PKMYT1 inhibitors.

In recent years, kinase inhibitors have become crucial for cancer treatment, particularly with significant advances made in the development of PKMYT1 inhibitors [14,15]. As shown in Figure 1, the first-in-class inhibitor RP-6306 (Repare Therapeutics) entered Phase II clinical trials for *CCNE1*-amplified breast cancer [16]. Structure-based drug design (SBDD) and virtual screening have facilitated the discovery of novel inhibitors, including compounds 8ma [17], 21 [18], 7 [6], 36 [4], and the natural product EGCG [19], all demonstrating potent antitumor efficacy. However, current inhibitors face limitations: most derivatives are structurally analogous to RP-6306 or represent natural products resistant to synthetic optimization, restricting target exploitation potential. Consequently, developing novel chemotypes remains a critical unmet need in PKMYT1-targeted therapy.

In this study, we developed compound MY-1 through pharmacophore fusion, using M1 as the lead compound. MY-1 exhibited potent kinase inhibitory activity (IC_50_ = 0.026 μM). Subsequent optimization guided by structure-based drug design (SBDD) yielded the potent inhibitor MY-14. The mechanism of action was validated through cell cycle and apoptosis assays. Finally, multiple molecular simulations elucidated the binding mode of MY-14 with PKMYT1, revealing key interactions essential for its inhibitory efficacy.

## 2. Experimental Section

### 2.1. Chemistry

All reagents and solvents utilized in the experiments were acquired from commercial suppliers and did not undergo any additional purification. Thin-layer chromatography (TLC) plates were employed to monitor the reactions, which were examined under ultraviolet light at 254 or 365 nm. NMR spectra were recorded on Bruker Avance spectrometers operating at either 600 or 400 MHz, utilizing TMS as the internal standard, with CDCl_3_ (7.26 ppm) and DMSO-*d*_6_ (2.50 ppm) serving as reference standards; these spectra were obtained in solutions of CDCl_3_ or DMSO-*d*_6_. The Agilent 1100 LC-MS spectrometer was used to obtain low-resolution ESI-MS spectra, whereas high-resolution mass spectrometry (HRMS) data were gathered with the Agilent Q-TOF 6530 or 6550 mass spectrometer. The melting point was determined using the BÜCHI B-540 apparatus in an open capillary setup. All compounds were confirmed to possess a purity exceeding 95%. The purity was determined by HPLC using a Hua Spectrum S6000PLUS ultra-high-performance liquid chromatography system, equipped with an Agilent ZORBAX Eclipse XDB-C18 column. The sample injection conditions were specified as a methanol-to-water ratio of 8:2, with a column temperature of 25 °C. Spectral data for all compounds were provided in the Supplementary Materials.

1-bromo-2,4-dimethyl-3-nitrobenzene (T-1): A solution of 2,6-dimethylnitrobenzene (4.00 g, 26.48 mmol), bromine (6.35 g, 39.72 mmol), FeBr_3_ (0.16 g, 0.53 mmol), and iron powder (0.44 g, 7.94 mmol) were dissolved in chloroform (50 mL). After stirring at 0 °C for 12 h, the mixture was poured into a saturated sodium thiosulfate solution and extracted with methylene chloride. The organic phases were combined, washed with saturated brine, dried with anhydrous Na_2_SO_4_, and concentrated under reduced pressure. The residue was purified by petroleum ether pulping to obtain the white solid compound T-1 (3.25 g, 53.6% yield). ^1^H NMR (600 MHz, Chloroform-*d*) δ 7.55 (d, *J* = 8.2 Hz, 1H), 7.01 (d, *J* = 8.2 Hz, 1H), 2.34 (s, 3H), 2.25 (s, 3H).

1-methoxy-2,4-dimethyl-3-nitrobenzene (T-2): A solution of T-1 (3.25 g, 14.19 mmol), sodium methoxide (7.89 mL, 42.57 mmol), and copper(I) bromide (0.81 g, 5.68 mmol) in anhydrous *N,N*-dimethylformamide (DMF, 10 mL) was stirred at 90 °C under an argon atmosphere for 16 h. After cooling to room temperature, the reaction mixture was carefully poured into ice water (100 mL), resulting in the formation of a black-brown precipitate. The solid was collected by vacuum filtration and washed with cold water (3 × 20 mL). The crude product was purified by column chromatography on silica gel to afford T-2 as a light greenish-yellow crystalline solid (2.40 g, 93.4% yield). ^1^H NMR (600 MHz, Chloroform-*d*) δ 7.06 (d, *J* = 8.4, 1.2 Hz, 1H), 6.84 (d, *J* = 8.4 Hz, 1H), 3.84 (s, 3H), 2.22 (d, *J* = 0.8 Hz, 3H), 2.13 (s, 3H).

2,4-dimethyl-3-nitrophenol (T-3): A solution of T-2 (2.40 g, 10.48 mmol) in anhydrous dichloromethane (DCM, 8 mL) was cooled to −40 °C. To this stirred solution, a 2 M solution of boron trichloride (13.25 mL, 26.50 mmol) in DCM was added dropwise over 10 min. After complete addition, the reaction mixture was maintained at −40 °C for 15 min, then gradually warmed to room temperature and stirred for an additional 1.5 h. The reaction was carefully quenched by the addition of ice-cold methanol, and the solvent was evaporated under reduced pressure. The resulting residue was extracted with ethyl acetate (3 × 15 mL), and the combined organic layers were dried over anhydrous sodium sulfate (Na_2_SO_4_), filtered, and concentrated in vacuo to afford T-3 as a red oil (1.95 g, 88.1% yield). ^1^H NMR (600 MHz, Chloroform-*d*) δ 6.98 (d, *J* = 8.2 Hz, 1H), 6.79 (d, *J* = 8.3 Hz, 1H), 5.01 (s, 1H), 2.22 (s, 3H), 2.16 (s, 3H).

*tert*-butyl(2,4-dimethyl-3-nitrophenoxy)dimethylsilane (T-4): A solution of T-3 (1.95 g, 11.67 mmol), anhydrous potassium carbonate (K_2_CO_3_, 3.23 g, 23.34 mmol), and *tert*-butyldimethylsilyl chloride (TBSCl, 3.52 g, 23.34 mmol) in anhydrous dichloromethane (DCM, 8 mL) was stirred at room temperature under an inert atmosphere for 6 h. After completion, the reaction mixture was diluted with DCM (20 mL) and washed with water (20 mL) followed by brine (20 mL). The organic layer was separated, dried over anhydrous sodium sulfate (Na_2_SO_4_), filtered, and concentrated under reduced pressure to afford T-4 as a yellow oil (2.45 g, 74.7% yield). ^1^H NMR (600 MHz, Chloroform-*d*) δ 6.73 (d, 1H), 6.56 (d, *J* = 8.3 Hz, 1H), 1.99 (s, 3H), 1.91 (s, 3H), 0.79 (s, 9H), 0.00 (s, 6H).

3-((*tert*-butyldimethylsilyl)oxy)-2,6-dimethylaniline (T-5): A solution of T-4 (2.45 g, 8.71 mmol) in ethanol (8 mL) and saturated aqueous ammonium chloride (2 mL) was treated with iron powder (2.44 g, 43.55 mmol). The reaction mixture was stirred vigorously at 80 °C for 4 h. After cooling to room temperature, the mixture was filtered, and the filter cake was washed thoroughly with ethanol (3 × 15 mL). The combined filtrates were concentrated under reduced pressure, and the resulting residue was extracted with ethyl acetate (3 × 50 mL). The combined organic layers were washed with brine (50 mL), dried over anhydrous sodium sulfate (Na_2_SO_4_), filtered, and concentrated in vacuo. Purification by flash column chromatography afforded T-5 as a pale yellow oil (1.95 g, 89.1% yield). ^1^H NMR (600 MHz, Chloroform-*d*) δ 6.57 (d, 1H), 6.04 (d, *J* = 8.1 Hz, 1H), 1.92 (d, *J* = 0.7 Hz, 3H), 1.86 (s, 3H), 0.82 (s, 9H), 0.00 (s, 6H).

2,4-dichloro-*N*-(3-((tert-butyldimethylsilyl)oxy)-2,6-dimethylphenyl)pyrimidine-5-carboxamide (T-6): A mixture of T-5 (1.61 g, 6.40 mmol) and 2,4-dichloro-5-pyrimidinecarbonyl chloride (1 g, 6.40 mmol) in anhydrous THF (10 mL) was stirred at room temperature for 4 h. Upon completion, the resulting precipitate was collected by vacuum filtration and further purified by column chromatography to afford T-6 as a white solid (1.50 g, 55.1% yield). ^1^H NMR (600 MHz, Chloroform-*d*) δ 8.80 (s, 1H), 7.51 (s, 1H), 6.76 (d, *J* = 8.3 Hz, 1H), 6.52 (d, *J* = 8.2 Hz, 1H), 2.00 (s, 3H), 1.93 (s, 3H), 0.79 (s, 9H), 0.00 (s, 6H).

4-amino-2-chloro-*N*-(3-((tert-butyldimethylsilyl)oxy)-2,6-dimethylphenyl)pyrimidine-5-carboxamide (T-7): A solution of T-6 (1.5 g, 3.53 mmol) in anhydrous 1,4-dioxane (10 mL) was treated with ammonia solution in dioxane (5 mL). The reaction mixture was stirred for 2 h at ambient temperature. Upon completion, the mixture was concentrated under reduced pressure to remove excess ammonia, then diluted with water (20 mL) and extracted with ethyl acetate (3 × 30 mL). The combined organic layers were washed with brine (30 mL), dried over anhydrous sodium sulfate (Na_2_SO_4_), filtered, and concentrated in vacuo. The crude product was purified by trituration with petroleum ether/ethyl acetate (3:1, *v*/*v*) to afford T-7 as a white crystalline solid (1.05 g, 73.2% yield). ^1^H NMR (600 MHz, Chloroform-*d*) δ 8.38 (s, 1H), 7.26 (s, 1H), 6.76 (d, *J* = 8.3 Hz, 1H), 6.52 (d, *J* = 8.2 Hz, 1H), 1.97 (s, 3H), 1.90 (s, 3H), 0.80 (s, 9H), −0.00 (s, 6H).

4-amino-2-(phenylamino)-*N*-(3-hydroxy-2,6-dimethylphenyl)pyrimidine-5-carboxamide (MY-1): White solid; yield: 46.7%; mp: 273.5–274.7 °C; ^1^H NMR (600 MHz, DMSO-*d*_6_) δ 9.45 (d, *J* = 15.0 Hz, 2H), 9.16 (s, 1H), 8.78 (s, 1H), 7.83 (d, *J* = 8.0 Hz, 2H), 7.29 –7.24 (m, 2H), 6.95 (t, *J* = 7.3 Hz, 1H), 6.88 (d, *J* = 8.2 Hz, 1H), 6.68 (d, *J* = 8.3 Hz, 1H), 2.06 (s, 3H), 1.99 (s, 3H). ^13^C NMR (151 MHz, DMSO) δ 165.8, 163.6, 160.6, 157.7, 154.1, 140.9, 136.2, 128.9, 127.3, 126.1, 122.9, 122.0, 119.7, 113.5, 100.5, 18.1, 11.6. HRMS (ESI) for C_19_H_19_N_5_O_6_ [M+Na]^+^: calcd, 372.1491, found, 372.1422. HPLC: 99.421%.

4-amino-2-((2-chlorophenyl)amino)-*N*-(3-hydroxy-2,6-dimethylphenyl)pyrimidine-5-carboxamide (MY-2): White solid; yield: 39.1% mp: 256.9–257.9 °C; ^1^H NMR (600 MHz, DMSO-*d*_6_) δ 9.46 (s, 1H), 9.16 (s, 1H), 8.73 (s, 1H), 8.51 (s, 1H), 7.94 (d, *J* = 8.3 Hz, 1H), 7.49 (d, *J* = 8.0 Hz, 1H), 7.33 (t, *J* = 7.8 Hz, 1H), 7.15 (t, *J* = 7.7 Hz, 1H), 6.88 (d, *J* = 8.2 Hz, 1H), 6.68 (d, *J* = 8.2 Hz, 1H), 2.05 (s, 3H), 1.98 (s, 3H). ^13^C NMR (151 MHz, DMSO) δ 165.7, 163.7, 161.0, 157.8, 154.1, 136.8, 136.1, 129.8, 127.9, 127.4, 127.3, 126.4, 126.1, 125.7, 122.9, 113.6, 101.1, 18.0, 11.6. HRMS (ESI) for C_19_H_18_ClN_5_O_2_ [M+Na]^+^: calcd, 406.1047, found, 406.1022. HPLC: 98.066%.

4-amino-2-((3-chlorophenyl)amino)-*N*-(3-hydroxy-2,6-dimethylphenyl)pyrimidine-5-carboxamide (MY-3): White solid; yield: 42.3%; mp: 261.3–262.9 °C; ^1^H NMR (600 MHz, DMSO-*d*_6_) δ 9.67 (s, 1H), 9.48 (s, 1H), 9.17 (s, 1H), 8.80 (s, 1H), 8.02 (t, *J* = 2.1 Hz, 1H), 7.77 (d, *J* = 8.3, 2.1 Hz, 1H), 7.28 (t, *J* = 8.1 Hz, 1H), 6.98 (d, *J* = 8.0, 2.1 Hz, 1H), 6.88 (d, *J* = 8.2 Hz, 1H), 6.69 (d, *J* = 8.2 Hz, 1H), 2.07 (s, 3H), 1.99 (s, 3H). ^13^C NMR (151 MHz, DMSO) δ 165.6, 163.5, 160.4, 157.7, 154.1, 142.5, 136.1, 133.4, 130.5, 127.3, 126.1, 122.8, 121.4, 118.7, 117.9, 113.6, 101.0, 18.0, 11.6. HRMS (ESI) for C_19_H_18_ClN_5_O_2_ [M+Na]^+^: calcd, 406.1047, found, 406.1031. HPLC: 96.054%.

4-amino-2-((4-chlorophenyl)amino)-*N*-(3-hydroxy-2,6-dimethylphenyl) (MY-4): White solid; yield: 37.6%; mp: 238.5–240.9 °C; ^1^H NMR (600 MHz, DMSO-*d*_6_) δ 9.63 (s, 1H), 9.46 (s, 1H), 8.78 (s, 1H), 7.88 (d, *J* = 9.0 Hz, 2H), 7.30 (d, *J* = 8.8 Hz, 2H), 6.88 (d, *J* = 8.2 Hz, 1H), 6.68 (d, *J* = 8.3 Hz, 1H), 2.06 (s, 3H), 1.98 (s, 3H). ^13^C NMR (151 MHz, DMSO) δ 164.6, 162.4, 158.8, 156.6, 153.1, 138.9, 135.1, 127.1, 126.2, 125.0, 124.3, 121.8, 120.6, 111.9, 99.7, 54.9, 17.9, 15.6, 9.7. HRMS (ESI) for C_19_H_18_ClN_5_O_2_ [M+Na]^+^: calcd, 406.1047, found, 406.1041. HPLC: 97.481%.

4-amino-2-((4-fluorophenyl)amino)-*N*-(3-hydroxy-2,6-dimethylphenyl)pyrimidine-5-carboxamide (MY-5): White solid; yield: 48.9%; mp: 282.5–320.2 °C; ^1^H NMR (600 MHz, DMSO-*d*_6_) δ 9.51 (s, 1H), 9.43 (s, 1H), 9.15 (s, 1H), 7.84 (d, *J* = 9.0, 5.1 Hz, 2H), 7.10 (d, *J* = 8.9 Hz, 2H), 6.88 (d, *J* = 8.2 Hz, 1H), 6.68 (d, *J* = 8.2 Hz, 1H), 2.06 (s, 3H), 1.98 (s, 3H). ^13^C NMR (151 MHz, DMSO) δ 166.7, 163.5, 160.0, 159.2, 157.7, 156.2, 154.1, 137.3, 135.5, 127.3, 125.5, 123.4, 121.3, 115.4, 115.2, 113.5, 100.5, 17.6, 11.6. HRMS (ESI) for C_19_H_18_FN_5_O_2_ [M+Na]^+^: calcd, 390.1343, found, 390.1339. HPLC: 99.218%.

4-amino-2-((4-bromophenyl)amino)-*N*-(3-hydroxy-2,6-dimethylphenyl)pyrimidine-5-carboxamide (MY-6): Yellow solid; yield: 56.3%; mp: 242.9–245.5 °C; ^1^H NMR (600 MHz, DMSO-*d*_6_) δ 9.63 (s, 1H), 9.46 (s, 1H), 8.78 (s, 1H), 7.83 (d, *J* = 9.0 Hz, 2H), 7.42 (d, *J* = 9.0 Hz, 2H), 6.87 (d, *J* = 8.2 Hz, 1H), 6.68 (d, *J* = 8.1 Hz, 1H), 2.06 (s, 3H), 1.98 (s, 3H). ^13^C NMR (151 MHz, DMSO) δ 165.6, 163.5, 160.4, 157.7, 154.2, 140.4, 136.2, 131.6, 127.3, 126.0, 122.9, 121.5, 113.6, 113.3, 100.1, 56.5, 19.0, 18.0, 11.6. HRMS (ESI) for C_19_H_18_BrN_5_O_2_ [M+Na]^+^: calcd, 450.0542, found, 450.0574. HPLC: 98.250%.

4-amino-2-(*p*-tolylamino)-*N*-(3-hydroxy-2,6-dimethylphenyl)pyrimidine-5-carboxamide (MY-7): Light white solid; yield: 52.1% mp: 264.0–264.5 °C; ^1^H NMR (600 MHz, DMSO-*d*_6_) δ 9.41 (s, 1H), 9.36 (s, 1H), 9.15 (s, 1H), 8.76 (s, 1H), 7.70 (d, *J* = 8.5 Hz, 2H), 7.07 (d, *J* = 8.2 Hz, 2H), 6.88 (d, *J* = 8.2 Hz, 1H), 6.68 (d, *J* = 8.1 Hz, 1H), 2.25 (s, 3H), 2.06 (s, 3H), 1.98 (s, 3H). ^13^C NMR (151 MHz, DMSO) δ 165.8, 163.6, 160.7, 158.4, 154.7, 139.2, 136.8, 131.8, 129.3, 127.3, 126.1, 122.9, 119.9, 114.4, 100.2, 20.9, 17.4, 10.3. HRMS (ESI) for C_20_H_21_N_5_O_2_ [M+Na]^+^: calcd, 386.1593, found, 386.1586. HPLC: 95.956%.

4-amino-2-((4-isopropylphenyl)amino)-*N*-(3-hydroxy-2,6-dimethylphenyl)pyrimidine-5-carboxamide (MY-8): White solid; yield: 46.8% mp: 248.2–250.2 °C; ^1^H NMR (600 MHz, DMSO-*d*_6_) δ 9.41 (s, 1H), 9.36 (s, 1H), 9.15 (s, 1H), 8.76 (s, 1H), 7.70 (d, *J* = 8.2 Hz, 2H), 7.13 (d, *J* = 8.6 Hz, 2H), 6.88 (d, *J* = 8.2 Hz, 1H), 6.68 (d, *J* = 8.1 Hz, 1H), 2.83 (hept, *J* = 6.9 Hz, 1H), 2.06 (s, 3H), 1.98 (s, 3H), 1.19 (d, *J* = 6.9 Hz, 6H). ^13^C NMR (151 MHz, DMSO) δ 165.8, 163.6, 161.8, 158.7, 154.1, 143.1, 138.6, 136.3, 127.3, 126.6, 126.1, 122.9, 120.1, 113.5, 100.3, 33.3, 24.5, 18.1, 11.6, 0.6. HRMS (ESI) for C_22_H_25_N_5_O_2_ [M+Na]^+^: calcd, 414.1906, found, 414.1925. HPLC: 97.348%.

4-amino-2-([1,1′-biphenyl]-4-ylamino)-*N*-(3-hydroxy-2,6-dimethylphenyl)pyrimidine-5-carboxamide (MY-9): White solid; yield: 39.7% mp: 249.0–249.6 °C; ^1^H NMR (600 MHz, DMSO-*d*_6_) δ 9.61 (s, 1H), 9.46 (s, 1H), 9.18 (s, 1H), 8.80 (s, 1H), 7.94 (d, *J* = 8.2 Hz, 2H), 7.65 (d, *J* = 7.7 Hz, 2H), 7.59 (d, *J* = 8.3 Hz, 2H), 7.44 (t, *J* = 7.6 Hz, 2H), 7.31 (t, *J* = 7.4 Hz, 1H), 6.88 (d, *J* = 8.2 Hz, 1H), 6.69 (d, *J* = 8.2 Hz, 1H), 2.07 (s, 3H), 1.99 (s, 3H). ^13^C NMR (151 MHz, DMSO) δ 165.7, 164.0, 160.6, 157.7, 154.1, 141.0, 136.2, 133.6, 130.1, 129.4, 127.3, 127.2, 127.1, 126.6, 126.1, 122.9, 120.0, 113.6, 100.6, 82.7, 18.1, 11.6. HRMS (ESI) for C_25_H_23_N_5_O_2_ [M+Na]^+^: calcd, 448.1750, found, 448.1758. HPLC: 96.096%.

4-amino-2-((4-morpholinophenyl)amino)-*N*-(3-hydroxy-2,6-dimethylphenyl)pyrimidine-5-carboxamide (MY-10): Yellow solid; yield: 63.6% mp: 270.0–280.0 °C; ^1^H NMR (600 MHz, DMSO-*d*_6_) δ 9.38 (s, 1H), 9.23 (s, 1H), 9.15 (s, 1H), 8.73 (s, 1H), 7.64 (d, *J* = 8.5 Hz, 2H), 6.88 (d, 1H), 6.86 (s, 2H), 6.68 (d, *J* = 8.2 Hz, 1H), 3.76–3.71 (m, 4H), 3.05–3.01 (m, 4H), 2.06 (s, 3H), 1.98 (s, 3H). ^13^C NMR (151 MHz, DMSO) δ 166.4, 164.2, 160.7, 158.7, 154.6, 146.8, 136.3, 132.7, 128.4, 126.7, 123.5, 121.6, 117.3, 114.6, 99.9, 66.2, 49.7, 18.7, 11.2. HRMS (ESI) for C_23_H_26_N_6_O_3_ [M+Na]^+^: calcd, 457.1964, found, 457.1971. HPLC: 97.409%.

4-amino-2-((4-(4-methylpiperazin-1-yl)phenyl)amino)-*N*-(3-hydroxy-2,6-dimethylphenyl)pyrimidine-5-carboxamide (MY-11): White solid; yield: 48.8% mp: 276.9–278.7 °C; ^1^H NMR (600 MHz, DMSO-*d*_6_) δ 9.38 (s, 1H), 9.21 (s, 1H), 9.15 (s, 1H), 8.73 (s, 1H), 7.61 (d, *J* = 8.5 Hz, 2H), 6.87 (d, *J* = 8.2 Hz, 2H), 6.85 (s, 1H), 6.68 (d, *J* = 8.2 Hz, 1H), 3.08–3.03 (m, 4H), 2.47–2.43 (m, 4H), 2.22 (s, 3H), 2.06 (s, 3H), 1.98 (s, 3H). ^13^C NMR (151 MHz, DMSO) δ 207.4, 165.9, 163.6, 160.7, 157.8, 154.1, 146.8, 136.3, 132.9, 127.3, 125.6, 122.9, 121.2, 116.2, 113.5, 100.5, 54.1, 48.7, 46.7, 18.1, 11.6. HRMS (ESI) for C_24_H_29_N_7_O_2_ [M+Na]^+^: calcd, 470.2281, found, 470.2271. HPLC: 97.778%.

4-amino-2-((4-nitrophenyl)amino)-*N*-(3-hydroxy-2,6-dimethylphenyl)pyrimidine-5-carboxamide (MY-12): Light yellow solid; yield: 32.2% mp: 278.5–279.3 °C; ^1^H NMR (600 MHz, DMSO-*d*_6_)δ 10.28 (s, 1H), 9.59 (s, 1H), 9.26 (s, 1H), 8.86 (s, 1H), 8.17 (d, *J* = 8.2 Hz, 2H), 8.13 (d, *J* = 9.9 Hz, 2), 6.88 (d, *J* = 8.2 Hz, 1H), 6.70 (d, *J* = 8.2 Hz, 1H), 2.07 (s, 3H), 1.99 (s, 3H). ^13^C NMR (151 MHz, DMSO) δ 165.4, 163.5, 160.0, 157.1, 154.2, 147.7, 141.5, 136.0, 127.3, 126.0, 125.3, 122.8, 118.6, 113.7, 102.0, 18.0, 11.6. HRMS (ESI) for C_19_H_18_N_6_O_4_ [M+Na]^+^: calcd, 417.1288, found, 417.1275. HPLC: 96.217%.

4-amino-2-((4-(dimethylphosphoryl)phenyl)amino)-*N*-(3-hydroxy-2,6-dimethylphenyl)pyrimidine-5-carboxamide (MY-13): White solid; yield: 54.3% mp: 238.5–239.7 °C; ^1^H NMR (600 MHz, DMSO-*d*_6_) δ 9.75 (s, 1H), 9.50 (s, 1H), 9.20 (s, 1H), 8.81 (s, 1H), 7.98 (dd, 2H), 7.63 (dd, *J* = 11.0, 8.5 Hz, 2H), 6.88 (d, *J* = 8.2 Hz, 1H), 6.69 (d, *J* = 8.2 Hz, 1H), 2.07 (s, 3H), 1.99 (s, 3H), 1.63 (s, 3H), 1.60 (s, 3H). ^13^C NMR (151 MHz, DMSO) δ 165.2, 162.0, 160.1, 157.9, 153.1, 142.6, 135.7, 129.6, 129.1, 127.3, 126.7, 126.2, 124.5, 121.8, 117.9, 112.5, 100.0, 17.6, 17.1, 17.0, 10.5. HRMS (ESI) for C_21_H_24_N_5_O_3_P [M+Na]^+^: calcd, 448.1515, found, 448.1546. HPLC: 98.481%.

(4-((4-amino-5-((3-hydroxy-2,6-dimethylphenyl)carbamoyl)pyrimidin-2-yl)amino)phenyl)phosphonate (MY-14): White solid; yield: 52.1% mp: 232.1–232.7 °C; ^1^H NMR (400 MHz, DMSO-*d*_6_) δ 9.85 (s, 1H), 9.51 (s, 1H), 9.16 (s, 1H), 8.81 (s, 1H), 8.02 (dd, *J* = 8.7, 3.6 Hz, 2H), 7.59 (dd, *J* = 12.6, 8.4 Hz, 2H), 6.88 (d, *J* = 8.1 Hz, 1H), 6.69 (d, *J* = 8.1 Hz, 1H), 3.98 (dtq, *J* = 10.2, 6.5, 3.2 Hz, 4H), 2.07 (s, 3H), 1.99 (s, 3H), 1.23 (t, *J* = 7.0 Hz, 6H). ^13^C NMR (101 MHz, DMSO) δ 165.5, 163.5, 160.4, 156.9, 154.1, 144.8, 136.1, 133.5, 132.5, 132.4, 127.3, 126.1, 123.4, 121.0, 119.1, 118.9, 118.8, 113.6, 101.3, 62.7, 19.5, 16.2, 10.4. HRMS (ESI) for C_23_H_28_N_5_O_5_P [M+Na]^+^: calcd, 508.1726, found, 508.1732. HPLC: 99.295%.

4-amino-2-((4-(dimethylcarbamoyl)phenyl)amino)-*N*-(3-hydroxy-2,6-dimethylphenyl)pyrimidine-5-carboxamide (MY-15): White solid; yield: 41.5% mp: 236.3–237.5 °C; ^1^H NMR (600 MHz, DMSO-*d*_6_) δ 9.70 (s, 1H), 9.48 (s, 1H), 9.20 (s, 1H), 8.80 (s, 1H), 7.90 (d, *J* = 8.2 Hz, 2H), 7.34 (d, *J* = 8.3 Hz, 2H), 6.88 (d, *J* = 8.2 Hz, 1H), 6.69 (d, *J* = 8.2 Hz, 1H), 2.97 (s, 6H), 2.07 (s, 3H), 1.99 (s, 3H). ^13^C NMR (151 MHz, DMSO) δ 172.4, 165.7, 163.5, 161.4, 158.6, 154.2, 142.7, 135.5, 129.3, 128.3, 127.3, 126.1, 122.9, 118.1, 113.6, 101.4, 29.0, 27.0, 18.0, 10.6. HRMS (ESI) for C_22_H_24_N_6_O_3_ [M+Na]^+^: calcd, 443.1808, found, 443.1813. HPLC: 98.055%.

4-amino-2-((4-(morpholine-4-carbonyl)phenyl)amino)-*N*-(3-hydroxy-2,6-dimethylphenyl)pyrimidine-5-carboxamide (MY-16): White solid; yield: 44.5% mp: 235.4–237.4 °C; ^1^H NMR (600 MHz, DMSO-*d*_6_) δ 9.72 (s, 1H), 9.48 (s, 1H), 9.15 (s, 1H), 8.80 (s, 1H), 7.92 (d, *J* = 8.3 Hz, 2H), 7.34 (d, *J* = 8.6 Hz, 2H), 6.88 (d, *J* = 8.2 Hz, 1H), 6.69 (d, *J* = 8.2 Hz, 1H), 3.60 (s, 4H), 3.51 (s, 4H), 2.07 (s, 3H), 1.99 (s, 3H). ^13^C NMR (151 MHz, DMSO) δ 170.6, 165.6, 163.0, 161.2, 157.3, 153.0, 143.6, 135.3, 130.1, 128.5, 128.3, 127.3, 126.1, 122.9, 118.8, 113.6, 100.9, 65.6, 54.7, 18.0, 11.1. HRMS (ESI) for C_24_H_26_N_6_O_4_ [M+Na]^+^: calcd, 485.1914, found, 485.1909. HPLC: 98.595%.

4-amino-2-((2-methyl-1-oxoisoindolin-5-yl)amino)-*N*-(3-hydroxy-2,6-dimethylphenyl)pyrimidine-5-carboxamide (MY-17): White solid; yield: 48.9% mp: 317.7–318.5 °C; ^1^H NMR (600 MHz, DMSO-*d*_6_) δ 9.85 (s, 1H), 9.49 (s, 1H), 9.17 (s, 1H), 8.82 (s, 1H), 8.23 (s, 1H), 7.83 (dd, *J* = 8.4, 1.9 Hz, 1H), 7.54 (d, *J* = 8.4 Hz, 1H), 6.89 (d, *J* = 8.2 Hz, 1H), 6.69 (d, *J* = 8.2 Hz, 1H), 4.42 (s, 2H), 3.05 (s, 3H), 2.07 (s, 3H), 1.99 (s, 3H). ^13^C NMR (151 MHz, DMSO) δ 166.8, 164.5, 163.2, 159.4, 156.6, 153.1, 144.9, 142.2, 135.1, 126.7, 125.0, 124.8, 122.2, 121.8, 117.9, 112.5, 112.0, 100.0, 50.8, 28.5, 17.0, 9.8. HRMS (ESI) for C_22_H_22_N_6_O_3_ [M+Na]^+^: calcd, 441.1651, found, 441.1648. HPLC: 98.880%.

4-amino-2-(benzofuran-6-ylamino)-*N*-(3-hydroxy-2,6-dimethylphenyl)pyrimidine-5-carboxamide (MY-18): White solid; yield: 53.2% mp: 223.2–224.3 °C; ^1^H NMR (600 MHz, DMSO-*d*_6_) δ 9.68 (s, 1H), 9.48 (s, 1H), 9.16 (s, 1H), 8.80 (s, 1H), 8.44 (s, 1H), 7.88 (d, *J* = 2.2 Hz, 1H), 7.53–7.46 (m, 2H), 6.88 (d, *J* = 8.3 Hz, 1H), 6.87 (d, *J* = 2.5 Hz, 1H), 6.69 (d, *J* = 8.2 Hz, 1H), 2.07 (s, 3H), 1.99 (s, 3H). ^13^C NMR (151 MHz, DMSO) δ 164.6, 162.5, 159.2, 154.2, 153.0, 143.0, 137.6, 134.3, 125.7, 124.1, 121.2, 120.6, 119.1, 115.1, 111.9, 105.9, 101.4, 100.0, 54.3, 17.0, 10.5. HRMS (ESI) for C_21_H_19_N_5_O_3_ [M+Na]^+^: calcd, 412.1386, found, 412.1393. HPLC: 95.509%.

4-amino-2-(benzo[*d*]thiazol-6-ylamino)-*N*-(3-hydroxy-2,6-dimethylphenyl)pyrimidine-5-carboxamide (MY-19): White solid; yield: 47.7% mp: 281.9–283.1 °C; ^1^H NMR (600 MHz, DMSO-*d*_6_) δ 9.82 (s, 1H), 9.48 (s, 1H), 9.20 (s, 1H), 9.16 (s, 1H), 8.99 (s, 1H), 8.82 (s, 1H), 7.96 (d, *J* = 8.8 Hz, 1H), 7.75 (dd, *J* = 8.8, 2.2 Hz, 1H), 6.89 (d, *J* = 8.2 Hz, 1H), 6.69 (d, *J* = 8.2 Hz, 1H), 2.08 (s, 3H), 2.00 (s, 3H). ^13^C NMR (151 MHz, DMSO) δ 166.7, 163.6, 160.0, 157.2, 154.1, 153.9, 148.4, 139.0, 136.2, 134.8, 127.3, 126.1, 123.0, 122.9, 119.5, 113.6, 111.2, 100.7, 18.1, 11.6. HRMS (ESI) for C_20_H_18_N_6_O_2_S [M+Na]^+^: calcd, 429.1110, found, 429.1111. HPLC: 98.832%.

4-amino-2-(benzo[*d*]thiazol-5-ylamino)-*N*-(3-hydroxy-2,6-dimethylphenyl)pyrimidine-5-carboxamide (MY-20): White solid; yield: 43.6% mp: 263.5–264.5 °C; ^1^H NMR (600 MHz, DMSO-*d*_6_) δ 9.73 (s, 1H), 9.48 (s, 1H), 9.34 (s, 1H), 9.16 (s, 1H), 8.83 (s, 1H), 8.77 (d, *J* = 2.1 Hz, 1H), 8.01 (d, *J* = 8.7 Hz, 1H), 7.86 (dd, *J* = 8.8, 2.2 Hz, 1H), 6.89 (d, *J* = 8.2 Hz, 1H), 6.69 (d, *J* = 8.3 Hz, 1H), 2.08 (s, 3H), 2.00 (s, 3H). ^13^C NMR (151 MHz, DMSO) δ 165.7, 163.6, 160.7, 157.7, 156.8, 154.8, 154.1, 139.7, 136.2, 127.3, 126.6, 126.1, 123.3, 122.2, 119.6, 114.2, 113.0, 100.7, 18.1, 11.6. HRMS (ESI) for C_20_H_18_N_6_O_2_S [M+Na]^+^: calcd, 429.1110, found, 429.1090. HPLC: 97.164%.

4-amino-2-(benzofuran-5-ylamino)-*N*-(3-hydroxy-2,6-dimethylphenyl)pyrimidine-5-carboxamide (MY-21): White solid; yield: 47.8% mp: 251.9–252.9 °C; ^1^H NMR (600 MHz, DMSO-*d*_6_) δ 9.48 (s, 1H), 9.42 (s, 1H), 9.16 (s, 1H), 8.78 (s, 1H), 8.21 (d, *J* = 2.3 Hz, 1H), 7.93 (d, *J* = 2.2 Hz, 1H), 7.61 (dd, *J* = 8.9, 2.2 Hz, 1H), 7.48 (d, *J* = 8.9 Hz, 1H), 6.89 (t, 1H), 6.88 (s, 1H), 6.69 (d, *J* = 8.2 Hz, 1H), 2.07 (s, 3H), 1.99 (s, 3H). ^13^C NMR (151 MHz, DMSO) δ 166.6, 163.6, 160.8, 158.2, 154.1, 150.6, 147.3, 136.8, 127.6, 127.3, 126.2, 122.9, 118.1, 113.5, 112.0, 111.2, 107.4, 98.6, 55.4, 18.1, 11.6. HRMS (ESI) for C_21_H_19_N_5_O_3_ [M+Na]^+^: calcd, 412.1386, found, 412.1384. HPLC: 96.522%.

### 2.2. PKMYT1 Enzyme Inhibition Assays

The PKMYT1 kinase assay was conducted with the LanthaScreen™ Eu kinase binding method, as provided by Thermo Fisher Scientific (Waltham, MA, USA). Initially, compounds were prepared at a concentration of 4 mM in DMSO and subsequently diluted to various concentrations in 1X buffer. A mixture of 2.5 nM PKMYT1 kinase and 2 nM Eu-Anti-GST antibody was dispensed into a 384-well white plate. Subsequently, 4 µL of the test compounds and 4 µL of Trancer178 (2.5 nM) were introduced into the plate, followed by centrifugation at 1000 rpm for 1 min. The plate was then incubated in the dark at 25 °C for 1 h, and fluorescence signals were recorded at 665 nm and 615 nm.

### 2.3. Cell Proliferation Assay

HCC1569 and OVCAR3 cells (2 × 10^3^/well) were seeded in 96-well plates and cultured for 24 h at 37 °C, 5% CO_2_. Compounds at varying concentrations were added, followed by 5-day incubation. Cell viability was assessed using Cell Counting Kit-8 (CCK-8) with absorbance measurement at 450 nm. HCC1569 (BNCC341698) was purchased from BNCC and OVCAR3 (CL-0178) was purchased from Procell.

### 2.4. Cell Cloning Assay

OVCAR3 cells (4 × 10^3^/well) were seeded into 6-well plates and incubated for 24 h at 37 °C in a 5% CO_2_ atmosphere. Compound MY-14 was then added at varying concentrations, followed by a 14-day incubation period. Subsequently, cells were fixed with a 4% paraformaldehyde solution and stained using crystal violet solution, after which colonies were quantified using ImageJ 1.54 software.

### 2.5. Cell Apoptosis Assay

HCC1569 cells (1 × 10^5^/well) were seeded in 6-well plates and cultured for 24 h at 37 °C, 5% CO_2_. MY-14 at varying concentrations were added, followed by 5-day incubation. Cells were trypsinized and pelleted by centrifugation. Subsequent analysis was performed per the Medilunbio kit protocol: staining was conducted with propidium iodide (PI) and Annexin V-FITC. After 15 min incubation, samples were analyzed by flow cytometry.

### 2.6. Cell Cycle Assay

HCC1569 cells (1 × 10^5^/well) were seeded in 6-well plates and cultured for 24 h at 37 °C under 5% CO_2_. MY-14 at varying concentrations were added, followed by 5-day incubation. Cells were trypsinized, pelleted by centrifugation, and fixed with 70% ethanol. Subsequent staining was performed with propidium iodide (PI) and RNase A according to the Beyotime kit protocol. After 30 min incubation at 37 °C, samples were analyzed by flow cytometry.

### 2.7. Molecular Docking

Molecular docking was performed using Glide 6.1. The crystal structure of 8D6E was retrieved from the RCSB Protein Data Bank. Briefly, water molecules were removed from the protein structure, followed by energy minimization. Ligand 3D conformations were generated, a grid file was constructed, and docking was executed [20].

### 2.8. Molecular Dynamics Simulation

Molecular dynamics (MD) simulations were conducted using Desmond (Schrödinger). The docked complex was solvated in a TIP3P water box under the OPLS_4 force field. The system was neutralized with Na^+^/Cl^−^ counterions. Subsequent energy minimization was followed by a 100 ps equilibration MD simulation. Production MD was then performed under NPT ensemble conditions [21].

### 2.9. Binding Free Energy Calculations

Binding free energy calculations were performed using the corresponding built-in workflow scripts. The formulations were as follows:

$${∆G}_{bind}=∆G_{complex}-{(∆G}_{protein}+{∆G}_{ligand})$$

$${∆G}_{bind}={∆E}_{MM}-T∆S+∆G_{sol}$$

$${∆G}_{bind}={∆E}_{vdw}+{∆E}_{ele}+{∆G}_{GB}+{∆G}_{SA}-T∆S$$

The term ∆*G*_bind denotes the overall energy associated with PKMYT1. The quantities ∆*G*_complex, ∆*G*_protein, and ∆*G*_ligand indicate the free energies for the complex, the protein, and the ligand, respectively. The value of ∆*E*_MM reflects the interaction energy in the gas phase, which encompasses van der Waals forces (vdw), electrostatic forces or Coulomb interactions (ele), desolvation free energy (GB), and non-polar solvation energy (SA). Additionally, *T*∆S is derived from the alteration in conformational entropy resulting from ligand binding, while ∆*G*_sol refers to the energy associated with solvation.

### 2.10. Hirshfeld Surface Analysis

The final snapshot from MD simulations was subjected to Hirshfeld surface analysis utilizing Multiwfn 3.8 and Visual Molecular Dynamics (VMD) 1.9.3 [22,23].

### 2.11. Dynamic Cross-Correlation Matrix

Dynamic cross-correlation matrix (DCCM) analysis and MD trajectories of the MY-14–PKMYT1 complex and apo-PKMYT1 were processed via Desmond’s integrated scripts. Briefly, trajectory data were analyzed post-simulation to generate DCCM plots. Correlated motions of Cα atoms were evaluated to identify binding regions and elucidate local versus long-range residue coupling. The correlation coefficient (Cij), ranging from −1 to 1, quantified residue motion dynamics. Positive Cij values indicated concerted residue displacement in the same direction, while negative values denoted anti-correlated motion. These distinctions provided valuable insights into molecular interaction dynamics and structural behavior of the studied protein.

## 3. Results and Discussion

### 3.1. Chemistry Syntheses

**Scheme 1:**
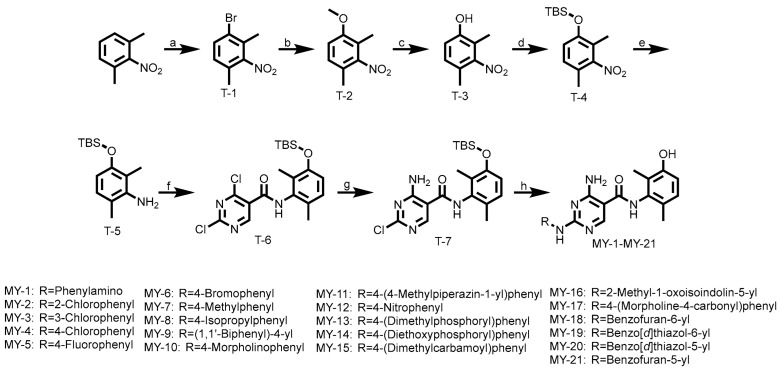
Reagent and Conditions: (a) Br_2_, FeBr_3_, Fe, CHCl_3_, 60 °C, 16 h, 53.6%; (b) MeONa, CuI, DMF, 80 °C, 6 h, 93.4%; (c) 2 M/L BBr_3_, DCM (dry), −40 °C, 2 h, 88.1%; (d) Trimethylchlorosilane, K_2_CO_3_, THF, r.t., 6 h, 74.7%; (e) Fe, NH_4_Cl, EtOH, 80 °C, 3 h, 89.1%; (f) 2,4-Dichloro-5-pyrimidinecarbonyl chloride, THF (dry), r.t., 4 h, 55.1%; (g) 1 M/L NH_3_·1,4-Dioxane, r.t., 2 h, 73.2%; (h) ArNH_2_, isopropanol: H_2_O = 3:1, CF_3_COOH, 90 °C, 2 h, 44.1–56.6%.

All compounds were synthesized according to the following route. As shown in Scheme 1, 2,6-Dimethylnitrobenzene underwent an electrophilic substitution reaction with bromine to yield T-1, which was treated with sodium methoxide under basic conditions via nucleophilic substitution, yielding intermediate T-2. Demethylation of T-2 using boron tribromide afforded T-3, followed by *tert*-butyldimethylsilyl (TBS) protection under basic conditions to produce T-4. Subsequent reduction with iron/ammonium chloride generated intermediate T-5. Condensation of T-5 with acyl chloride resulted in intermediate T-6, which underwent nucleophilic substitution with aqueous ammonia under basic conditions to afford T-7. Final coupling with various aniline derivatives provided target compounds MY-1 to MY-21.

### 3.2. Structural Optimization of PKMYT1 Inhibitors

In our compound library, M1 exhibited moderate kinase inhibitory activity (IC_50_ = 0.22 μM). Comparative molecular docking against the Phase II clinical candidate RP-6306 revealed near-identical binding modes at the DFG motif, where both compounds established hydrogen bonds with Asp251 and π–cation interactions involving Lys139. Within the hinge region, both engaged Cys190 via H-bonding. Crucially, RP-6306 established an additional H-bond with Glu188 through its amino group—a feature absent from M1—which mechanistically accounted for their 8.3-fold potency difference. Consequently, pharmacophore fusion was employed to introduce an amino group into M1, reinforcing hinge-region anchoring. This optimization yielded MY-1 with significantly enhanced inhibitory activity (IC_50_ = 0.026 μM).

**Figure 2:**
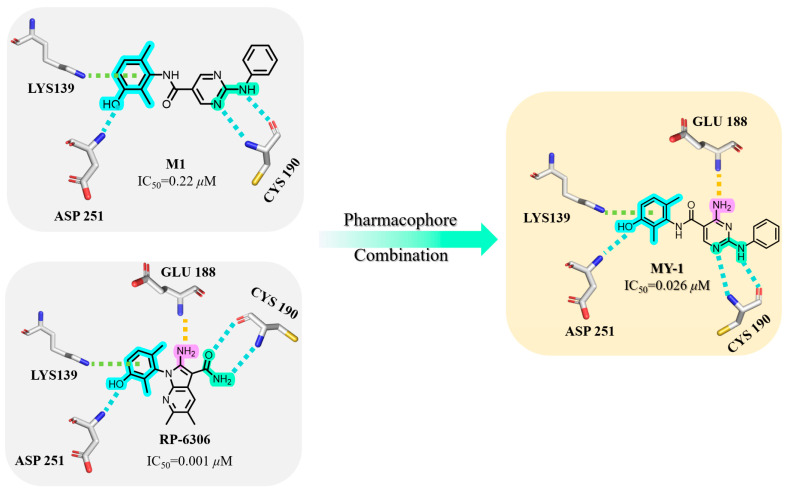
Rational Design of PKMYT1 Inhibitors through Pharmacophore Fusion.

As illustrated in Figure 2, MY-1 exhibited favorable binding at both the DFG motif and hinge region. Consequently, structural optimization was strategically directed toward the solvent-exposed R fragment. As shown in Table 1, initial modifications involved *ortho-*, *meta-*, and *para*-substitutions on the phenyl ring, wherein the *p*-chloro analog MY-4 demonstrated optimal potency (IC_50_ = 0.016 μM). Subsequent fluorine/bromine introduction led to reduced activity, though without progressive decline with increasing steric bulk, indicating significant steric accommodation at this molecular position.

As shown in Table 2, hydrophilic moiety incorporation then yielded MY-10 (IC_50_ = 0.0086 μM) and MY-11 (IC_50_ = 0.007 μM), with potency enhancement mechanistically attributed to H-bond interactions from oxygen and protonated *N*-methylpiperazine, thereby enhancing target engagement. Further introduction of strong H-bond donors—nitro (0.0051 μM), dimethylphosphinyl (0.0079 μM), and diethylphosphinyl (0.0026 μM)—achieved substantial activity gains, with the diethylphosphinyl derivative showing 8-fold higher potency than MY-4.

Subsequently, diverse hydrogen-bond-accepting moieties were introduced *para*-positionally. Most derivatives maintained comparable potency. As shown in Table 3, the incorporation of carbonyl-containing fragments resulted in compounds MY-16 and MY-17 exhibiting enhanced activity, with IC_50_ values of 0.0061 μM and 0.0033 μM, respectively. In benzofused heterocyclic systems, 6-substituted analog consistently outperformed 5-substituted counterparts (e.g., 6-benzofuran IC_50_ = 0.0085 μM vs. 5-isomer 0.012 μM). Notably, benzothiazole derivatives achieved stronger potency than benzofuran analogs, attributable to enhanced sulfur-mediated H-bond acceptance.

### 3.3. Cell Anti-Proliferation Activity Assay

Compounds with IC_50_ values below 10 nM were further evaluated for their anti-proliferative activity against *CCNE1*-amplified HCC1569 (breast) and OVCAR3 (ovarian) cancer cells. As shown in the Table 4, MY-17 exhibited potent anti-proliferative activity in both cell lines, with IC_50_ values of 1.06 μM and 0.80 μM for HCC1569 and OVCAR3, respectively. In contrast, the structurally analogous compound MY-13 demonstrated a complete loss of activity, which was attributed to its extremely low calculated partition coefficient (*cLogP* = 0.59), likely compromising cellular membrane permeability and thereby impairing functional efficacy. In addition, variable anti-proliferative potencies among certain compounds were observed across both cell lines. As shown in Table 5, the optimized compound MY-14 was further tested in lung cancer cells A549, which expressed CCNE1 at low levels, as well as in the corresponding lung epithelial cells BEAS-2B. MY-14 did not exhibit cytotoxicity, whereas RP-6306 demonstrated potent activity with IC_50_ values of 1.45 and 2.60 μM in A549 and BEAS-2B, respectively. Consequently, MY-14 was prioritized as the preferred compound for subsequent studies.

### 3.4. Effects of MY-14 on Colony Formation

**Figure 3:**
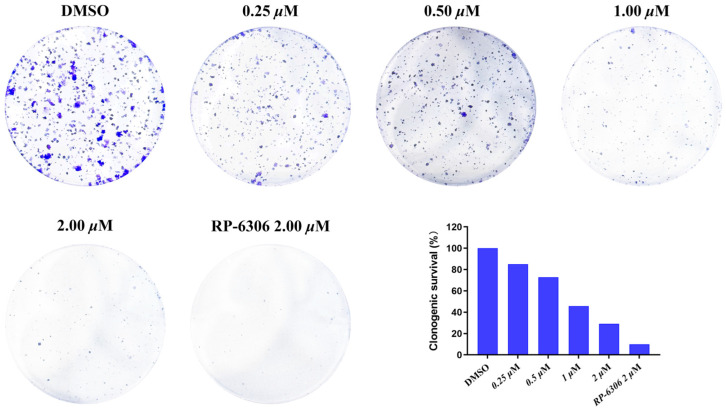
Cell Cloning Assay of Compound MY-14 in OVCAR3 Cells.

The colony formation assay served as a key indicator for evaluating cell proliferative capacity, invasiveness, and sensitivity to cytotoxic agents. HCC1569 cells demonstrated limited viability at low seeding densities; therefore, OVCAR3 cells were utilized for the colony formation inhibition assay. As shown in Figure 3, compound MY-14 inhibited colony formation in a dose-dependent manner following 14 days of incubation at varying concentrations.

### 3.5. Effects of MY-14 on Cell Apoptosis

**Figure 4:**
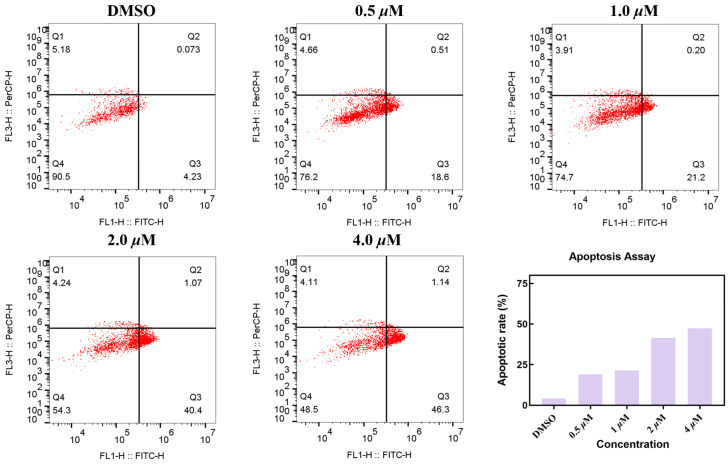
Apoptosis Assay of Compound MY-14 in HCC1569 Cells.

To elucidate the mechanism of action of compound MY-14, we assessed its ability to induce apoptosis in *CCNE1*-amplified breast cancer cells (HCC1569). As shown in Figure 4, compound MY-14 induced apoptosis in a concentration-dependent manner, with a maximal apoptotic rate of 47.44%. These results suggested that compound MY-14 promoted cell death by inducing the apoptotic pathway.

### 3.6. Effects of MY-14 on Cell Cycle

**Figure 5:**
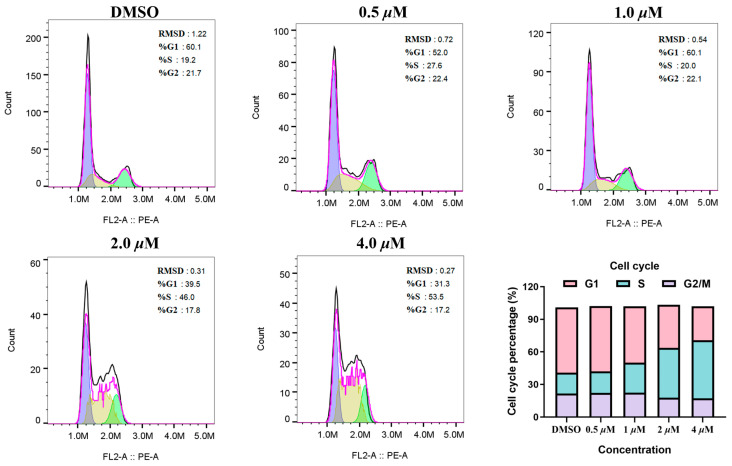
Cell-Cycle Analysis of Compound MY-14 in HCC1569 Cells.

The effect of MY-14 on the cell cycle was evaluated in HCC1569 cells. As illustrated in Figure 5, compound MY-14 induced a concentration-dependent increase in the proportion of S-phase cells while concurrently reducing the proportion of G1-phase cells. Conversely, it exhibited no significant impact on the distribution of G2/M-phase cells. Given that *CCNE1*, the synthetic lethal partner of PKMYT1, directly regulates the G1/S transition, these data further suggested that compound MY-14 induced G1/S phase cell-cycle arrest by inhibiting PKMYT1 and subsequently modulating *CCNE1*.

### 3.7. Molecular Dynamics Simulations

**Figure 6:**
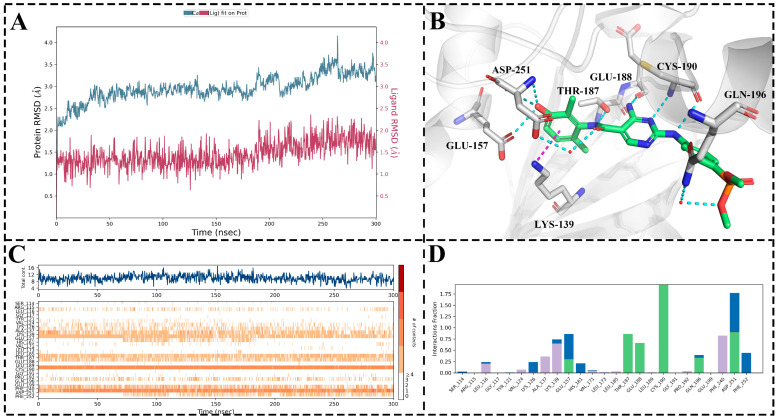
Molecular Dynamics Simulation of MY-14. (**A**) RMSD trajectories: Protein Cα atoms (blue); Ligand relative to protein (red). (**B**) Final snapshot conformation from MD simulation. (**C**) Interaction fingerprint (H-bonds, Hydrophobic, Ionic, Water bridges) across entire MD simulation. (**D**) Residue contact frequency histogram. The horizontal and vertical axes represent amino acids, while the vertical coordinate indicates the proportion of interactions between the amino acids and the ligands during the simulation process. A value greater than 1 indicates that an amino acid interacted with more than one atom of the ligand. In this context, green representes hydrogen bond interactions, blue denotes water-bridge interactions, and light purple signifies hydrophobic interactions.

Subsequently, molecular docking was initially conducted for MY-14. Due to the presence of conserved water molecules within the active site of PKMYT1, molecular docking could not reliably elucidate the binding mode of the compound; therefore, the resulting docked conformation was subjected to 300 ns molecular dynamics (MD) simulations. As illustrated in Figure 6A, the root-mean-square deviation (RMSD) of the complex remained below 2 Å throughout the MD simulation, indicating stable and valid simulation trajectories. As presented in Figure 6D, compound MY-14 maintained over 50% hydrogen bond occupancy with key residues Thr187, Glu188, Cys190, Gln196, and Asp251 and exhibited more than 50% hydrophobic interaction occupancy with Lys139 and Phe240. Figure 6C provides a corresponding visualization of the interactions shown in Figure 6D. Subsequently, the final snapshot from the MD simulation was extracted for 3D visualization. As depicted in Figure 6B, these interactions were highly consistent with those illustrated in Figure 6D. Notably, two conserved water molecules within the active site formed water-mediated hydrogen bonds with Asp251 and Gln196, respectively.

### 3.8. Binding Free Energy Calculation

**Figure 7:**
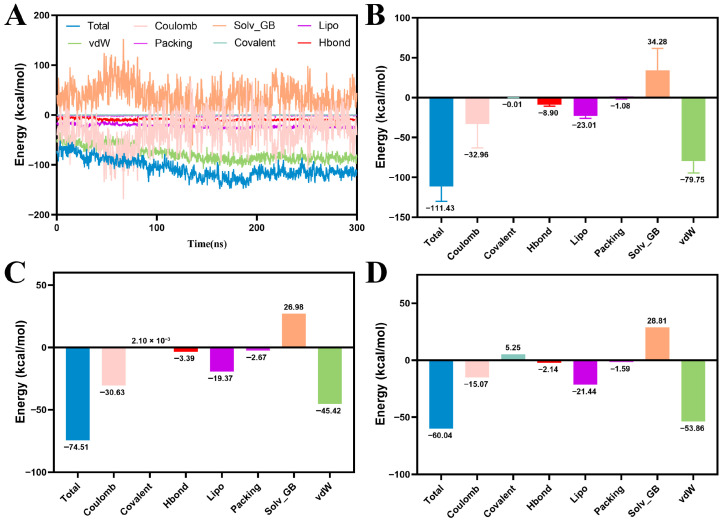
MM/GBSA Analysis of Protein-Ligand Complexes. (**A**) MM/GBSA energy variation over 300 ns MD simulation for PKMYT1-MY-14. (**B**) Histogram of MM/GBSA binding energy components for PKMYT1-MY-14. (**C**) Histogram of MM/GBSA binding energy components for PKMYT1-RP-6306. (**D**) Histogram of MM/GBSA binding energy components for PKMYT1-M1.

Next, the binding free energy for all MD trajectory-extracted conformations was calculated via MM/GBSA. As illustrated in the Figure 7, MY-14 exhibited significantly stronger binding affinity (–111.43 kcal/mol) compared to the lead compound (–60.04 kcal/mol) and the positive control compound (–74.51 kcal/mol). Decomposition analysis of the total binding energy (Figure 7B) indicated that van der Waals and electrostatic interactions were the primary contributors, followed by hydrophobic (Lipo) and hydrogen-bonding (HBond) interactions. Furthermore, solvation free energy (Solv) analysis revealed a substantial decrease in solvent-accessible surface area (SASA)-related energy upon formation of the ligand–receptor complex. Notably, the magnitude of this reduction exceeded that observed for both the lead (Figure 7D) and positive control compounds (Figure 7C), suggesting a superior binding affinity of MY-14 for PKMYT1.

### 3.9. Hirshfeld Surface Analysis of the Receptor–Ligand Interactions

**Figure 8:**
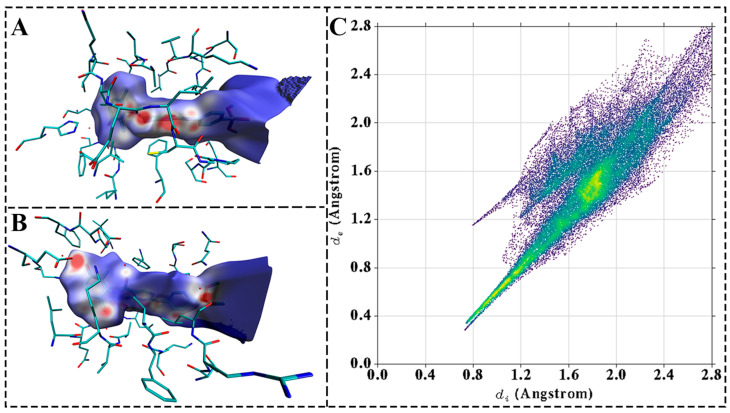
Hirshfeld surface and Fingerprint plots of PKMYT1-MY-14. (**A**) Hirshfeld surface of complex at 0° rotation. (**B**) Hirshfeld surface of complex at 180° rotation. (**C**) 2D fingerprint plot of complex. Hydrogen-bonding interactions appear as sharp spikes in the lower left region.

To elucidate the binding mode of the compound with the protein, quantum chemical methods were applied to perform Hirshfeld surface analysis and 2D fingerprint plot analysis of the active site within the complex. As illustrated in Figure 8A,B, the blue regions on the molecular surface—where electron density approached zero—indicated a lack of significant interactions. The white regions suggested the presence of potential π–π stacking or weak hydrogen-bonding interactions, while the red regions, characterized by higher electron density, corresponded to strong hydrogen-bonding interactions between the ligand and the receptor. These observations were consistent with the hydrogen bond-forming residues identified in molecular dynamics (MD) simulations, particularly Glu188, Cys190, and Asp251, which exhibited strong interaction intensities. Furthermore, Figure 8C displays characteristic sharp spikes in the lower left quadrant of the 2D fingerprint plot, indicative of hydrogen-bonding interactions. A total of five significant hydrogen bonds were identified, a finding that was corroborated by both Hirshfeld surface analysis and MD simulation data.

### 3.10. Dynamic Cross-Correlation Matrix Analysis for PKMYT1 Systems

**Figure 9:**
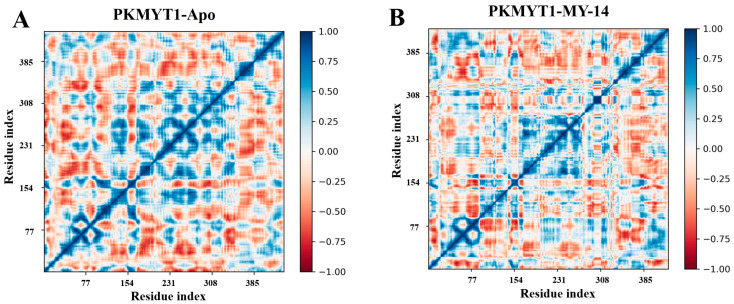
DCCM analysis for PKMYT1. (**A**) DCCM map of apo-PKMYT1 illustrating correlated motions in the absence of ligand. (**B**) DCCM map of the PKMYT1-MY-14 complex. Intensity of blue correlates with positive motion coupling; intensity of red indicates negative motion coupling. White regions denote no significant correlation.

Finally, dynamic cross-correlation matrix (DCCM) analysis was performed on the MD simulation trajectories. As shown in the Figure 9, dark blue regions indicated positively correlated motions between pairs of amino acid residues, whereas dark red regions represented negatively correlated motions. White regions denoted no significant correlations. The results demonstrated that key residues involved in PKMYT1 binding (Thr187, Glu188, Cys190, Asp251) significantly disrupted the collective motion patterns of neighboring residues. In contrast, residues located distal to the active site exhibited no significant perturbations.

## 4. Conclusions

In this study, compound M1 was identified as the lead compound. We developed the potent PKMYT1 inhibitor MY-14 through a pharmacophore fusion strategy, which exhibited significant kinase inhibitory activity (IC_50_ = 0.0026 μM) and cellular potency (IC_50_ = 1.02 μM in HCC1569 and 0.82 μM in OVCAR3 cells). MY-14 exhibited favorable cellular selectivity, showing reduced cytotoxicity against the tested *CCNE1*-low tumor cell line A549 as well as the corresponding lung epithelial cells. This performance was superior to that of the reference compound RP-6306. Pharmacological assays demonstrated that MY-14 induced apoptosis-mediated cell death, inhibited colony formation, and elicited G1/S phase cell-cycle arrest via synthetic lethality associated with *CCNE1*. Molecular dynamics simulations revealed hydrogen-bonding interactions between MY-14 and Thr187, Glu188, Cys190, and Asp251, along with a π–cation interaction involving Lys139 and a water-bridged hydrogen bond with Gln196. These findings were further corroborated by Hirshfeld surface analysis, fingerprint plots, and dynamic cross-correlation (DCCM) studies. MM/GBSA calculations indicated a binding free energy of –111.43 kcal/mol for MY-14, surpassing both the positive control and lead compound. Notably, in comparison with the currently most advanced inhibitor RP-6306, MY-14, while exhibiting comparable inhibitory activity at the enzymatic level and demonstrating acceptable safety, still displayed an order-of-magnitude difference at the cellular level. In the MD simulation of MY-14, it sustained stable interactions with the key amino acids described in the literature, further supporting the enzyme activity results. However, relative to RP-6306, MY-14 possessed a larger molecular weight and offered potential sites for structural modification, particularly at the amide bond, and modifications of MY-14 were currently underway. Collectively, MY-14 emerged as a promising compound for the development of a novel PKMYT1 inhibitor.

## Figures and Tables

**Table 1:** Inhibitory activities of compounds MY-1 to MY-9 against PKMYT1.

		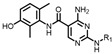			
**Compound**	**R**	**IC_50_ (** **μM) ^a^**	**Compound**	**R**	**IC_50_ (** **μM) ^a^**
MY-1	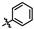	0.026	MY-6	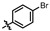	0.036
MY-2	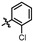	0.15	MY-7	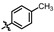	0.028
MY-3	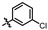	0.017	MY-8	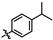	0.046
MY-4	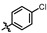	0.016	MY-9	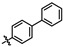	0.032
MY-5	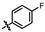	0.020	RP-6306	-	0.0099

^a^ The values of IC_50_ are shown as the mean (μM) values from two separate experiments.

**Table 2:** Inhibitory activities of compounds MY-10 to MY-14 against PKMYT1.

		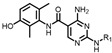			
**Compound**	**R**	**IC_50_ (** **μM) ^a^**	**Compound**	**R**	**IC_50_ (** **μM) ^a^**
MY-10	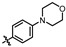	0.0086	MY-13	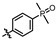	0.0079
MY-11	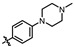	0.0071	MY-14	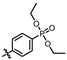	0.0026
MY-12	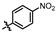	0.0051	RP-6306	-	0.0099

^a^ The values of IC_50_ are shown as the mean (μM) values from two separate experiments.

**Table 3:** Inhibitory activities of compounds MY-15 to MY-21 against PKMYT1.

		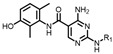			
**Compound**	**R**	**IC_50_ (** **μM) ^a^**	**Compound**	**R**	**IC_50_ (** **μM) ^a^**
MY-15	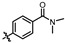	0.015	MY-19	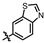	0.0035
MY-16	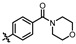	0.0061	MY-20	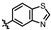	0.0057
MY-17	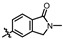	0.0033	MY-21	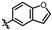	0.012
MY-18	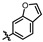	0.0085	RP-6306	-	0.0099

^a^ The values of IC_50_ are shown as the mean (μM) values from two separate experiments.

**Table 4:** Anti-Proliferative Activity of selected Compounds Against *CCNE1*-Amplified Tumor Cells.

Compound	R	PKMYT1 IC_50_ (μM) ^a^	HCC1569 IC_50_ (μM) ^b^	OVCAR3 IC_50_ (μM) ^b^
MY-10	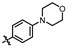	0.0086	2.95 ± 0.32	0.81 ± 0.06
MY-11	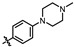	0.0071	3.56 ± 0.59	2.12 ± 0.33
MY-12	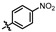	0.0051	6.35 ± 0.31	0.74 ± 0.11
MY-13	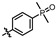	0.0079	>8.0	>8.0
MY-14	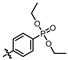	0.0026	1.06 ± 0.18	0.80 ± 0.07
MY-16	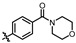	0.0061	>8.0	1.19 ± 0.09
MY-17	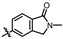	0.0033	>8.0	0.98 ± 0.07
MY-18	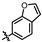	0.0085	6.97 ± 1.09	3.31 ± 0.18
MY-19	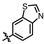	0.0035	3.14 ± 0.28	1.25 ± 0.06
MY-20	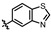	0.0057	3.54 ± 0.21	0.60 ± 0.09
RP-6306	-	0.0099	0.11 ± 0.02	0.09 ± 0.01

^a^ The values of IC_50_ are shown as the mean (μM) values from two separate experiments. ^b^ Data are expressed as means ± standard deviation (SD) (*n* = 3).

**Table 5:** The anti-proliferative activity of compounds in low-expressing *CCNE1* cells and normal cells.

Compound	A549 IC_50_ (μM) ^a^	BEAS-2B IC_50_ (μM) ^a^
MY-14	>8.0	>8.0
RP-6306	1.45 ± 0.23	2.60 ± 0.54

^a^ Data are expressed as means ± standard deviation (SD) (*n* = 3).

## Data Availability

The original contributions presented in this study are included in the article/Supplementary Materials. Further inquiries can be directed to the corresponding author.

## Supplementary Materials

The following supporting information can be downloaded at: https://www.mdpi.com/article/10.3390/biomedicines13092116/s1. All of Spectroscopic data for all Intermediates and compounds.
