# Supplementary material for: Design, Synthesis, and Computational Insights into PKMYT1 Inhibitors for the Treatment of Breast Cancer
Source: Biomedicines. 2025 Aug 29;13(9):2116. doi: 10.3390/biomedicines13092116 (PMC12467068; doi:10.3390/biomedicines13092116)
Supplement: Supplementary file 1 [file biomedicines-13-02116-s001.zip › biomedicines-3799234-supplementary.pdf]

Design, Synthesis and Computational Insights into PKMYT1 Inhibitors for the Treatment of Breast Cancer

Jinyu Yu[a#], Haoyu Zhang[a#], Chuanxu Su[a], Shizhe Yuan[a], Ziheng Yang[a], Nian Liu[a], Yixiang Sun[a], Zixuan Gao[a], Dongmei Zhao[a]*, Maosheng Cheng[a]

[a] *Key Laboratory of Structure-Based Drug Design and Discovery, Ministry of Education, School of Pharmaceutical Engineering, Shenyang Pharmaceutical University, 103 Wenhua Road, Shenhe District, Shenyang 110016, PR China*

*Corresponding author: Prof. Dongmei Zhao, medchemzhao@163.com.

#J.Y. and H.Z. are co-first authors and contributed equally to this work.

All of Spectroscopic data for all Intermediates and compounds.

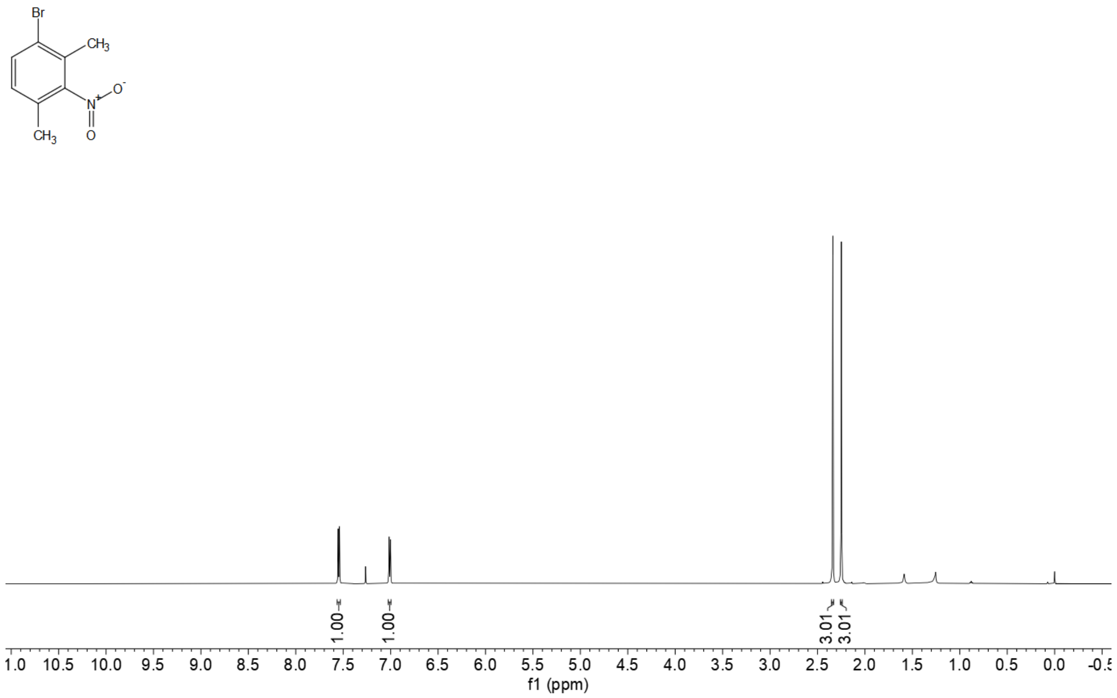

Figure S1 The ^{1}HNMR spectrum of Intermediate T-1.

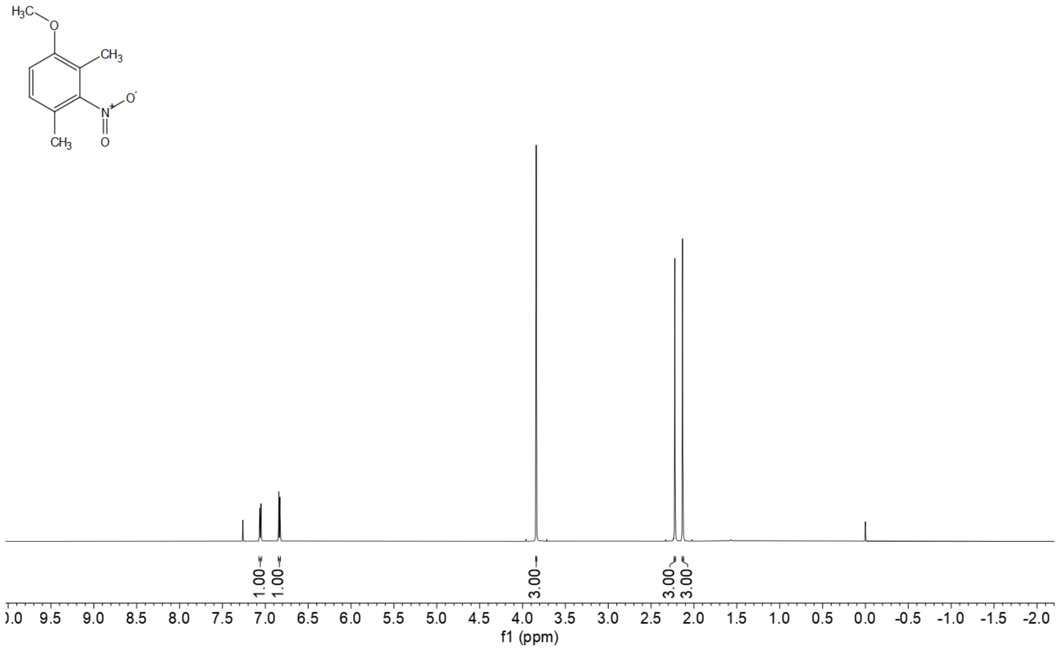

Figure S2 The ^{1}HNMR spectrum of Intermediate T-2.

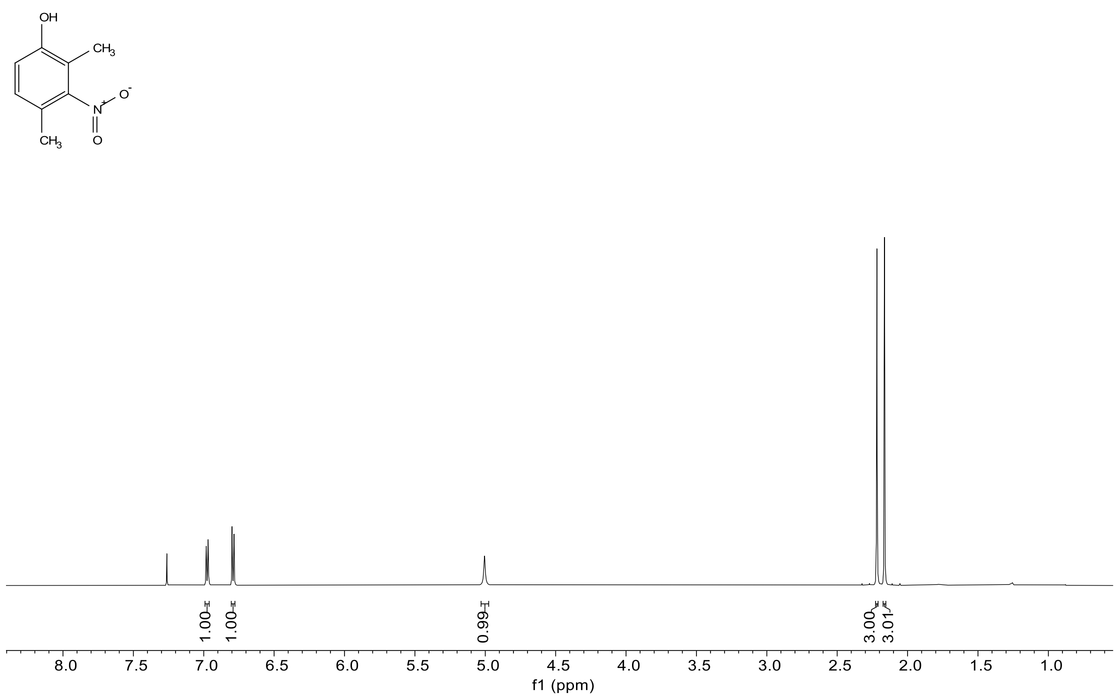

Figure S3 The ^{1}HNMR spectrum of Intermediate T-3.

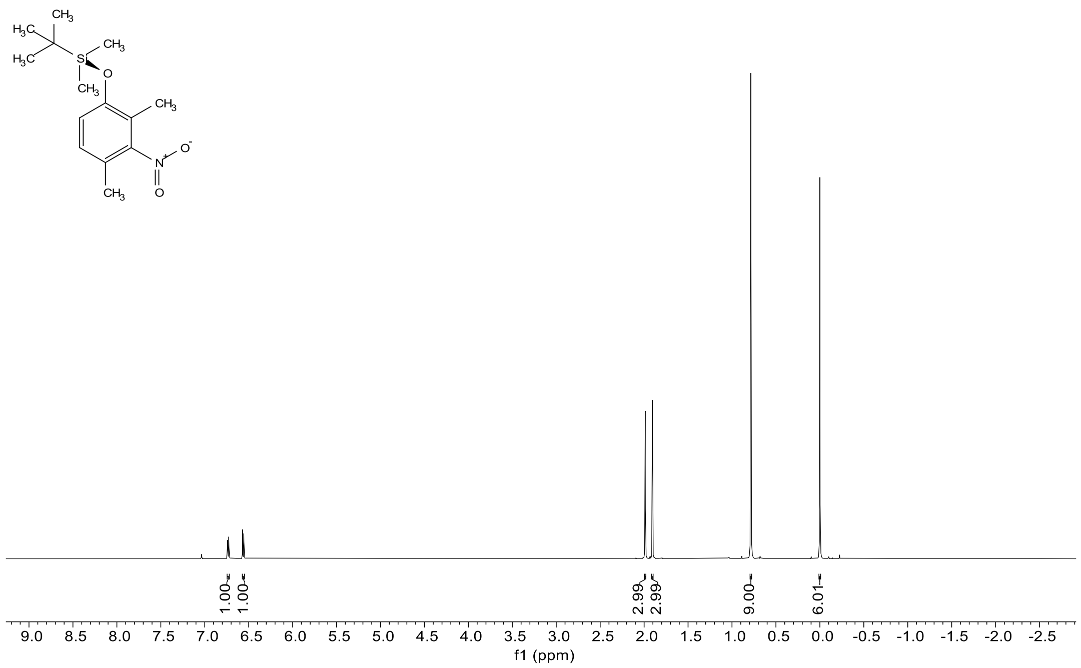

Figure S4 The ^{1}HNMR spectrum of Intermediate T-4.

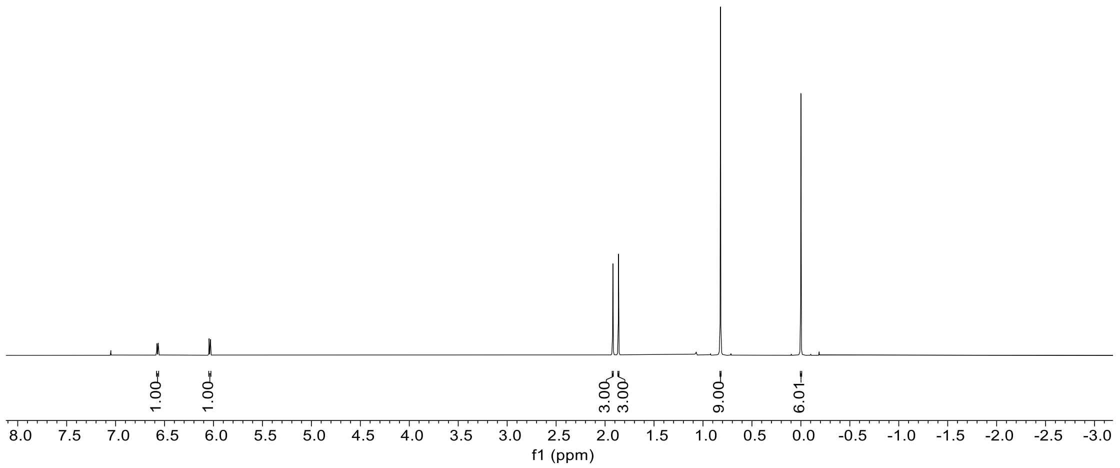

Figure S5 The ¹HNMR spectrum of Intermediate T-5.

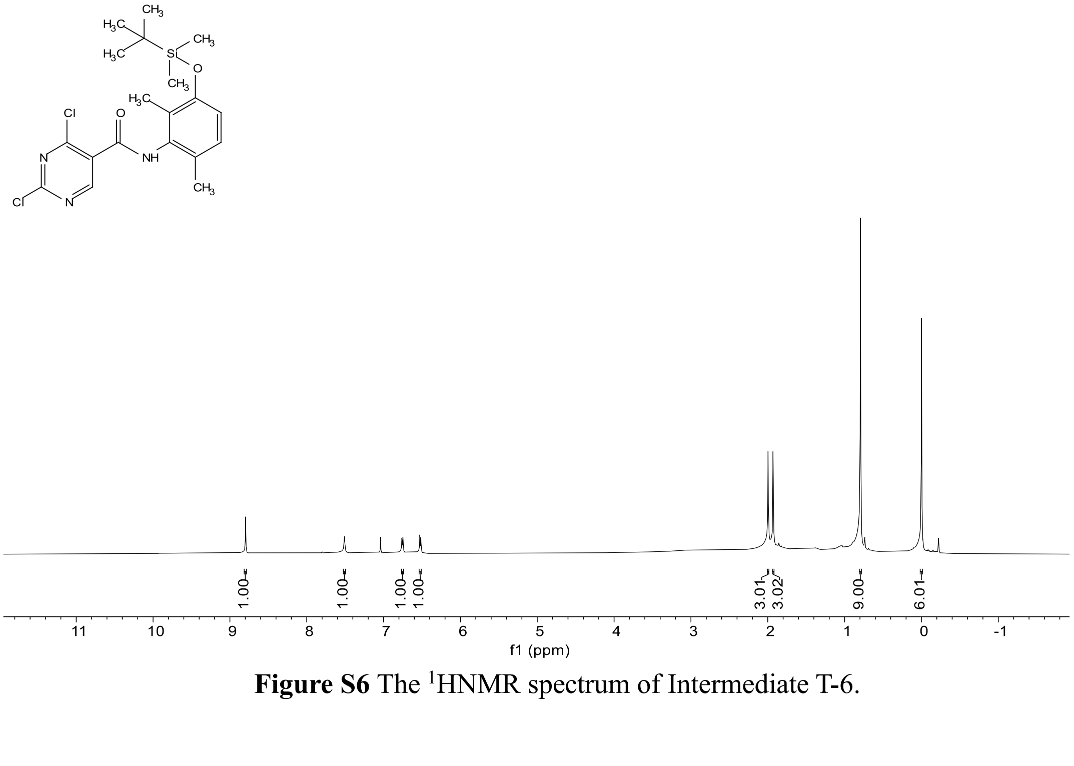

Figure S6 The ¹HNMR spectrum of Intermediate T-6.

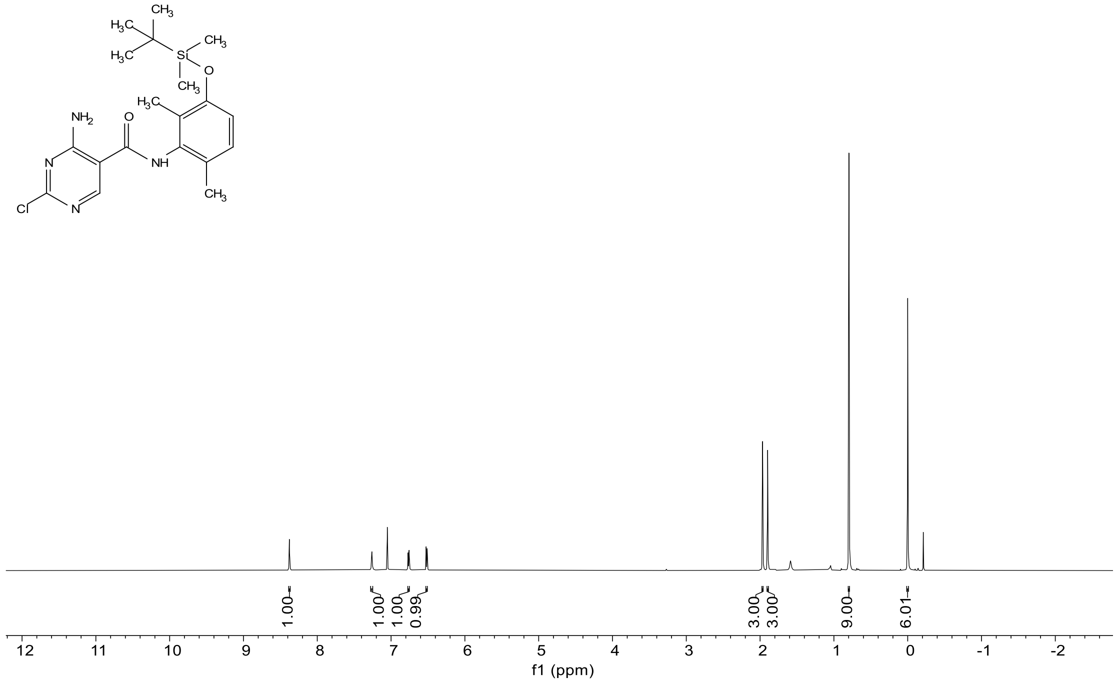

Figure S7 The ^{1}HNMR spectrum of Intermediate T-7.

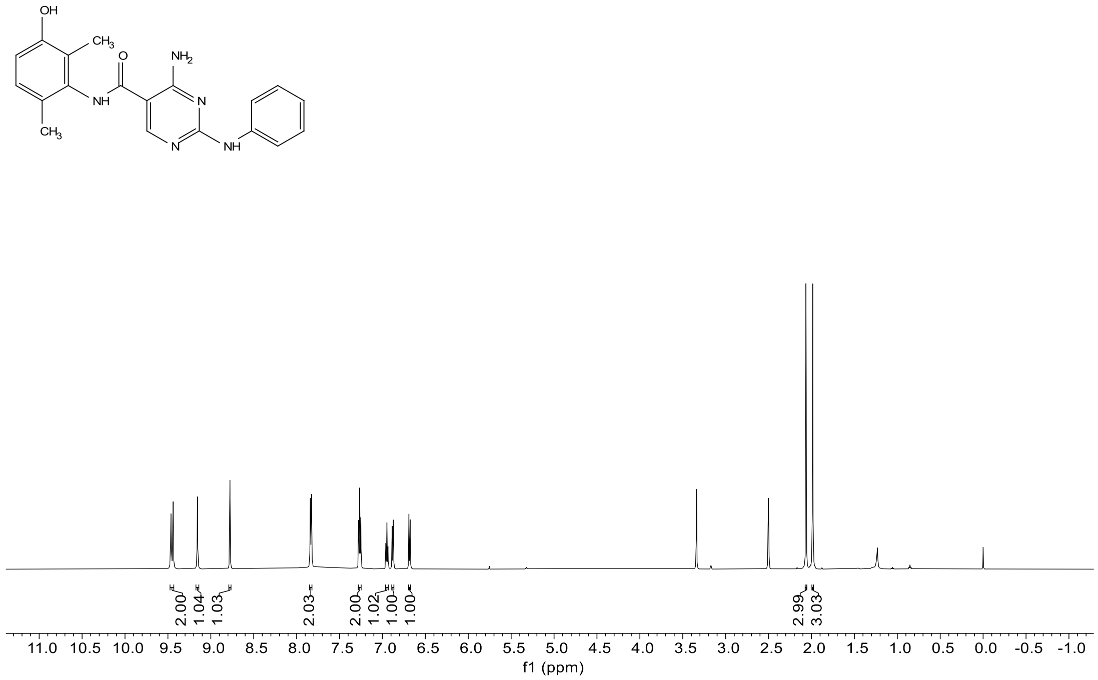

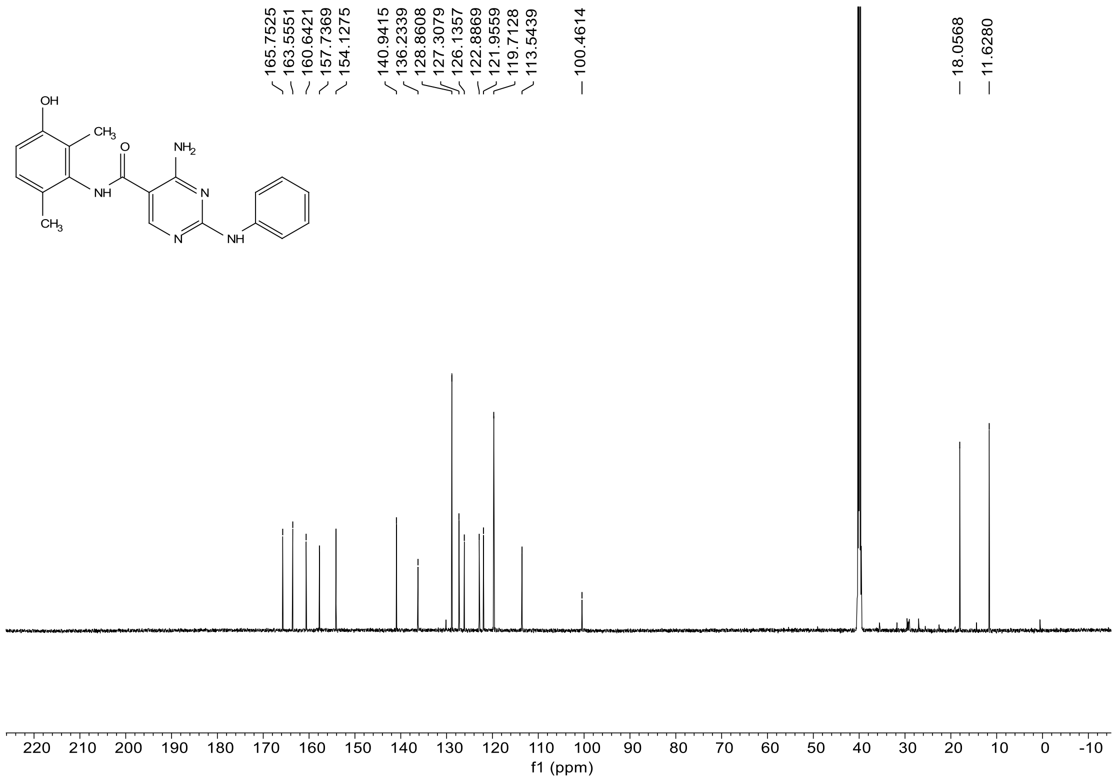

Fragmentor Voltage	Collision Energy	Ionization Mode
200	0	ESI

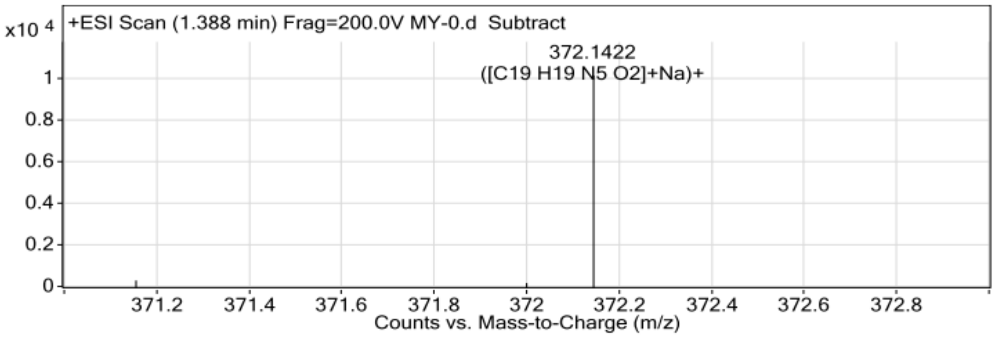

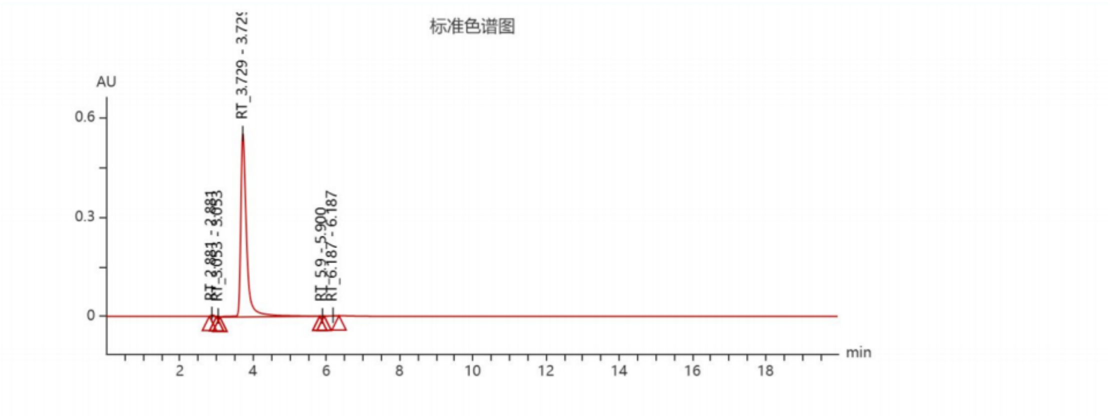

结果表

#	保留时间(min)	峰面积(μV*s)	峰高(μV)	百分面积(%)	百分高度(%)
1	2.881	25578.242	4209.727	0.413	0.750
2	3.053	7181.800	2737.375	0.116	0.488
3	3.729	6160499.058	554186.589	99.421	98.702
4	5.900	270.504	96.764	0.004	0.017
5	6.187	2850.190	242.131	0.046	0.043

Figure S8 The ^{1}HNMR, ^{13}CNMR, HRMS and HPLC spectrum of Compound MY-1.

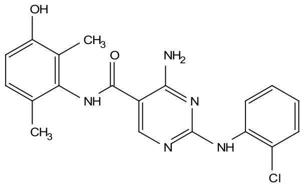

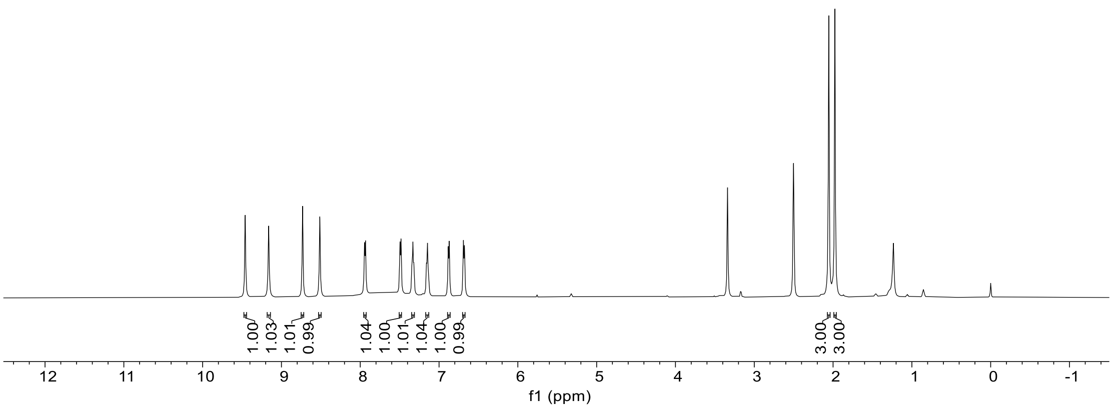

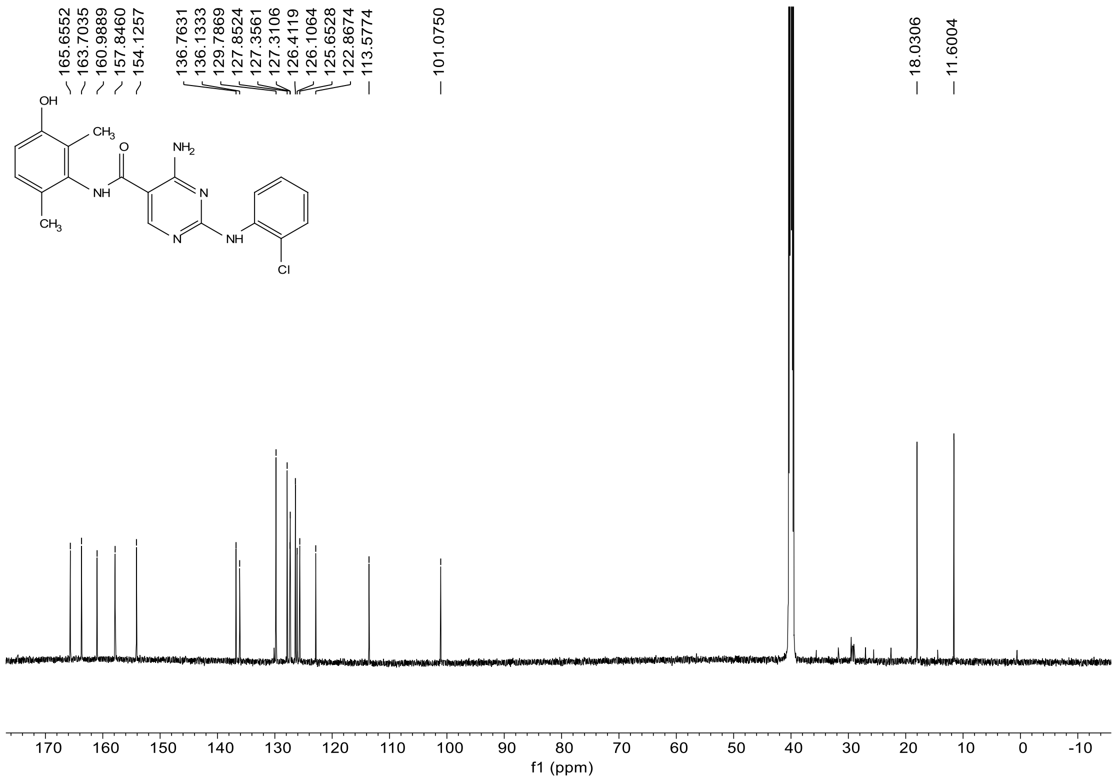
165.6552
163.7035
160.9889
157.8460
154.1257
136.7631
136.1333
129.7869
127.8524
127.3561
127.3106
126.4119
126.1064
125.6528
122.8674
113.5774
101.0750
18.0306
11.6004
OH
CH3
O
NH2
NH
N
N
NH
CH3
Cl
f1 (ppm)

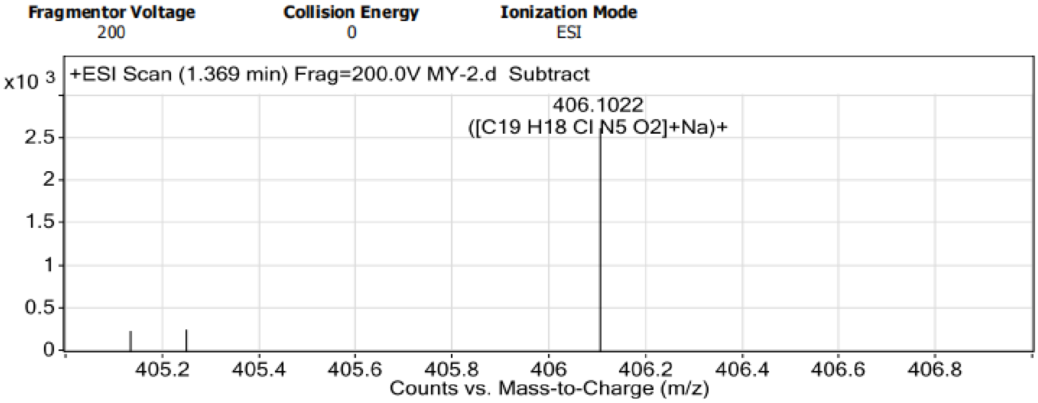
Fragmentor Voltage
200
Collision Energy
0
Ionization Mode
ESI
x10 3
+ESI Scan (1.369 min) Frag=200.0V MY-2.d Subtract
406.1022
([C19 H18 Cl N5 O2]+Na)+
Counts vs. Mass-to-Charge (m/z)

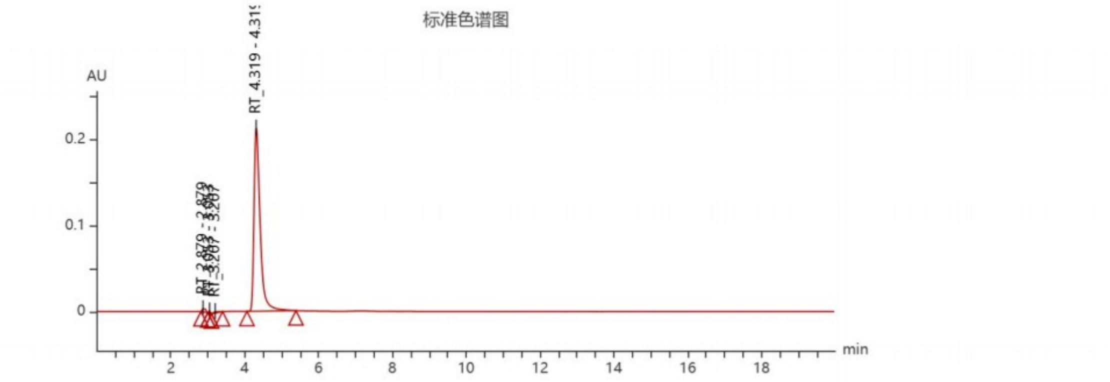

结果表

#	保留时间(min)	峰面积(μV*s)	峰高(μV)	百分面积(%)	百分高度(%)
1	2.879	25154.610	3828.987	1.005	1.735
2	3.053	6782.696	2669.817	0.271	1.210
3	3.207	16451.508	1542.250	0.657	0.699
4	4.319	2454218.050	212591.183	98.066	96.355

Figure S9 The ^{1}HNMR, ^{13}CNMR, HRMS and HPLC spectrum of Compound MY-2.

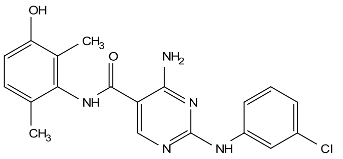

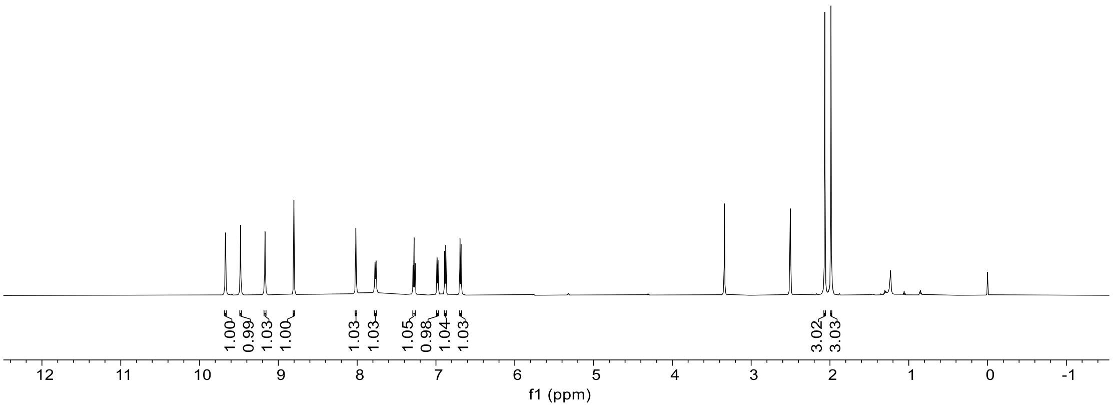

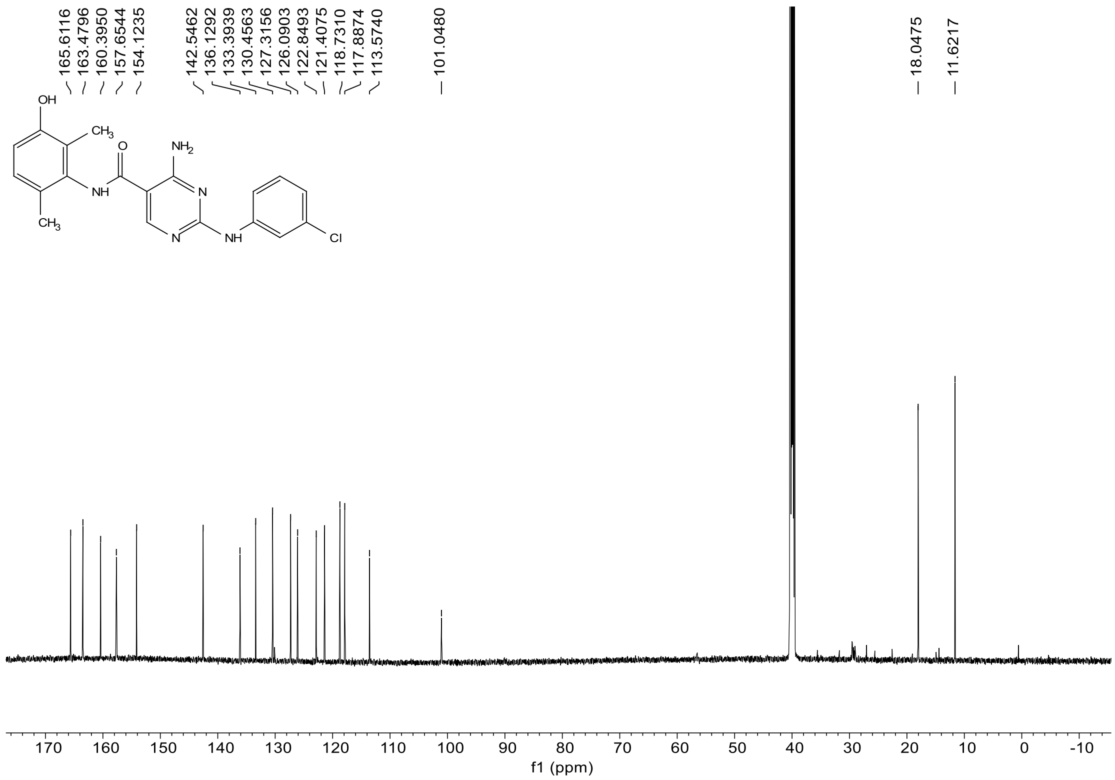

Fragmentor Voltage	Collision Energy	Ionization Mode
200	0	ESI

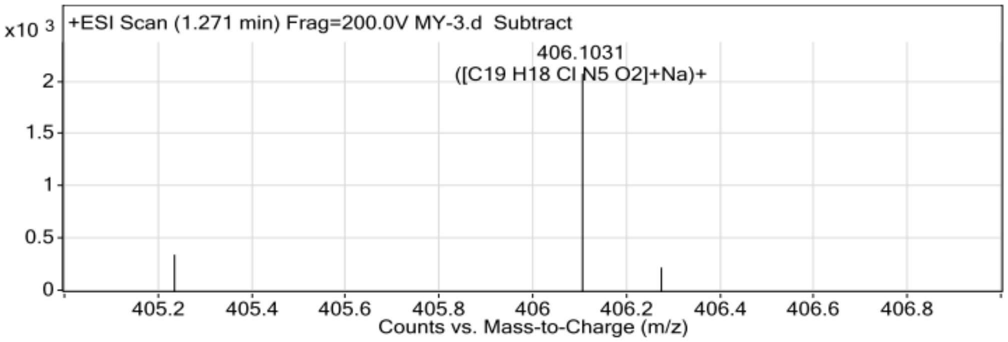

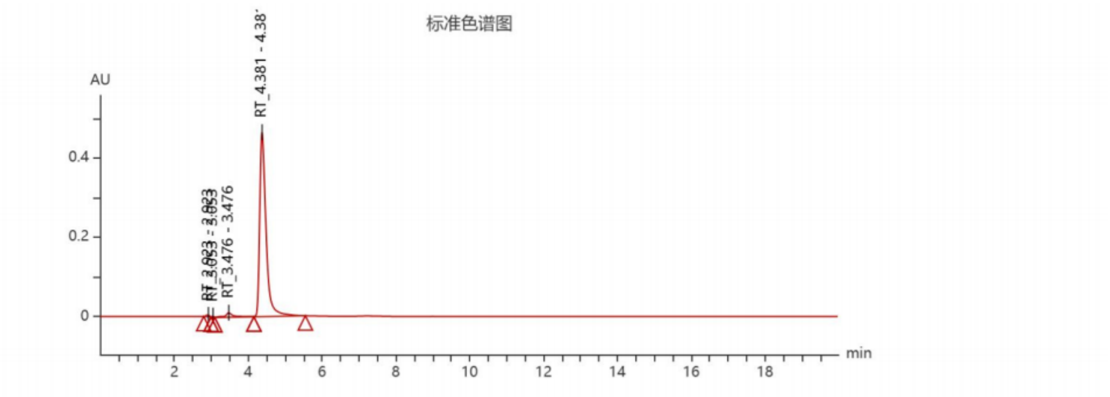

结果表

#	保留时间(min)	峰面积(μV*s)	峰高(μV)	百分面积(%)	百分高度(%)
1	2.923	27129.800	4299.378	0.470	0.890
2	3.053	7512.450	2782.000	0.130	0.576
3	3.476	193282.287	11005.692	3.347	2.279
4	4.381	5547489.380	464735.396	96.054	96.254

Figure S10 The ^{1}HNMR, ^{13}CNMR, HRMS and HPLC spectrum of Compound MY-3.

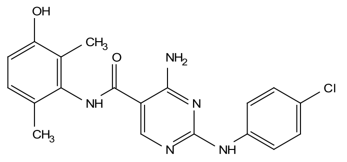

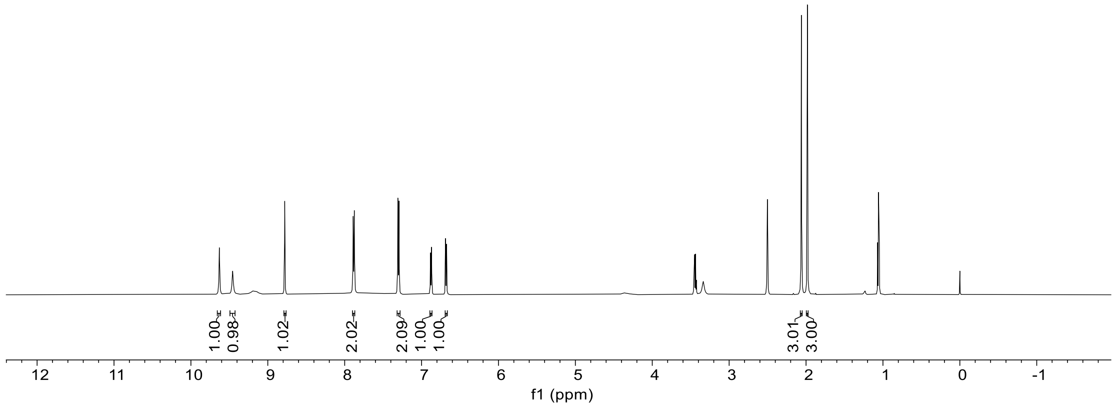

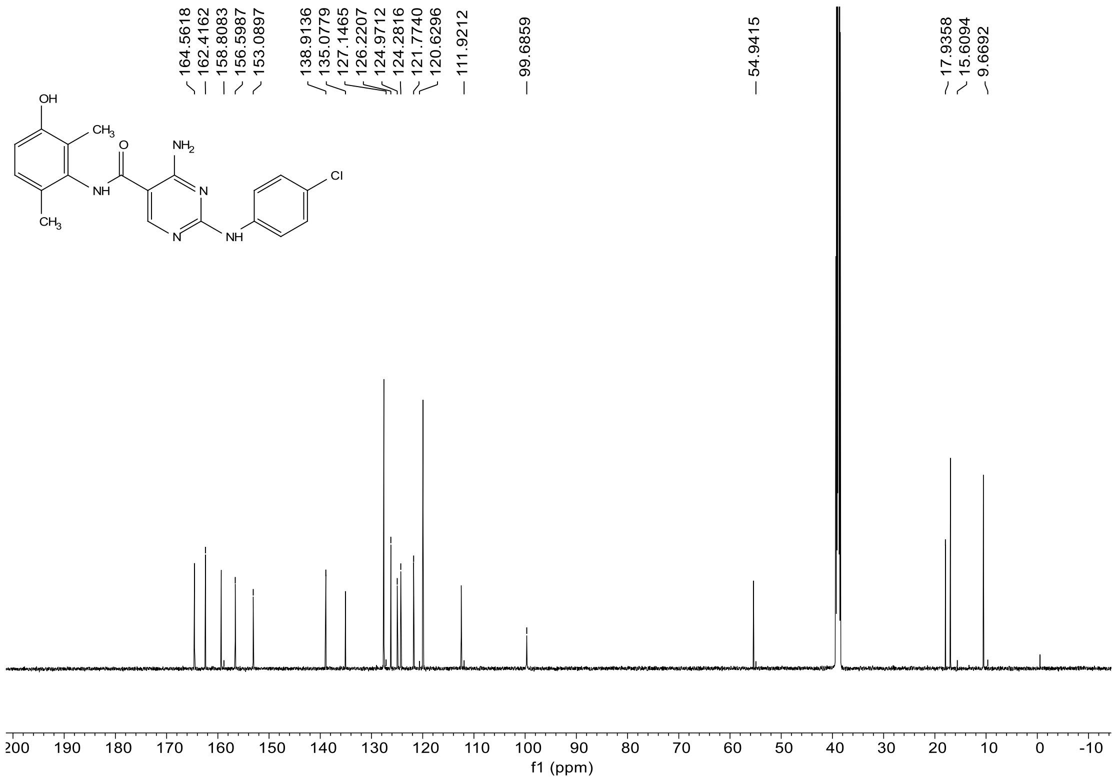

**Fragmentor Voltage**	**Collision Energy**	**Ionization Mode**
200	0	ESI

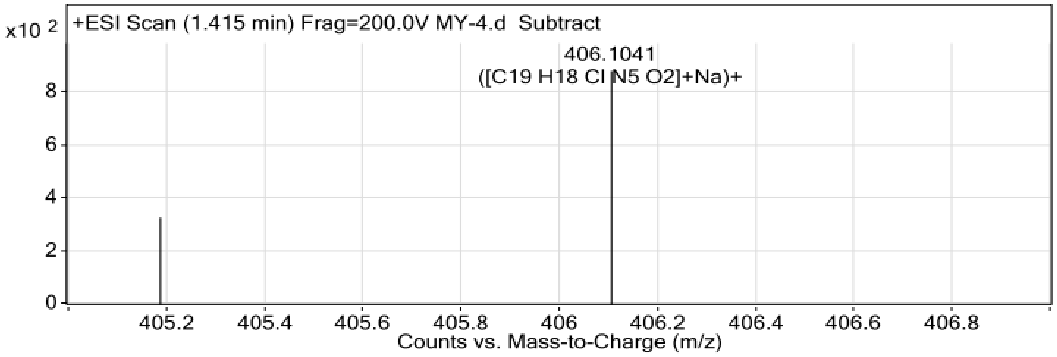

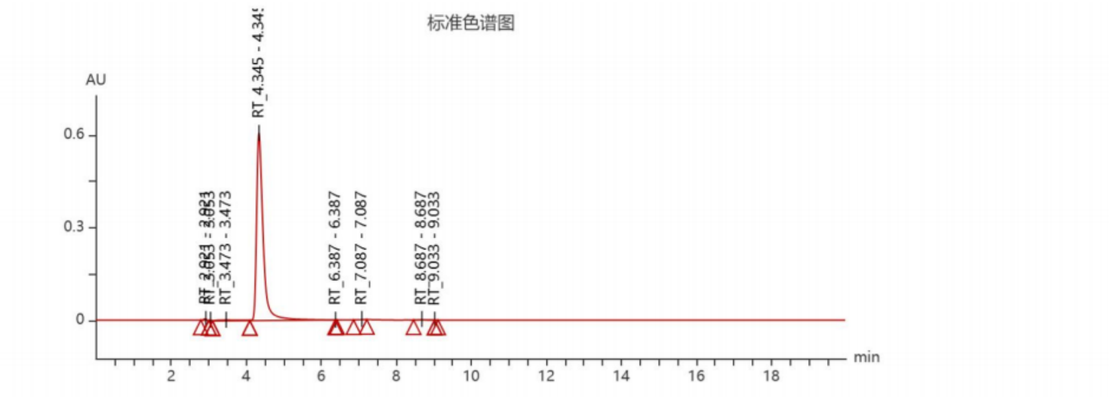

结果表

#	保留时间(min)	峰面积(μV*s)	峰高(μV)	百分面积(%)	百分高度(%)
1	2.921	26165.800	4262.346	0.344	0.688
2	3.053	6720.811	2627.917	0.088	0.424
3	3.473	139242.343	3471.298	1.831	0.561
4	4.345	7413496.873	607437.934	97.481	98.101
5	6.387	243.992	131.617	0.003	0.021
6	7.087	3290.325	297.557	0.043	0.048
7	8.687	15586.800	871.844	0.205	0.141
8	9.033	323.933	97.995	0.004	0.016

Figure S11 The ^{1}HNMR, ^{13}CNMR, HRMS and HPLC spectrum of Compound MY-4.

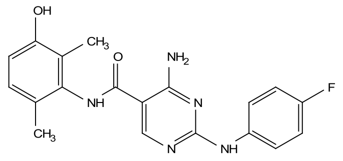

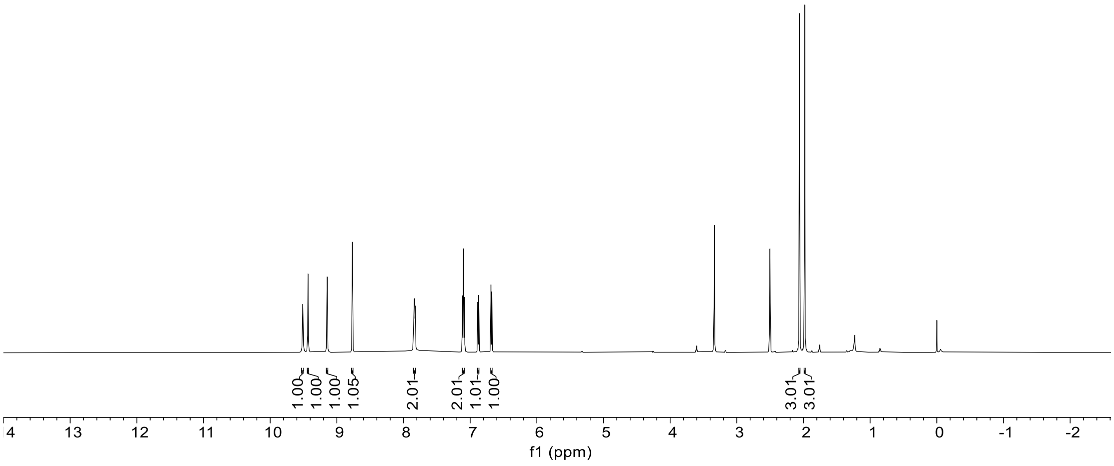

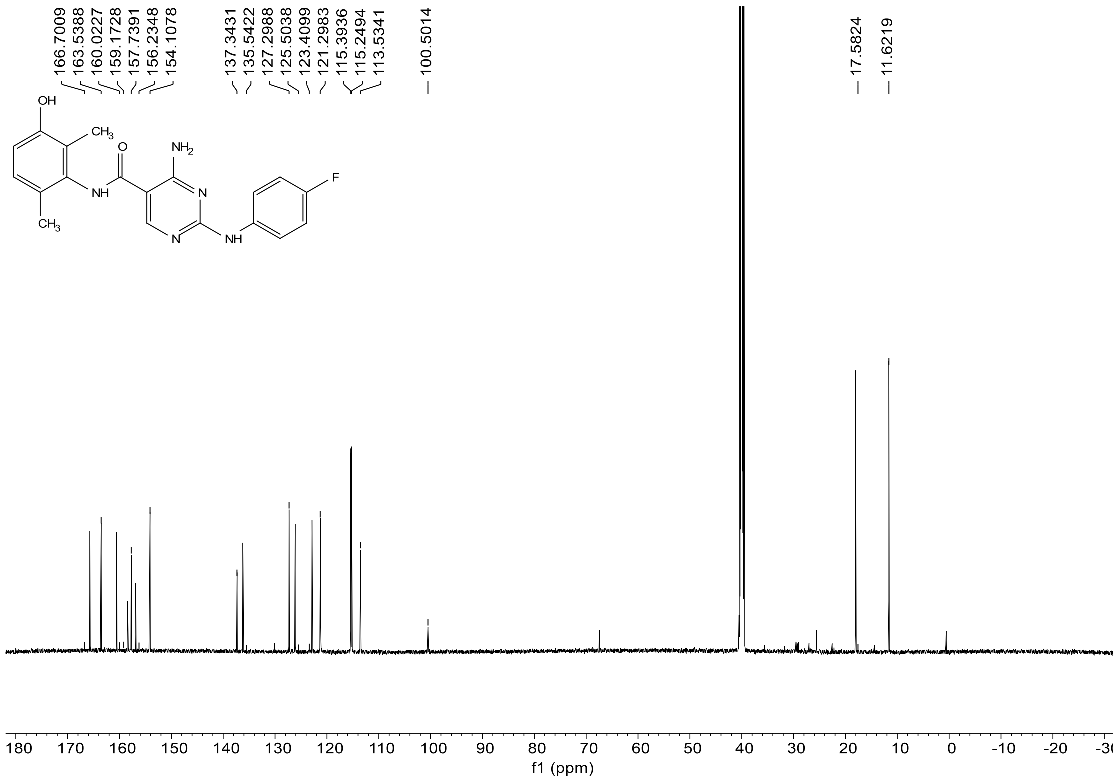

Fragmentor Voltage	Collision Energy	Ionization Mode
200	0	ESI

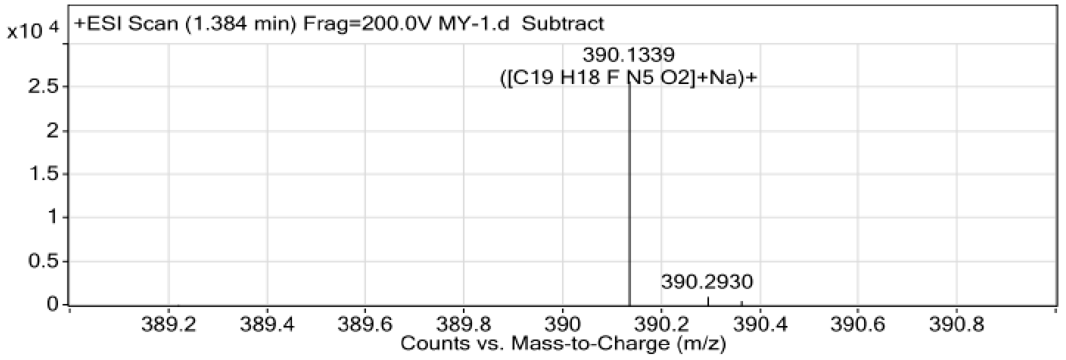

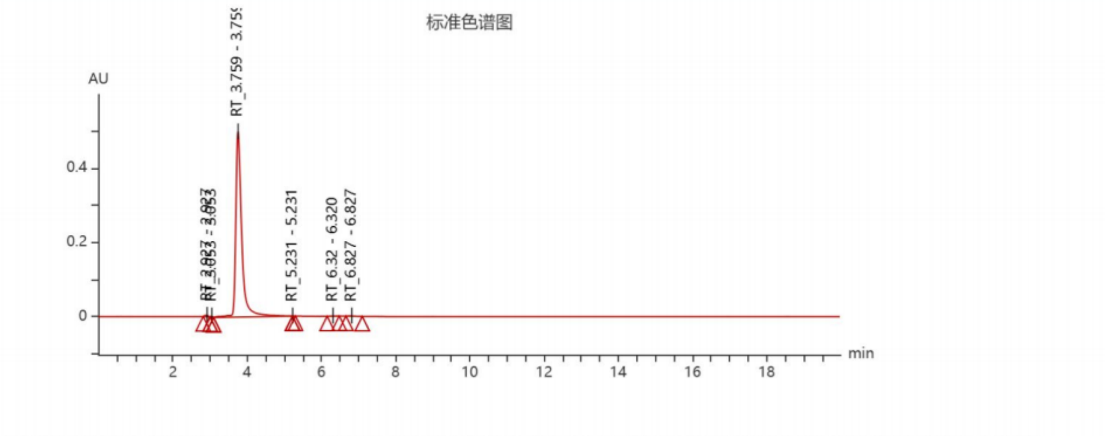

结果表

#	保留时间(min)	峰面积(μV*s)	峰高(μV)	百分面积(%)	百分高度(%)
1	2.927	24541.467	3768.464	0.433	0.742
2	3.053	6891.467	2675.409	0.122	0.527
3	3.759	5627135.387	500148.208	99.218	98.479
4	5.231	891.996	294.244	0.016	0.058
5	6.320	3402.967	313.046	0.060	0.062
6	6.827	8617.800	674.864	0.152	0.133

Figure S12 The ^{1}HNMR, ^{13}CNMR, HRMS and HPLC spectrum of Compound MY-5.

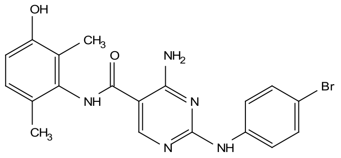

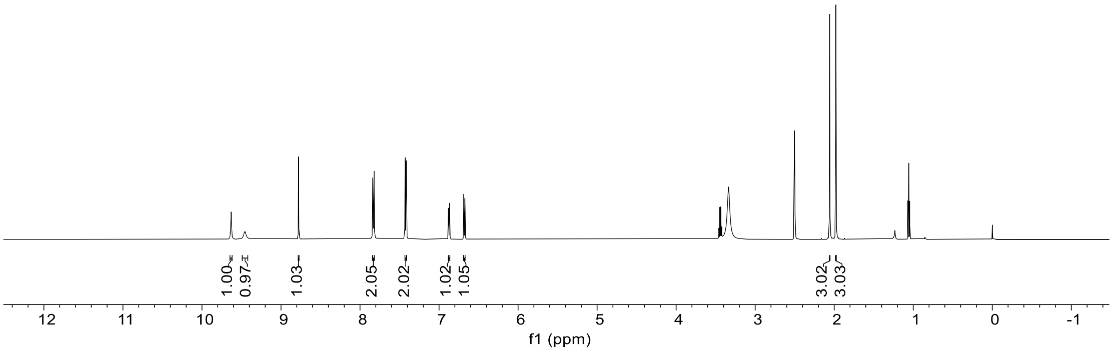

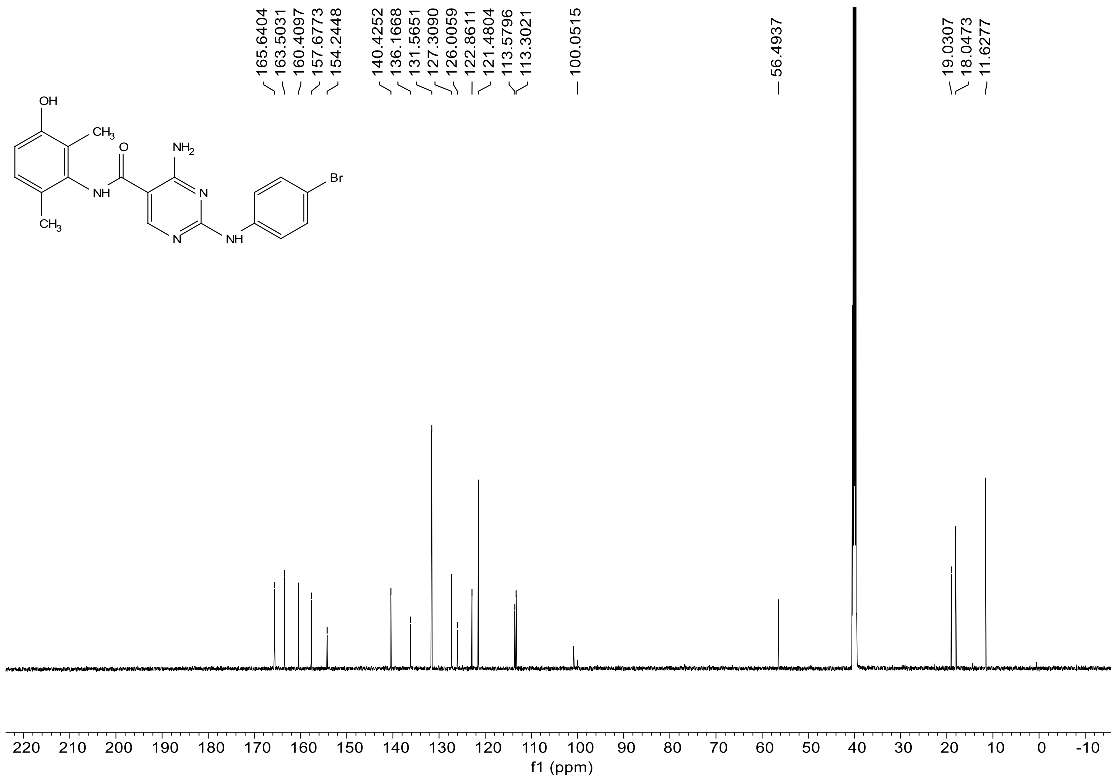

Fragmentor Voltage	Collision Energy	Ionization Mode
200	0	ESI

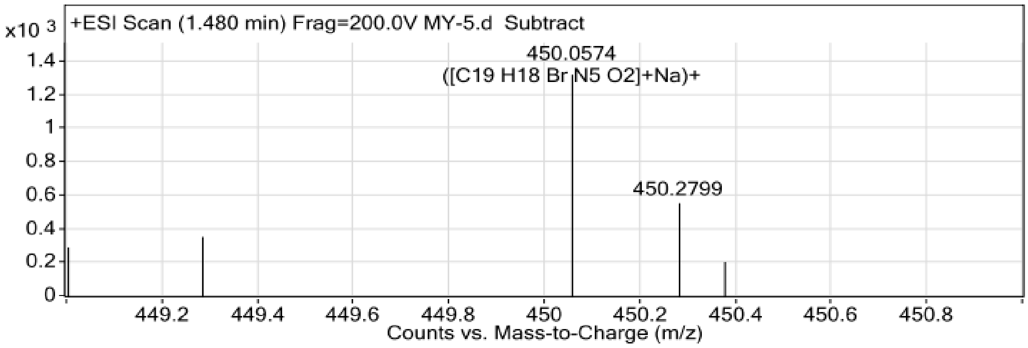

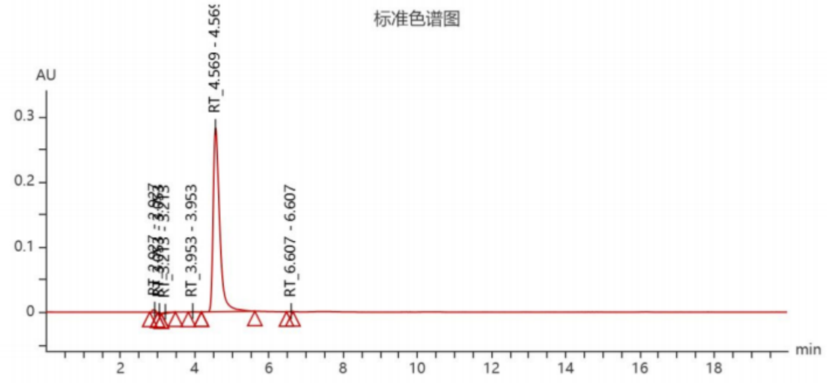

结果表

#	保留时间(min)	峰面积(μV*s)	峰高(μV)	百分面积(%)	百分高度(%)
1	2.927	26689.967	4008.406	0.763	1.375
2	3.053	6918.366	2684.233	0.198	0.921
3	3.213	22214.283	1674.553	0.635	0.575
4	3.953	3927.600	498.967	0.112	0.171
5	4.569	3436528.467	282376.210	98.250	96.887
6	6.607	1468.200	208.106	0.042	0.071

Figure S13 The ^{1}HNMR, ^{13}CNMR, HRMS and HPLC spectrum of Compound MY-6.

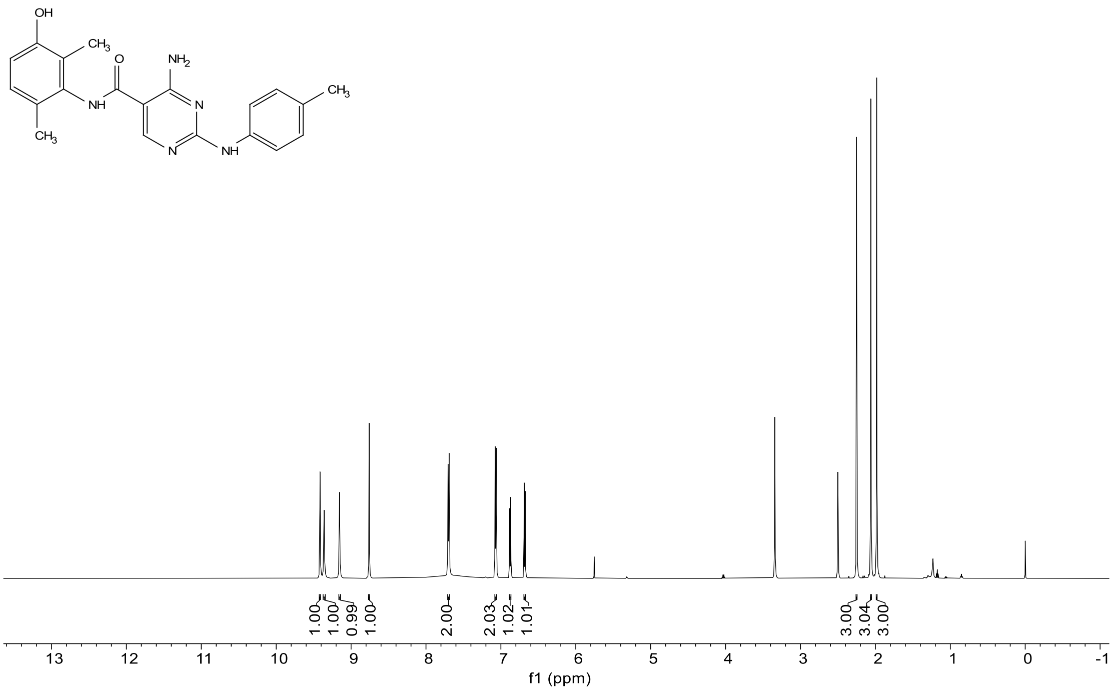

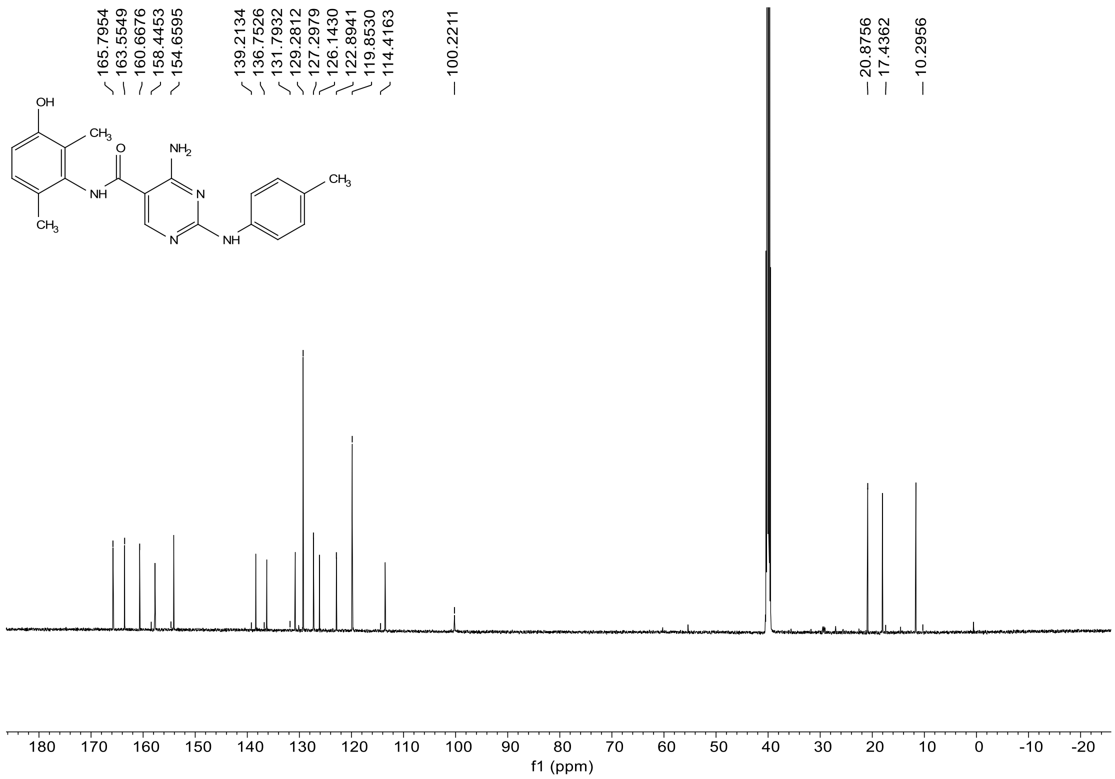

Fragmentor Voltage	Collision Energy	Ionization Mode
200	0	ESI

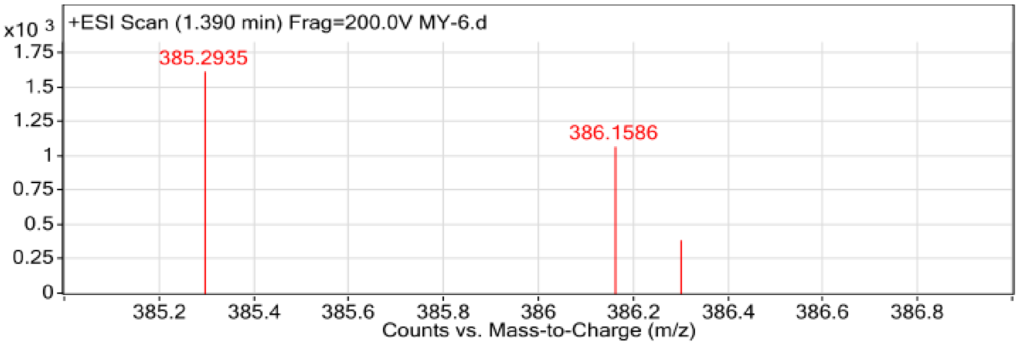

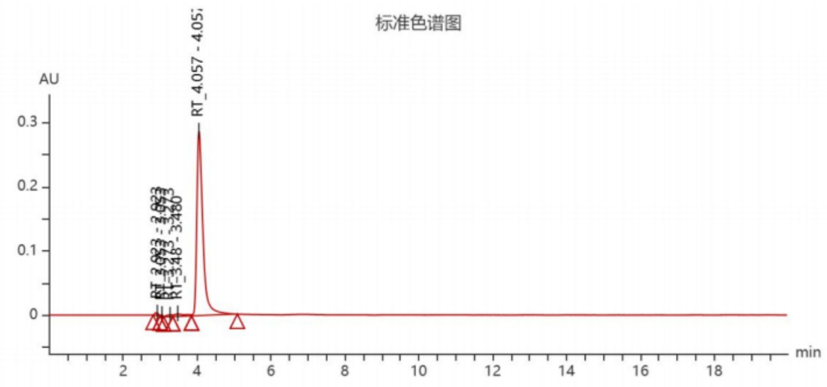

结果表

#	保留时间(min)	峰面积(μV*s)	峰高(μV)	百分面积(%)	百分高度(%)
1	2.923	24312.233	3846.099	0.715	1.283
2	3.053	6825.908	2574.233	0.201	0.859
3	3.273	29485.643	2593.652	0.867	0.865
4	3.480	76970.757	3745.680	2.262	1.250
5	4.057	3264619.841	286996.452	95.956	95.743

Figure S14 The ^{1}HNMR, ^{13}CNMR, HRMS and HPLC spectrum of Compound MY-7.

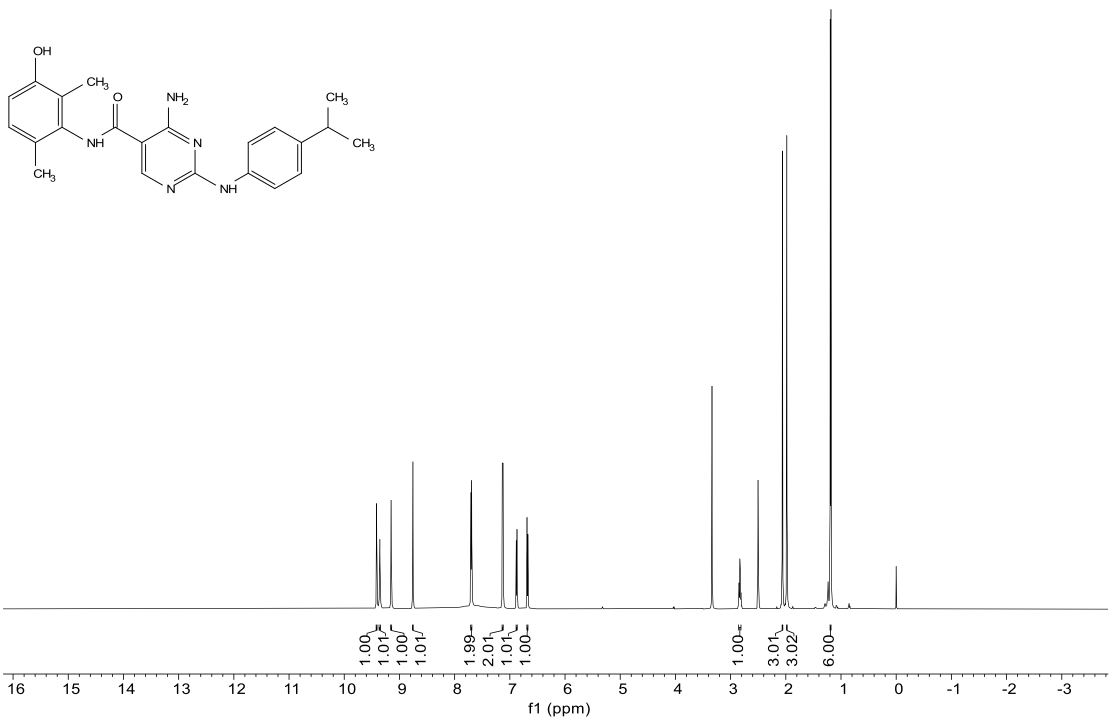

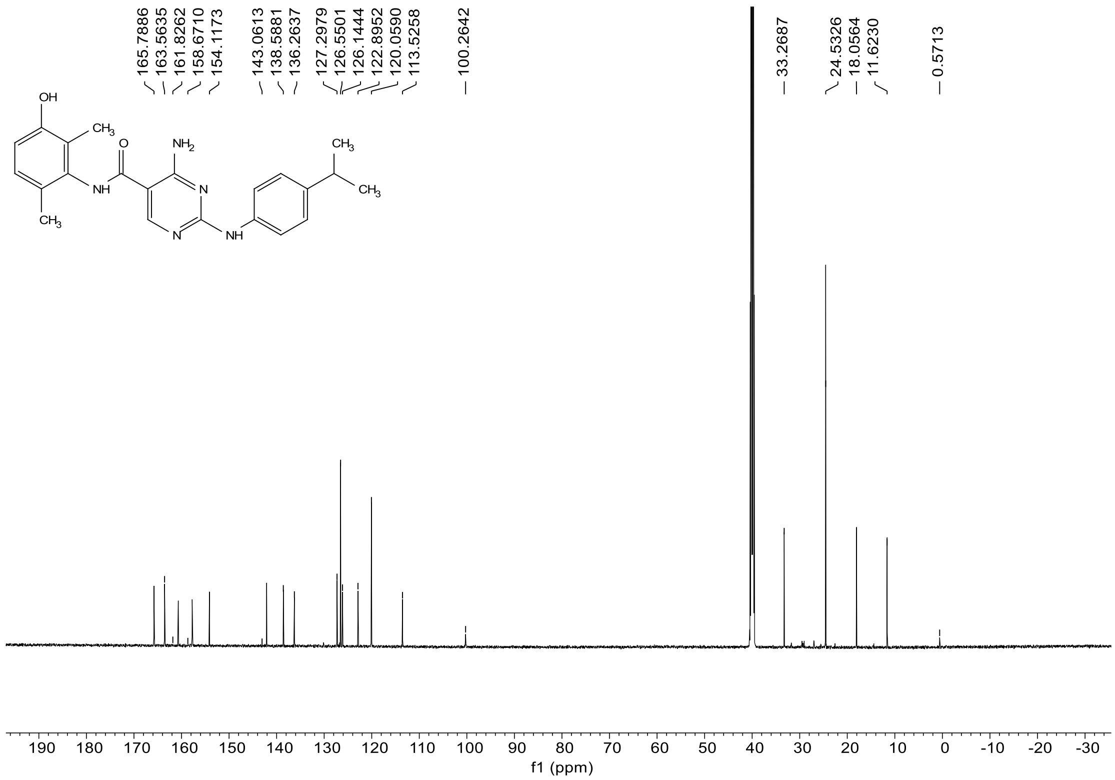
165.7886
163.5635
161.8262
158.6710
154.1173
143.0613
138.5881
136.2637
127.2979
126.5501
126.1444
122.8952
120.0590
113.5258
100.2642
33.2687
24.5326
18.0564
11.6230
0.5713
OH
CH3
O
NH2
N
NH
CH3
N
NH
CH3
CH3
f1 (ppm)

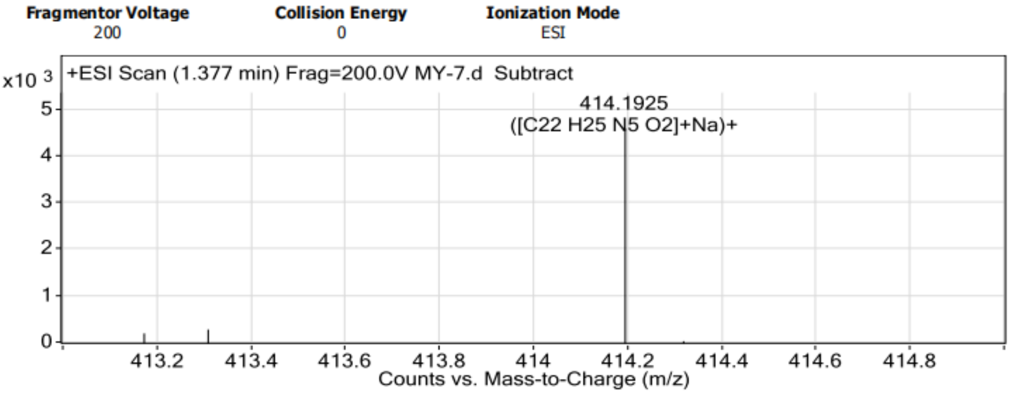
Fragmentor Voltage
200
Collision Energy
0
Ionization Mode
ESI
x10 3
+ESI Scan (1.377 min) Frag=200.0V MY-7.d Subtract
414.1925
([C22 H25 N5 O2]+Na)+
Counts vs. Mass-to-Charge (m/z)

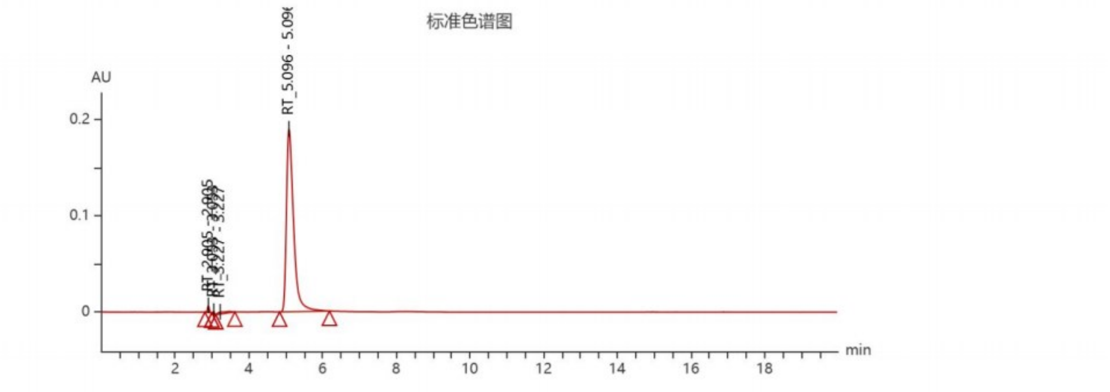

结果表

#	保留时间(min)	峰面积(μV*s)	峰高(μV)	百分面积(%)	百分高度(%)
1	2.905	23822.133	6714.052	0.907	3.341
2	3.053	6502.867	2545.500	0.248	1.267
3	3.227	39334.200	2009.038	1.497	1.000
4	5.096	2557321.433	189683.694	97.348	94.392

Figure S15 The ^{1}HNMR, ^{13}CNMR, HRMS and HPLC spectrum of Compound MY-8.

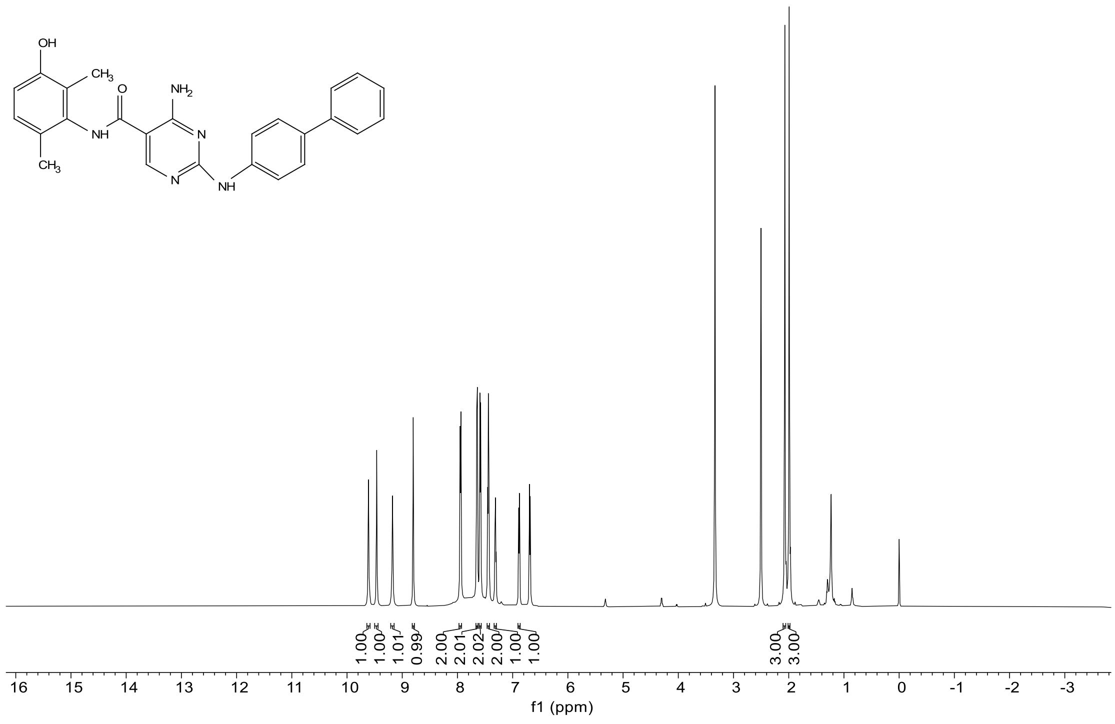

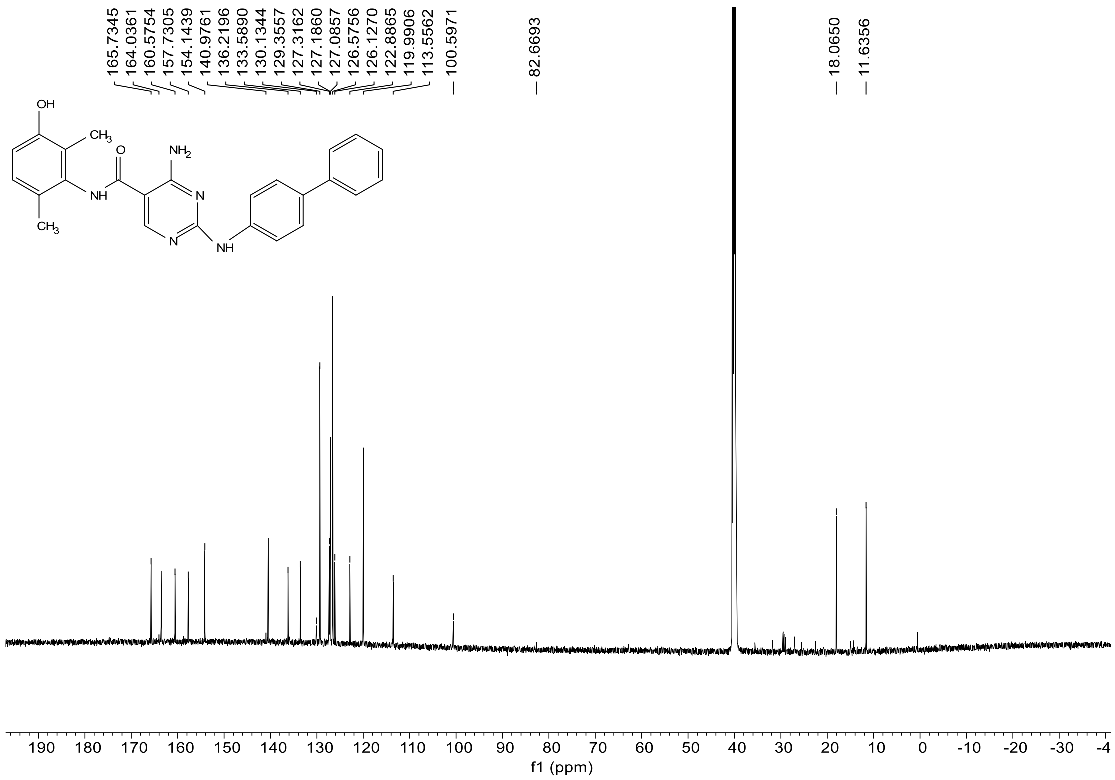
165.7345
164.0361
160.5754
157.7305
154.1439
140.9761
136.2196
133.5890
130.1344
129.3557
127.3162
127.1860
127.0857
126.5756
126.1270
122.8865
119.9906
113.5562
100.5971
82.6693
18.0650
11.6356
OH
CH3
O
NH2
NH
N
N
NH
CH3
190 180 170 160 150 140 130 120 110 100 90 80 70 60 50 40 30 20 10 0 -10 -20 -30 -4
f1 (ppm)

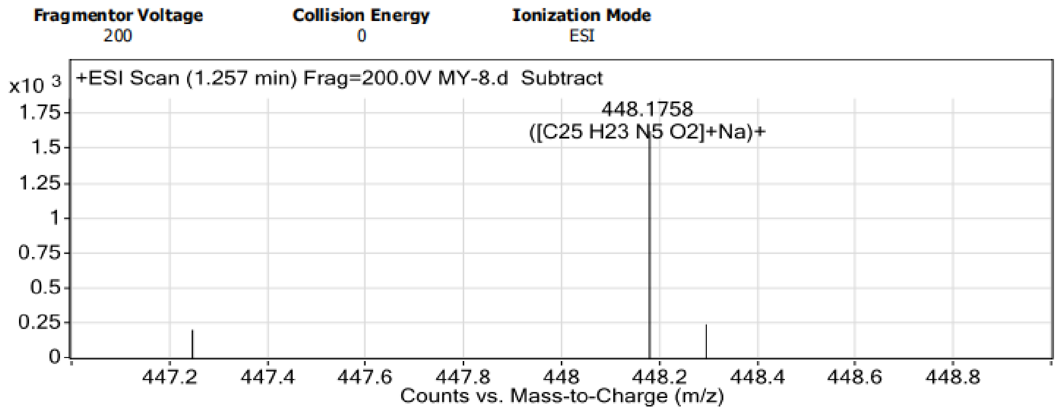
Fragmentor Voltage
200
Collision Energy
0
Ionization Mode
ESI
+ESI Scan (1.257 min) Frag=200.0V MY-8.d Subtract
x10 3
448.1758
([C25 H23 N5 O2]+Na)+
1.75
1.5
1.25
1
0.75
0.5
0.25
0
447.2 447.4 447.6 447.8 448 448.2 448.4 448.6 448.8
Counts vs. Mass-to-Charge (m/z)

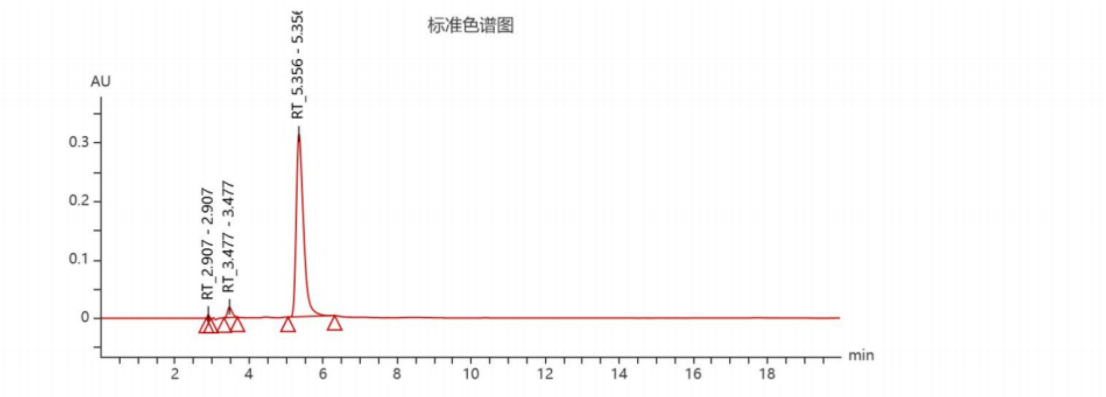

结果表

#	保留时间(min)	峰面积(μV*s)	峰高(μV)	百分面积(%)	百分高度(%)
1	2.907	20781.133	5892.861	0.467	1.751
2	3.477	153105.467	17696.577	3.437	5.259
3	5.356	4280341.733	312907.676	96.096	92.990

Figure S16 The ^{1}HNMR, ^{13}CNMR, HRMS and HPLC spectrum of Compound MY-9.

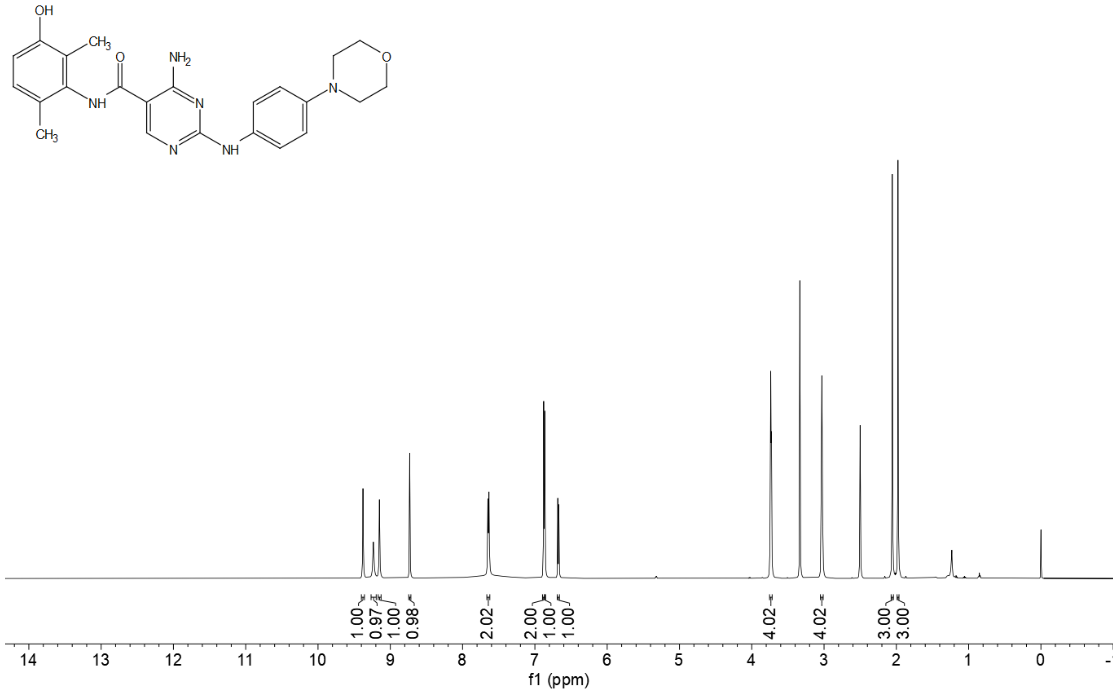

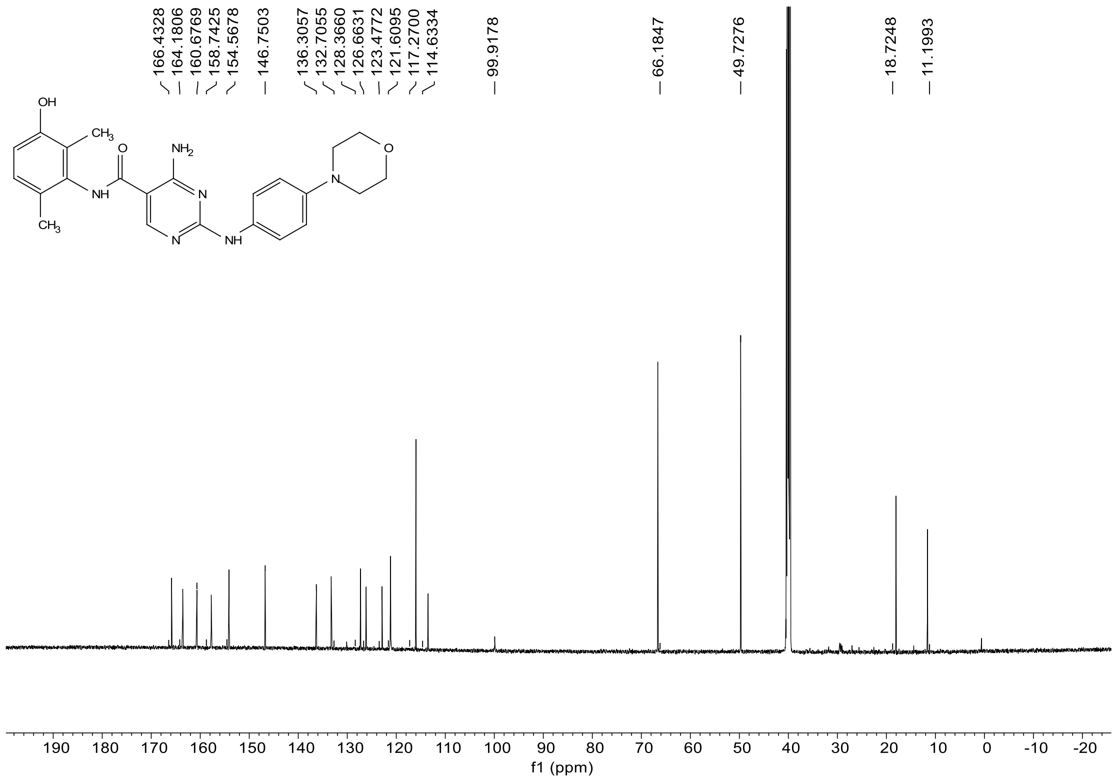

Fragmentor Voltage	Collision Energy	Ionization Mode
200	0	ESI

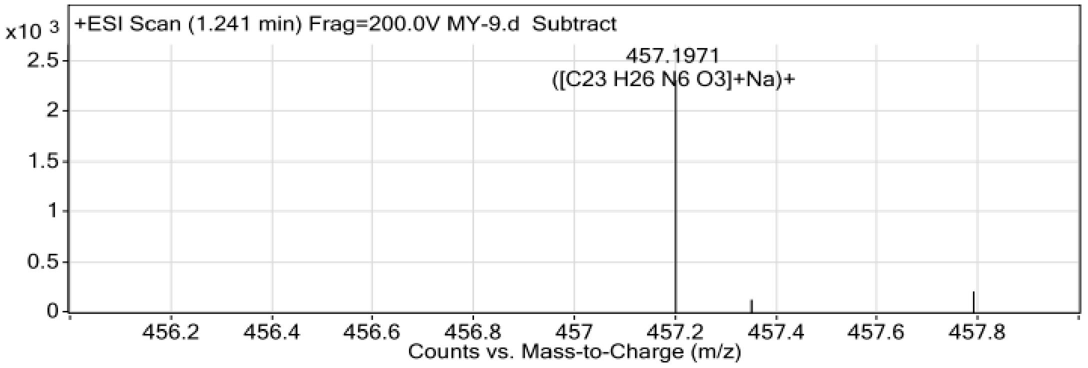

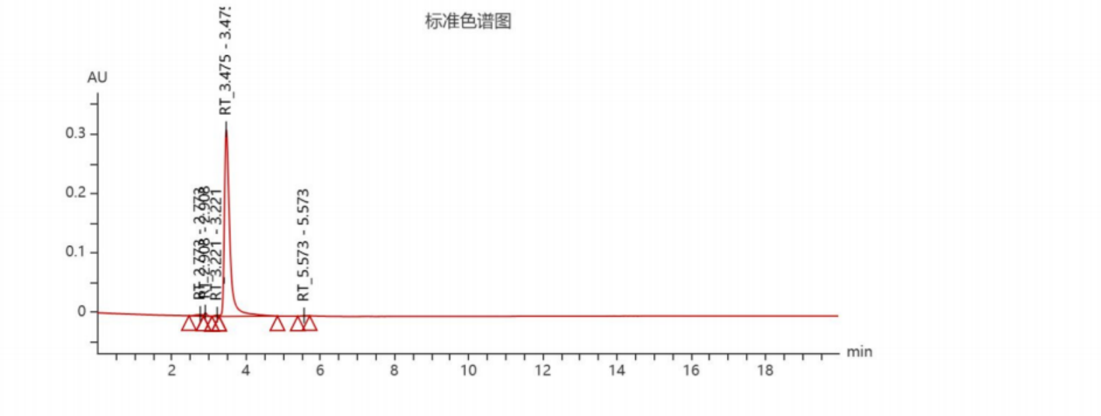

结果表

#	保留时间(min)	峰面积(μV*s)	峰高(μV)	百分面积(%)	百分高度(%)
1	2.773	34341.224	2437.468	1.037	0.750
2	2.908	30564.719	5620.483	0.923	1.730
3	3.221	17091.070	1992.666	0.516	0.614
4	3.475	3225543.864	314427.064	97.409	96.806
5	5.573	3801.300	324.698	0.115	0.100

Figure S17 The ^{1}HNMR, ^{13}CNMR, HRMS and HPLC spectrum of Compound MY-10.

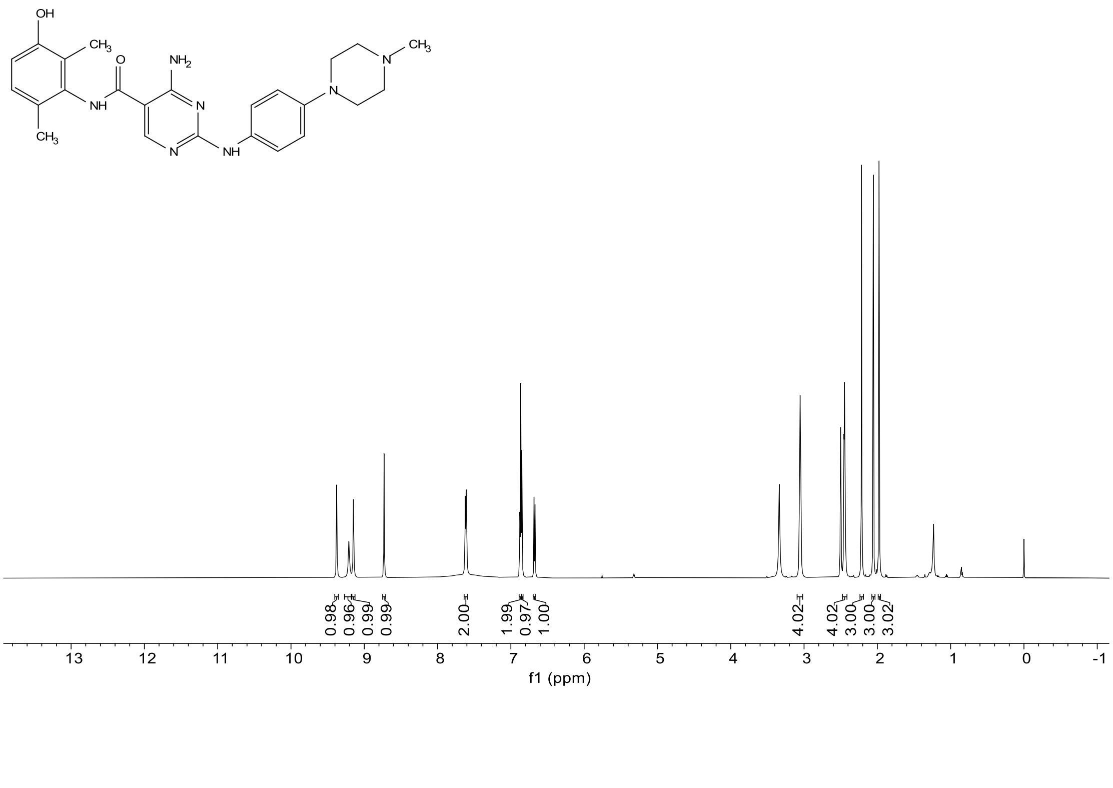

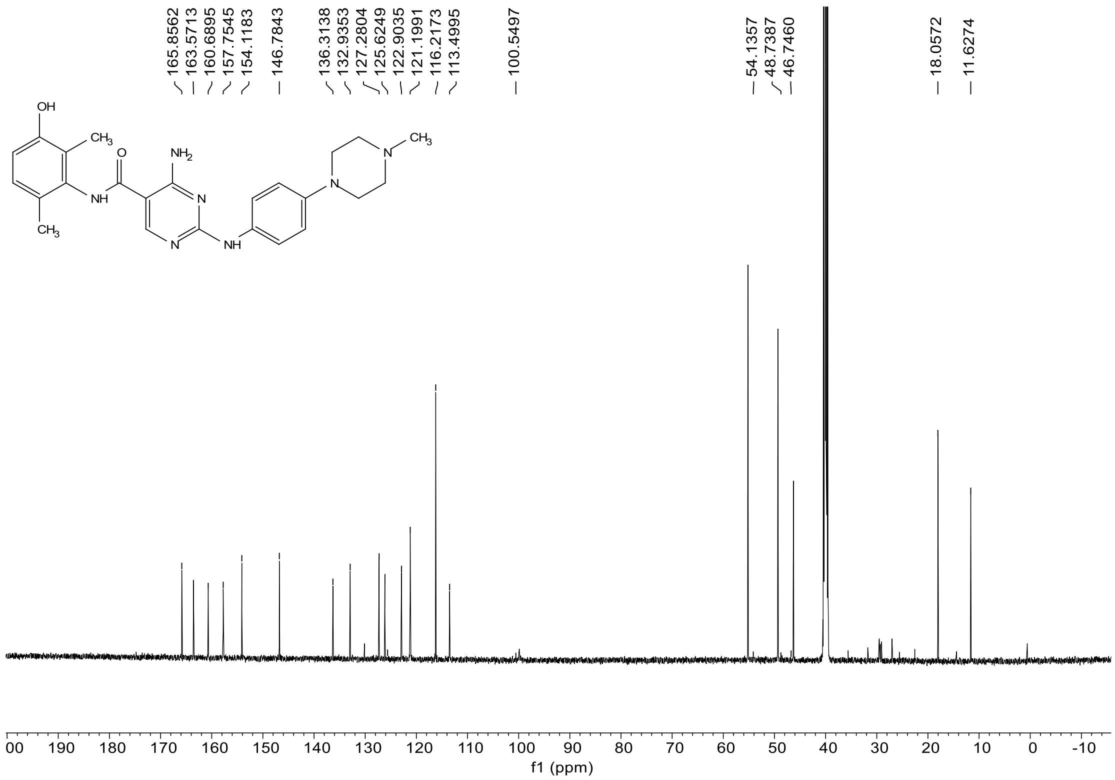
165.8562
163.5713
160.6895
157.7545
154.1183
146.7843
136.3138
132.9353
127.2804
125.6249
122.9035
121.1991
116.2173
113.4995
100.5497
54.1357
48.7387
46.7460
18.0572
11.6274
OH
CH3
O
NH2
CH3
NH
N
N
N
N
NH
CH3
00 190 180 170 160 150 140 130 120 110 100 90 80 70 60 50 40 30 20 10 0 -10
f1 (ppm)

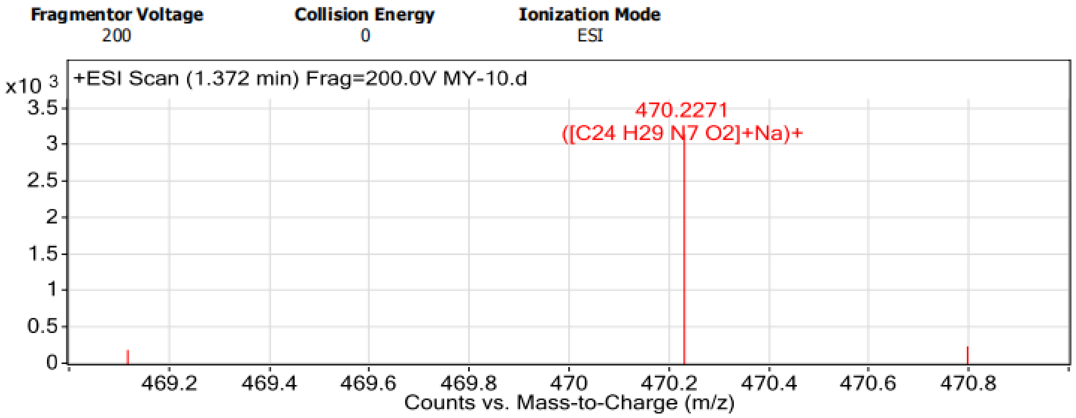
Fragmentor Voltage
200
Collision Energy
0
Ionization Mode
ESI
x10 3
+ESI Scan (1.372 min) Frag=200.0V MY-10.d
470.2271
([C24 H29 N7 O2]+Na)+
3.5
3
2.5
2
1.5
1
0.5
0
469.2 469.4 469.6 469.8 470 470.2 470.4 470.6 470.8
Counts vs. Mass-to-Charge (m/z)

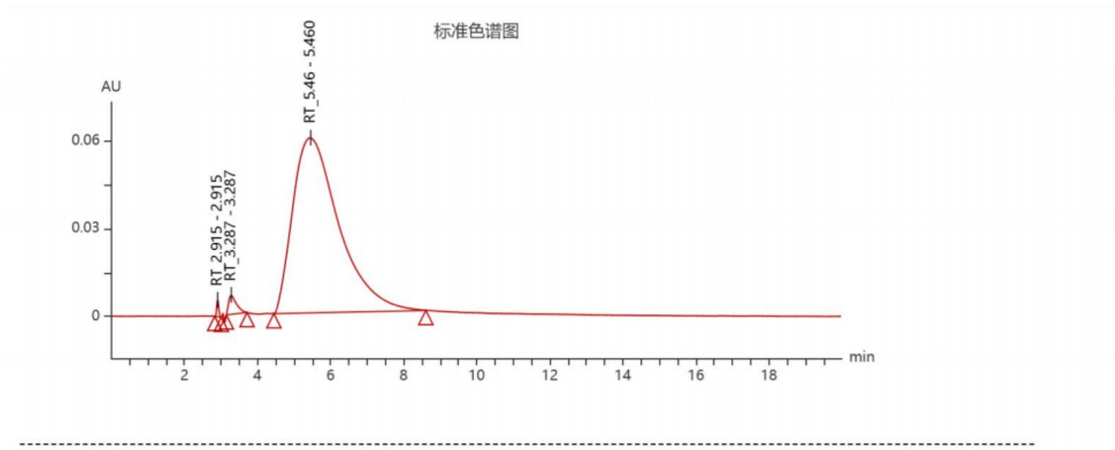

结果表

#	保留时间(min)	峰面积(μV*s)	峰高(μV)	百分面积(%)	百分高度(%)
1	2.915	21984.681	5595.657	0.409	7.787
2	3.287	97459.650	6499.544	1.813	9.045
3	5.460	5256790.874	59760.468	97.778	83.167

Figure S18 The ^{1}HNMR, ^{13}CNMR, HRMS and HPLC spectrum of Compound MY-11.

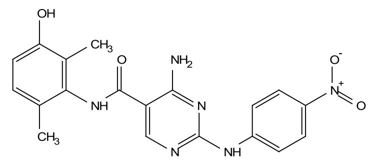

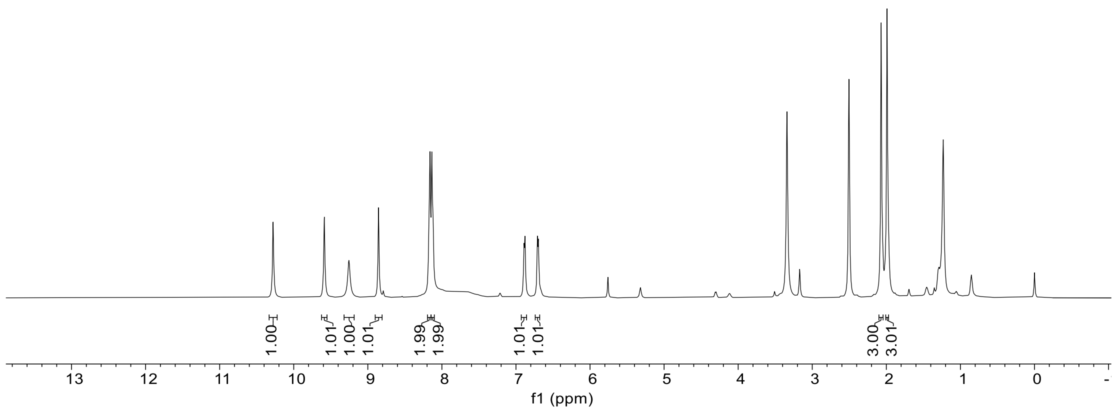

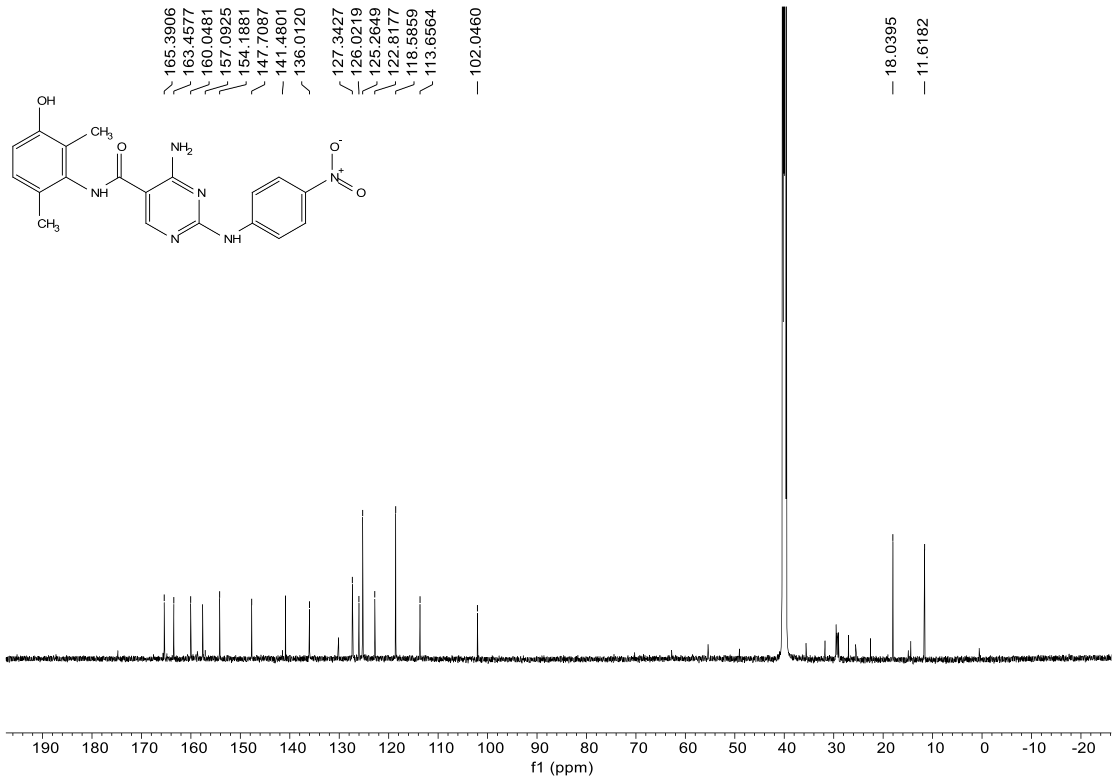

Fragmentor Voltage	Collision Energy	Ionization Mode
200	0	ESI

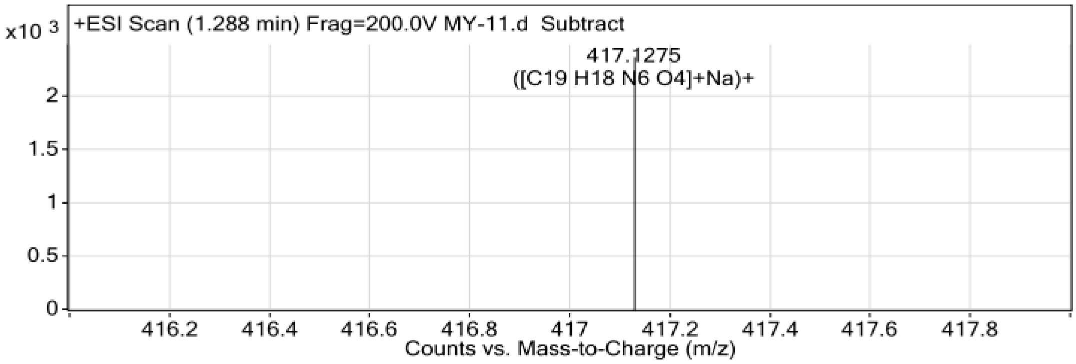

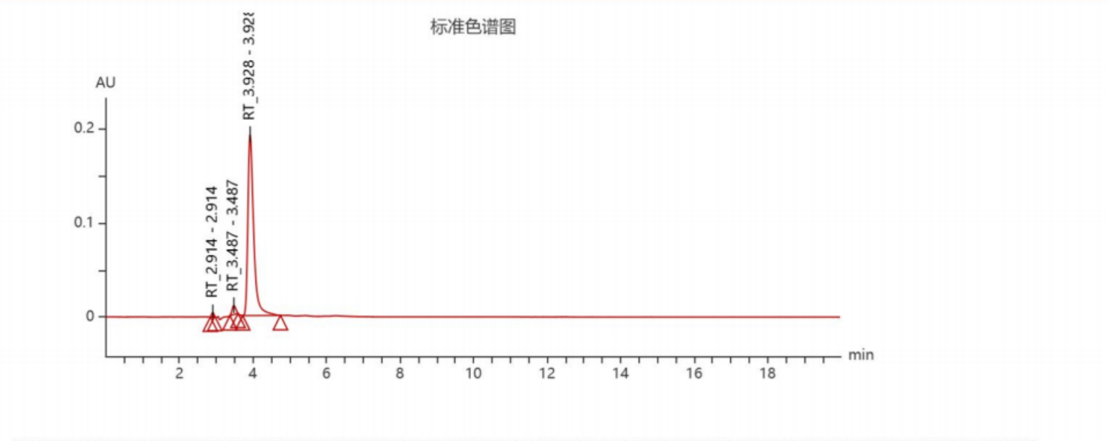

结果表

#	保留时间(min)	峰面积(μV*s)	峰高(μV)	百分面积(%)	百分高度(%)
1	2.914	16841.600	4271.008	0.771	2.069
2	3.487	65827.833	9581.121	3.013	4.642
3	3.928	2102418.967	192539.027	96.217	93.288

Figure S19 The ^{1}HNMR, ^{13}CNMR, HRMS and HPLC spectrum of Compound MY-12.

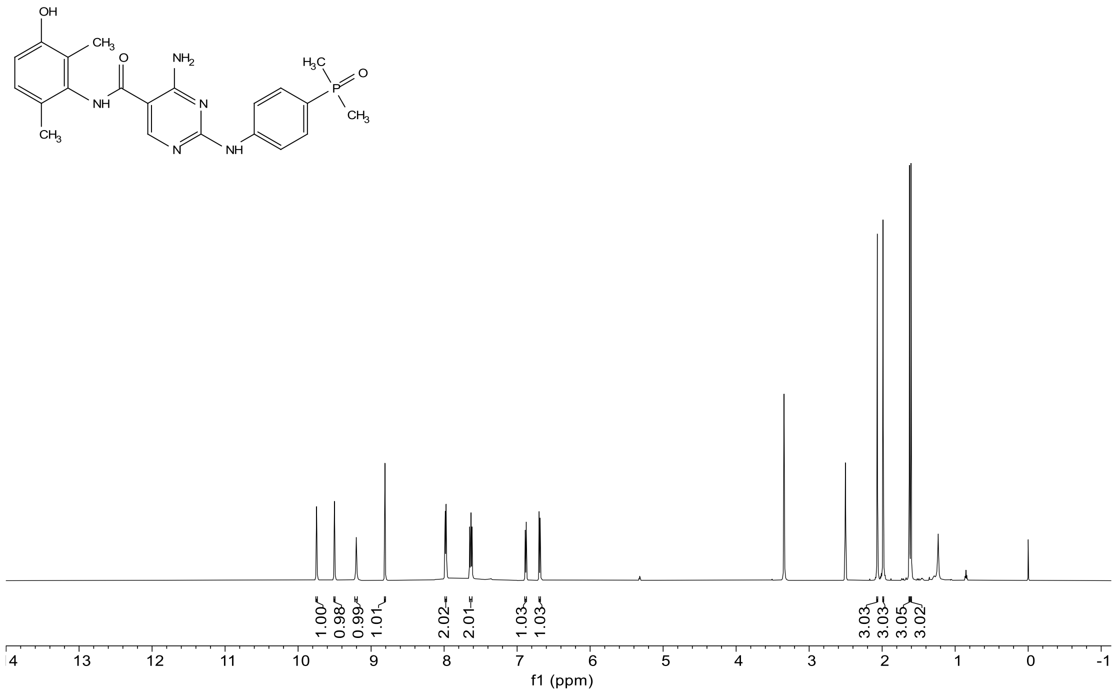

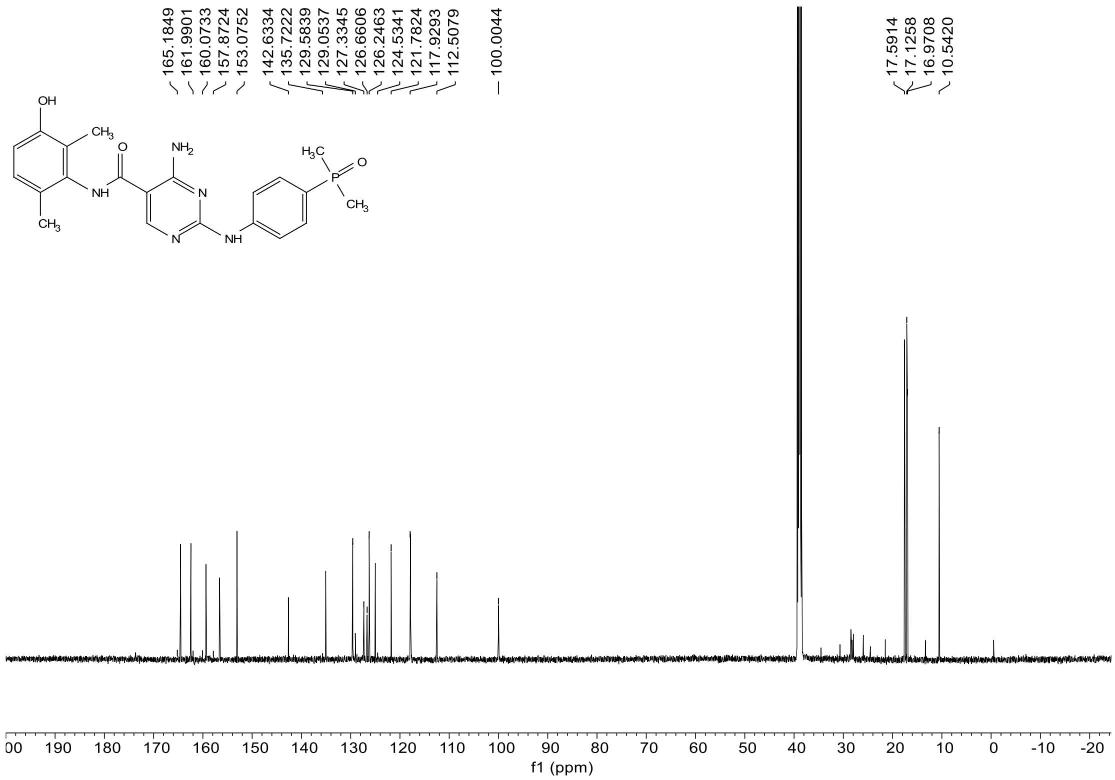

Fragmentor Voltage	Collision Energy	Ionization Mode
200	0	ESI

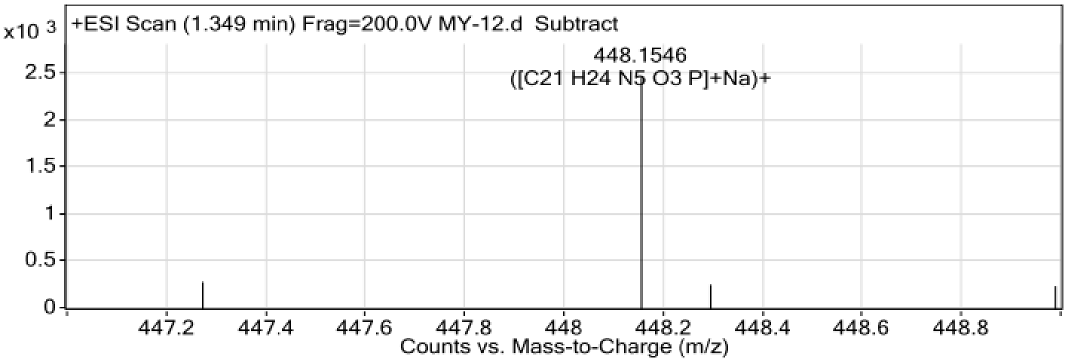

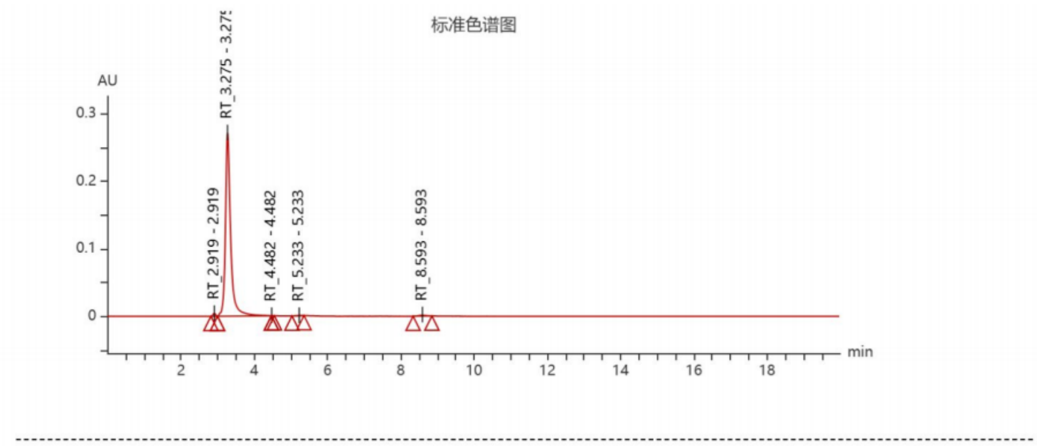

结果表

#	保留时间(min)	峰面积(μV*s)	峰高(μV)	百分面积(%)	百分高度(%)
1	2.919	20441.645	3872.287	0.742	1.396
2	3.275	2713210.133	271862.053	98.481	98.010
3	4.482	365.205	130.319	0.013	0.047
4	5.233	2918.208	281.500	0.106	0.101
5	8.593	18111.158	1236.419	0.657	0.446

Figure S20 The ^{1}HNMR, ^{13}CNMR, HRMS and HPLC spectrum of Compound MY-13.

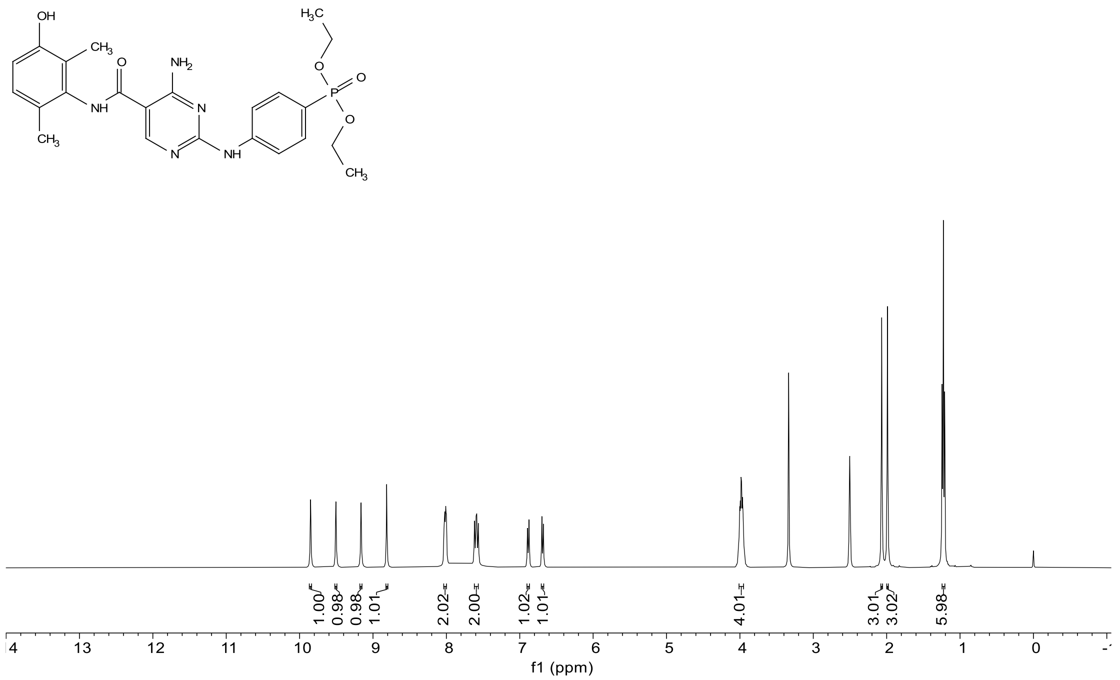

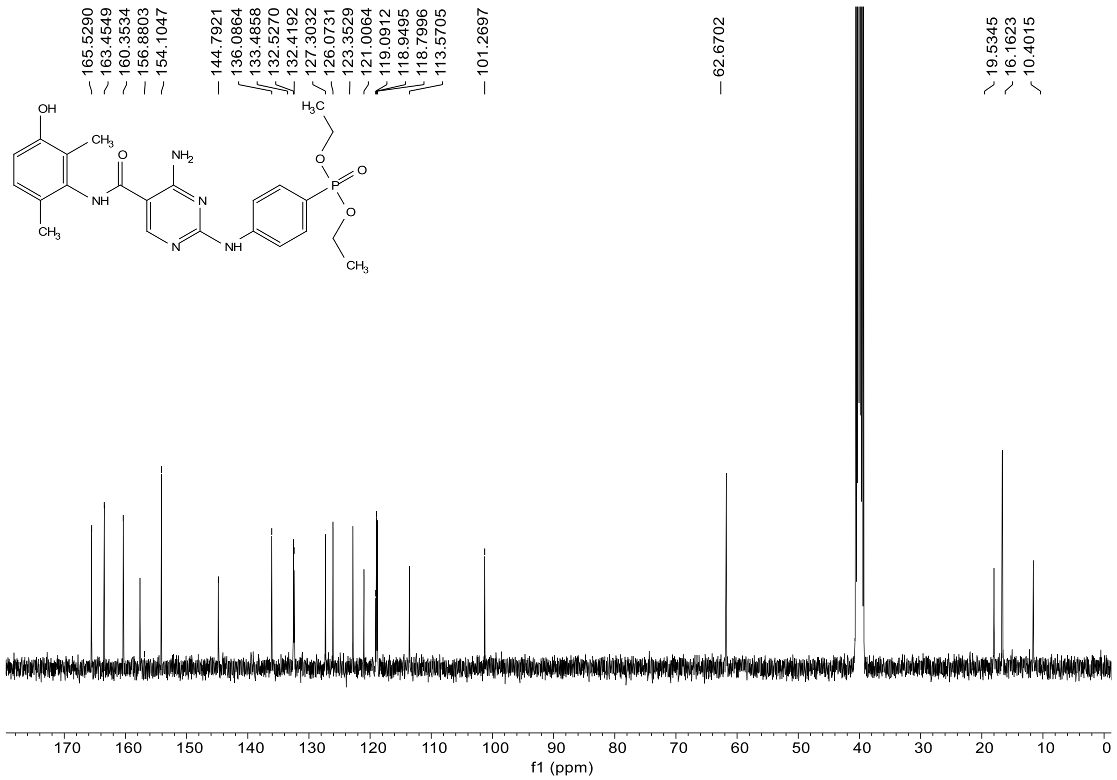
165.5290
163.4549
160.3534
156.8803
154.1047
144.7921
136.0864
133.4858
132.5270
132.4192
127.3032
126.0731
123.3529
121.0064
119.0912
118.9495
118.7996
113.5705
101.2697
62.6702
19.5345
16.1623
10.4015
OH
CH3
O
NH2
NH
N
CH3
N
NH
H3C
O
P
O
O
CH3
170 160 150 140 130 120 110 100 90 80 70 60 50 40 30 20 10 0
f1 (ppm)

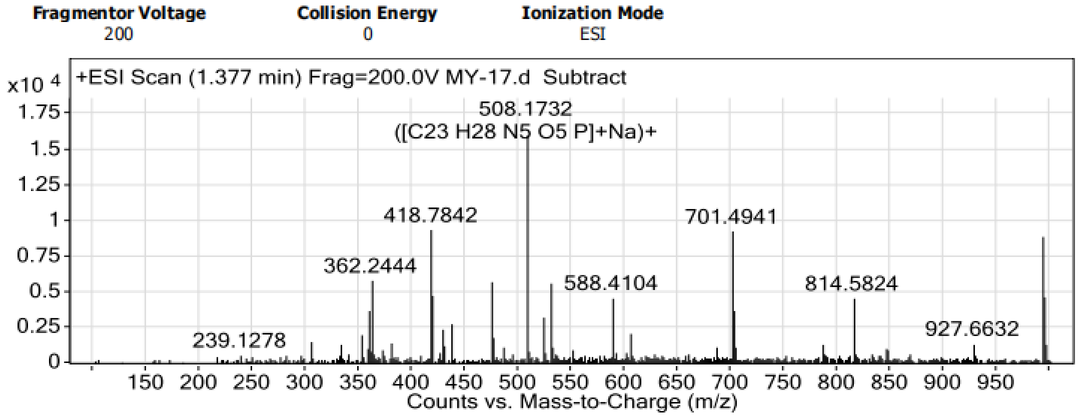
Fragmentor Voltage
200
Collision Energy
0
Ionization Mode
ESI
x10 4
+ESI Scan (1.377 min) Frag=200.0V MY-17.d Subtract
508.1732
([C23 H28 N5 O5 P]+Na)+
418.7842
701.4941
362.2444
588.4104
814.5824
927.6632
239.1278
1.75
1.5
1.25
1
0.75
0.5
0.25
0
150 200 250 300 350 400 450 500 550 600 650 700 750 800 850 900 950
Counts vs. Mass-to-Charge (m/z)

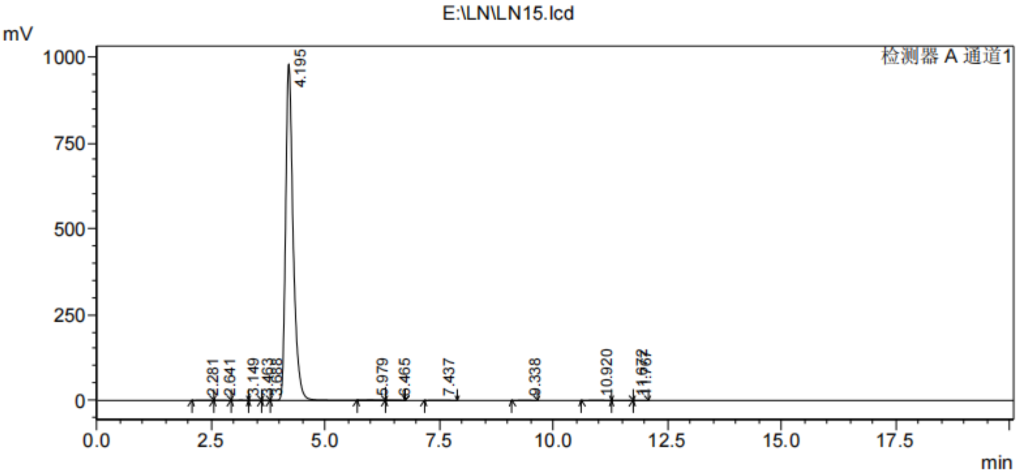

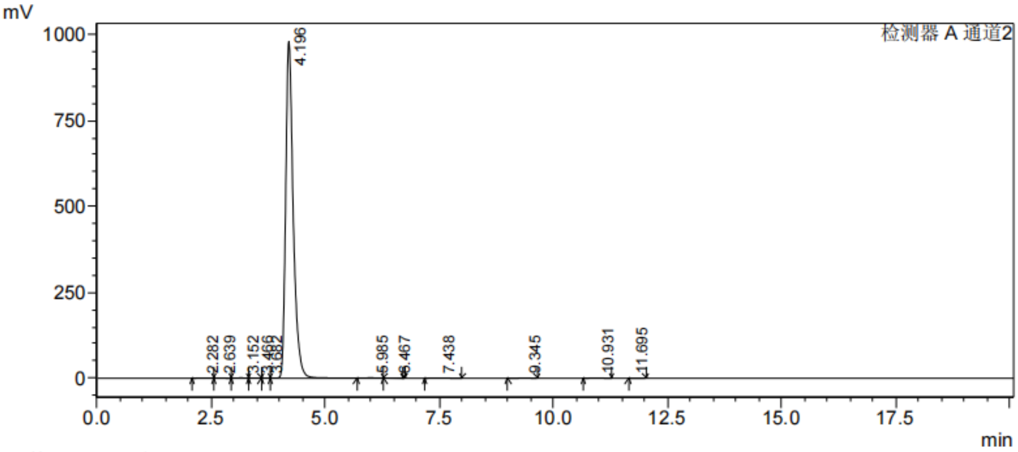

1 检测器 A 通道1/254nm
2 检测器 A 通道2/254nm

峰#	保留时间	面积	高度	面积 %	高度 %
1	2.281	9877	635	0.087	0.064
2	2.641	4451	269	0.039	0.027
3	3.149	13389	1025	0.118	0.104
4	3.463	9092	812	0.080	0.082
5	3.688	6447	595	0.057	0.060
6	4.195	11277454	979347	99.295	99.427
7	5.979	13061	812	0.115	0.082
8	6.465	3973	332	0.035	0.034
9	7.437	7590	427	0.067	0.043
10	9.338	4005	215	0.035	0.022
11	10.920	5364	306	0.047	0.031
12	11.672	1792	116	0.016	0.012
13	11.767	1015	95	0.009	0.010
总计		11357511	984987	100.000	100.000

Figure S21 The ^{1}HNMR, ^{13}CNMR, HRMS and HPLC spectrum of Compound MY-14.

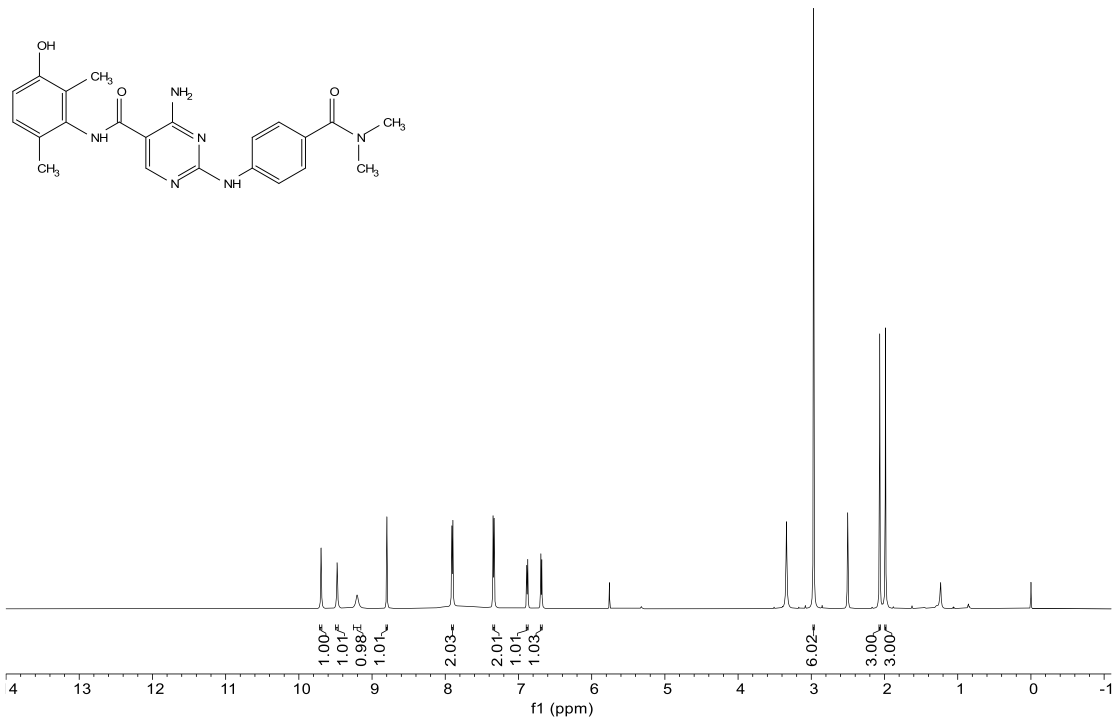
OH
CH3
O
NH2
NH
N
CH3
N
CH3
N
NH
CH3
1.00
1.01
0.98
1.01
2.03
2.01
1.01
1.03
6.02
3.00
3.00
f1 (ppm)

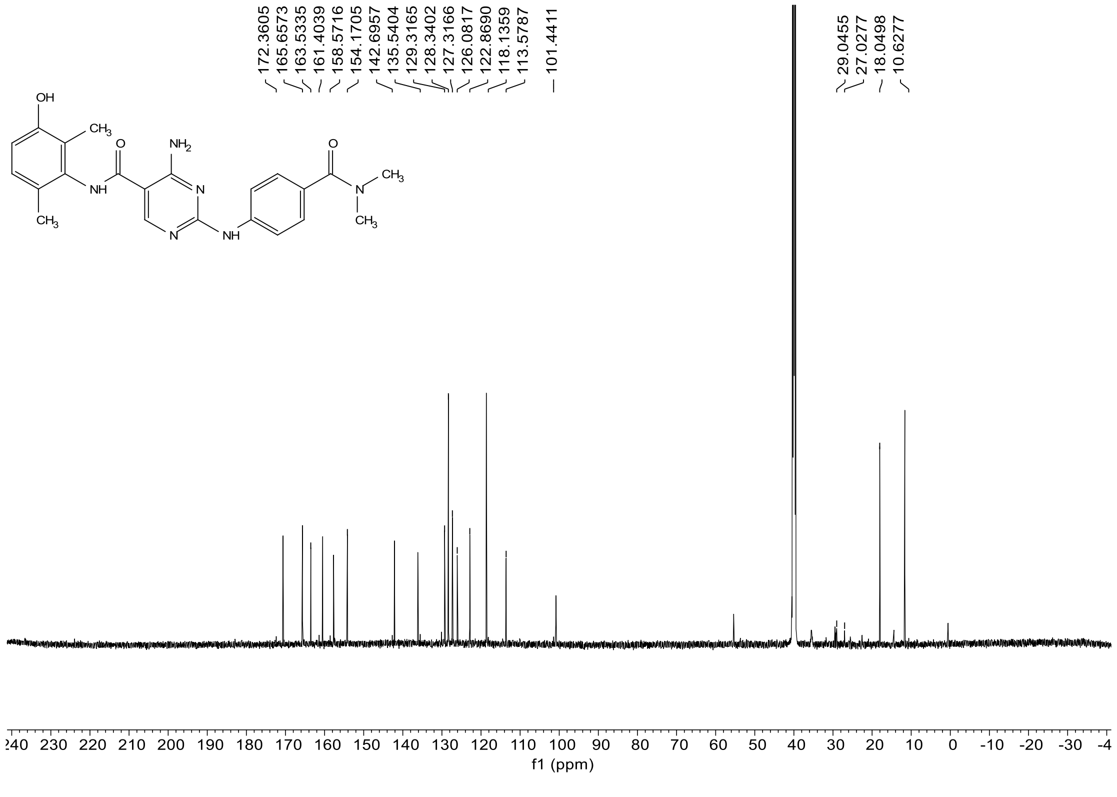
172.3605
165.6573
163.5335
161.4039
158.5716
154.1705
142.6957
135.5404
129.3165
128.3402
127.3166
126.0817
122.8690
118.1359
113.5787
101.4411
29.0455
27.0277
18.0498
10.6277
OH
CH3
O
NH2
NH
N
CH3
N
CH3
N
NH
CH3
f1 (ppm)

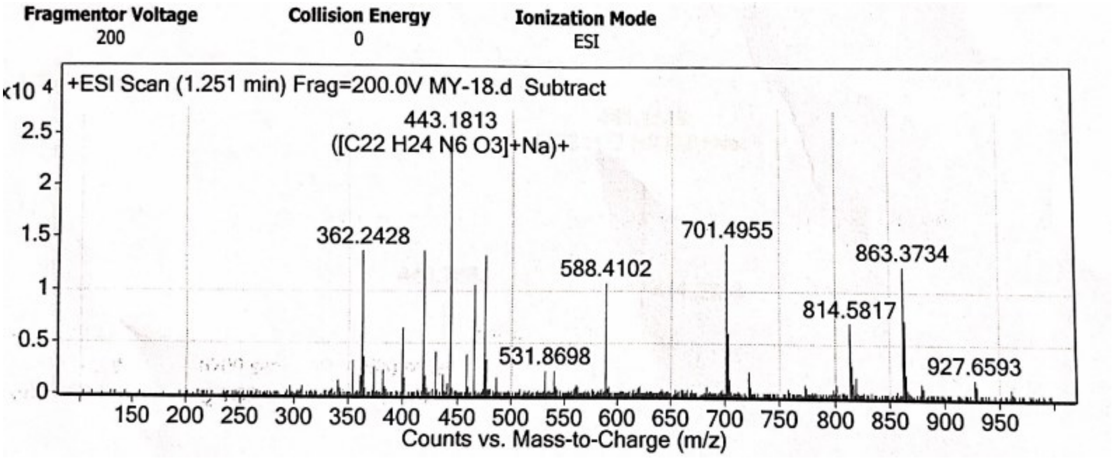

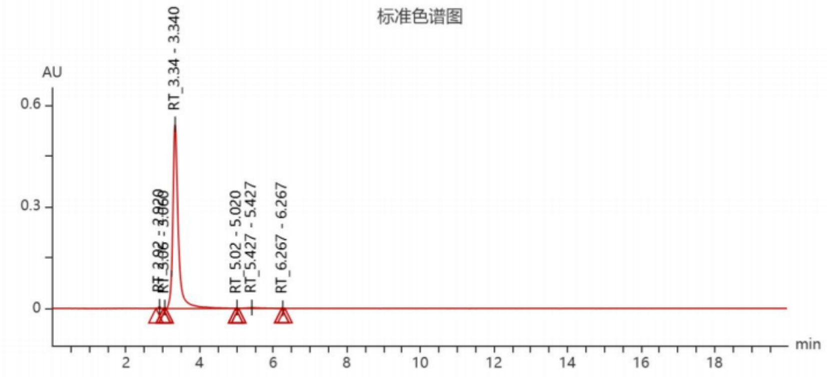

结果表

#	保留时间(min)	峰面积(μV*s)	峰高(μV)	百分面积(%)	百分高度(%)
1	2.920	22079.800	4599.250	0.381	0.834
2	3.060	3188.150	1589.136	0.055	0.288
3	3.340	5679516.056	542051.936	98.055	98.277
4	5.020	4128.195	917.206	0.071	0.166
5	5.427	82949.264	2260.418	1.432	0.410
6	6.267	325.985	135.178	0.006	0.025

Figure S22 The ^{1}HNMR, ^{13}CNMR, HRMS and HPLC spectrum of Compound MY-15.

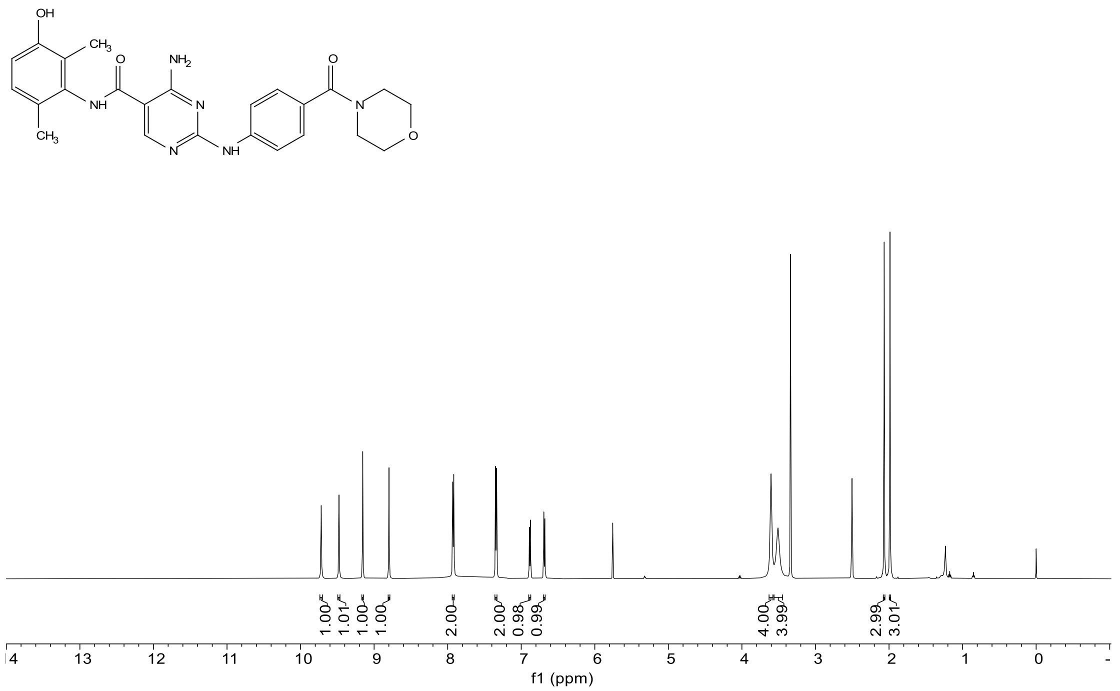
OH
CH3
O
NH2
NH
N
CH3
N
NH
O
N
O
1.00
1.01
1.00
1.00
2.00
2.00
0.98
0.99
4.00
3.99
2.99
3.01
14
13
12
11
10
9
8
7
6
5
4
3
2
1
0
f1 (ppm)

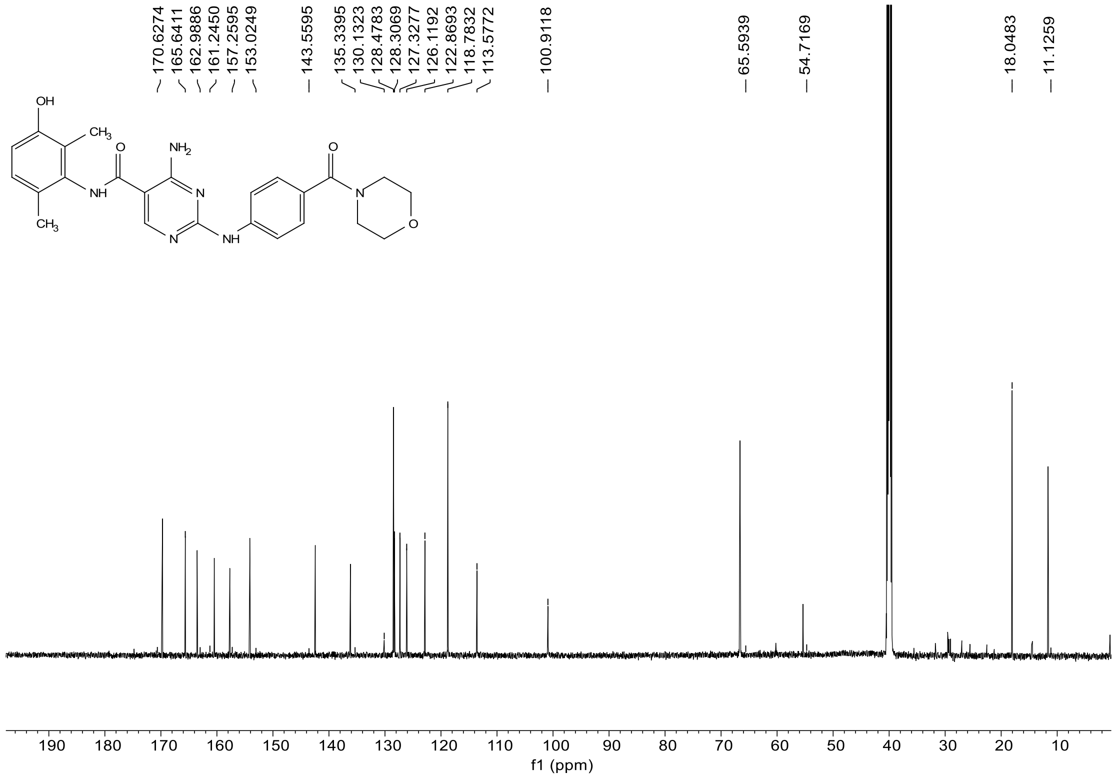
170.6274
165.6411
162.9886
161.2450
157.2595
153.0249
143.5595
135.3395
130.1323
128.4783
128.3069
127.3277
126.1192
122.8693
118.7832
113.5772
100.9118
65.5939
54.7169
18.0483
11.1259
OH
CH3
O
NH2
NH
N
CH3
N
NH
O
N
O
190
180
170
160
150
140
130
120
110
100
90
80
70
60
50
40
30
20
10
f1 (ppm)

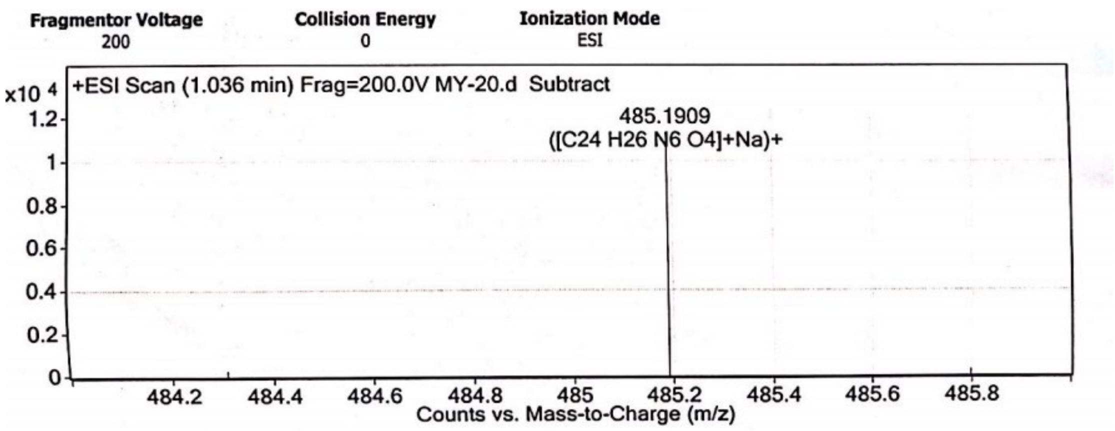

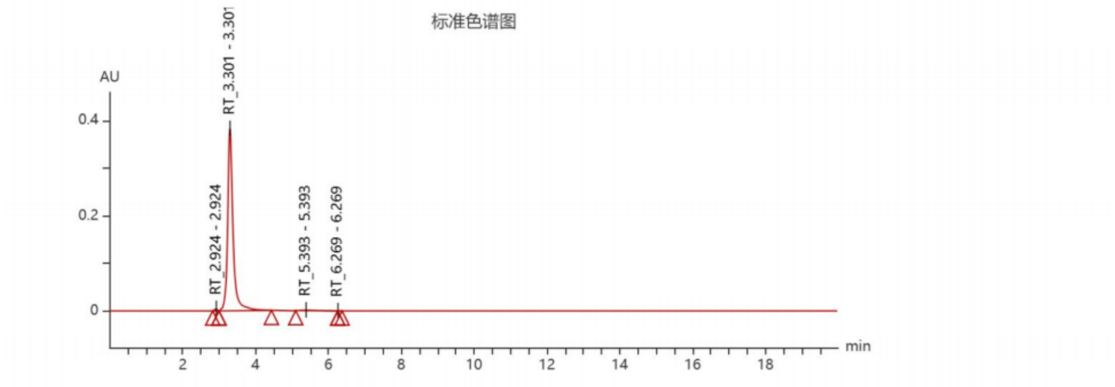

结果表

#	保留时间(min)	峰面积(μV*s)	峰高(μV)	百分面积(%)	百分高度(%)
1	2.924	20005.333	3747.591	0.501	0.967
2	3.301	3934982.433	382420.096	98.595	98.705
3	5.393	35765.133	1181.594	0.896	0.305
4	6.269	292.933	88.353	0.007	0.023

Figure S23 The ^{1}HNMR, ^{13}CNMR, HRMS and HPLC spectrum of Compound MY-16.

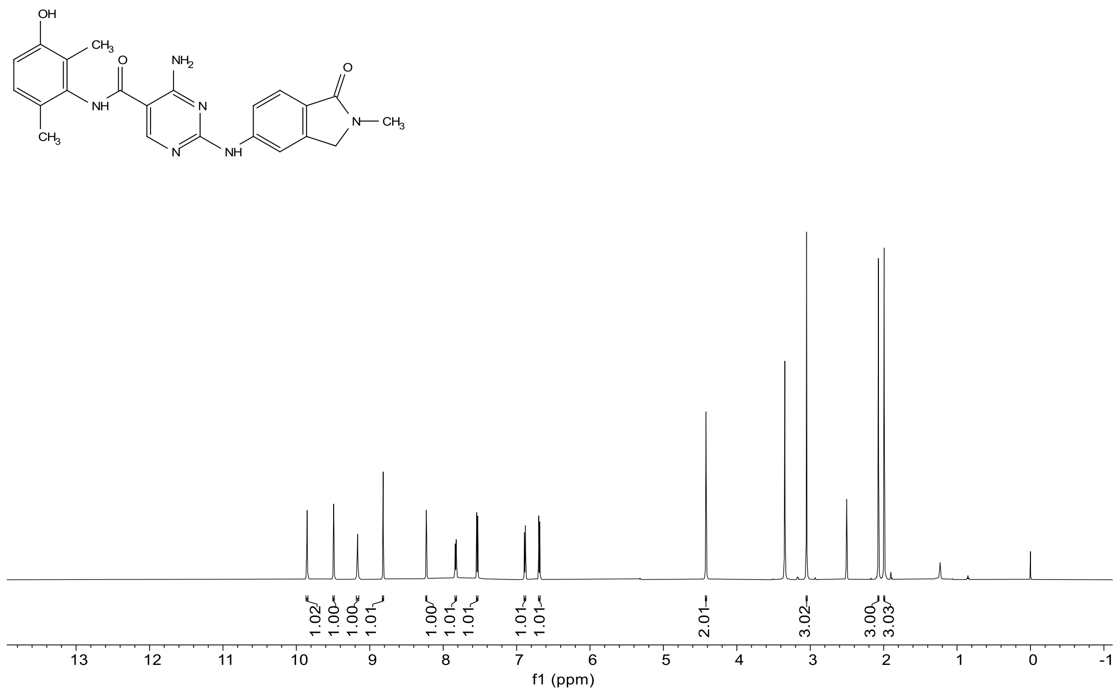

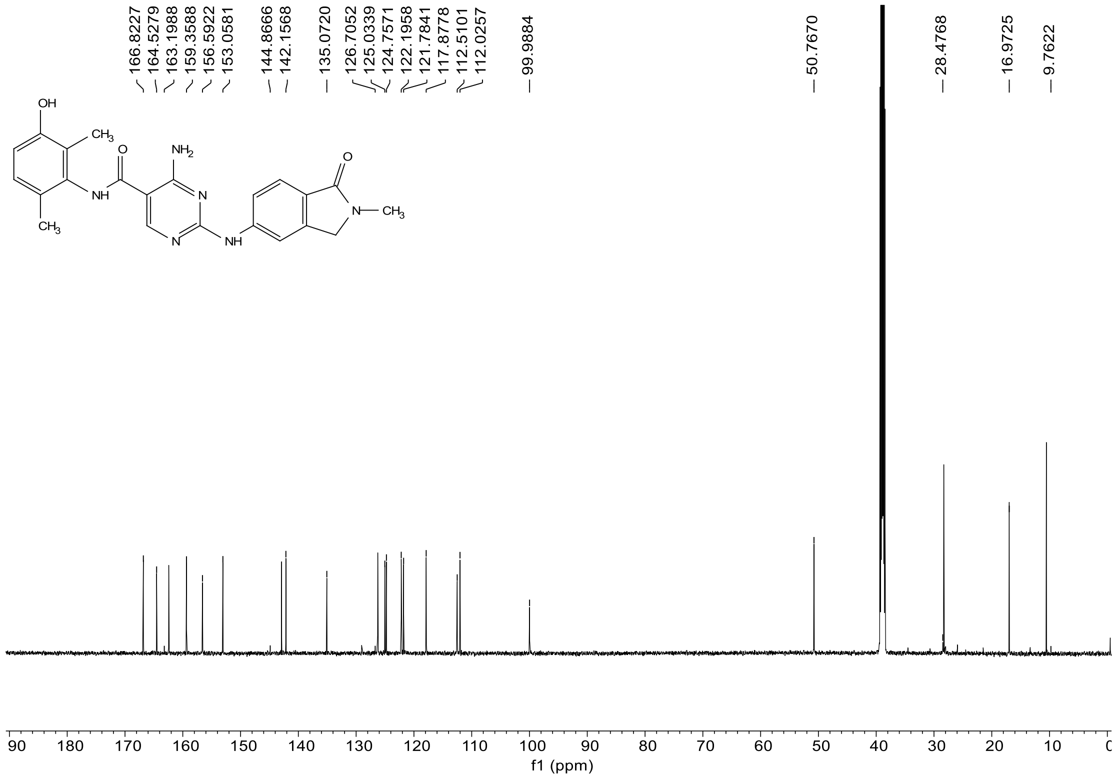

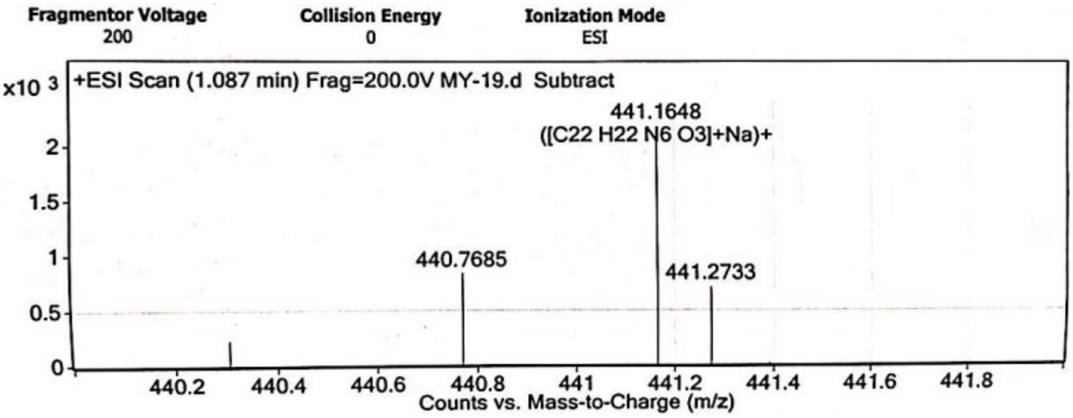

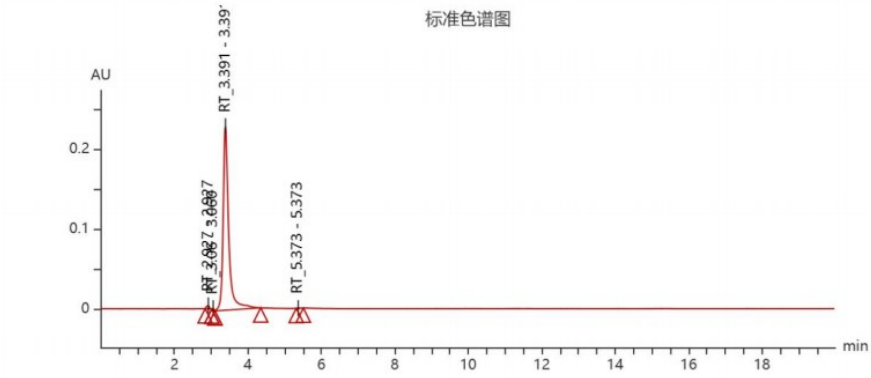

结果表

#	保留时间(min)	峰面积(μV*s)	峰高(μV)	百分面积(%)	百分高度(%)
1	2.927	21807.558	3894.815	0.891	1.650
2	3.060	4765.725	2072.115	0.195	0.878
3	3.391	2419760.000	229887.768	98.880	97.418
4	5.373	838.100	126.897	0.034	0.054

Figure S24 The ^{1}HNMR, ^{13}CNMR, HRMS and HPLC spectrum of Compound MY-17.

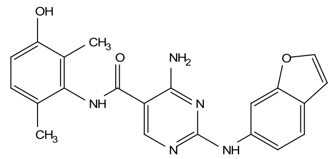

OH
CH3
O
NH2
NH
N
CH3
N
NH
O

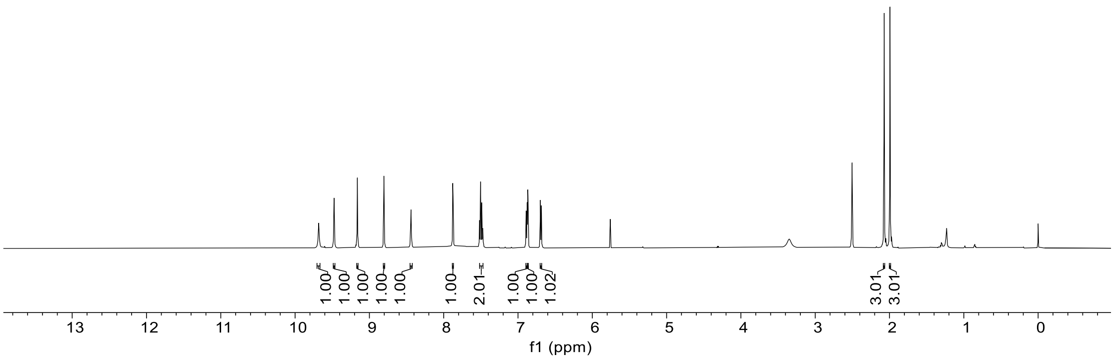

1.00
1.00
1.00
1.00
1.00
1.00
2.01
1.00
1.00
1.02
3.01
3.01
13 12 11 10 9 8 7 6 5 4 3 2 1 0
f1 (ppm)

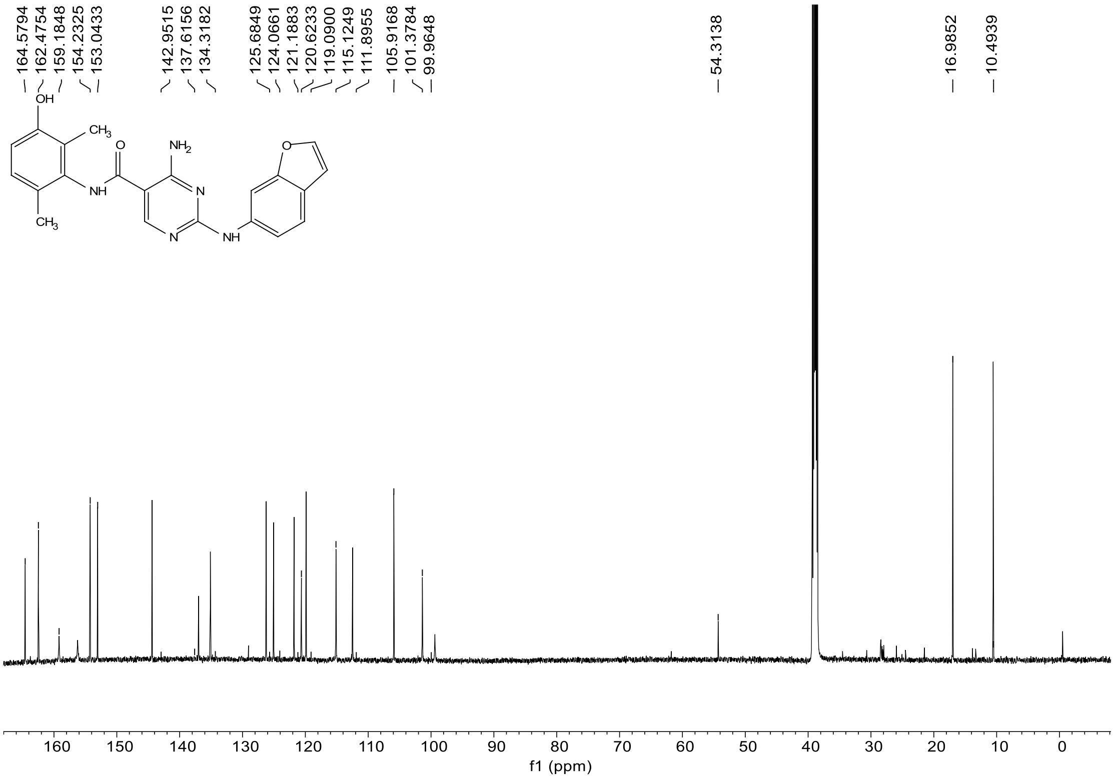

164.5794
162.4754
159.1848
154.2325
153.0433
142.9515
137.6156
134.3182
125.6849
124.0661
121.1883
120.6233
119.0900
115.1249
111.8955
105.9168
101.3784
99.9648
54.3138
16.9852
10.4939
OH
CH3
O
NH2
NH
N
CH3
N
NH
O
160 150 140 130 120 110 100 90 80 70 60 50 40 30 20 10 0
f1 (ppm)

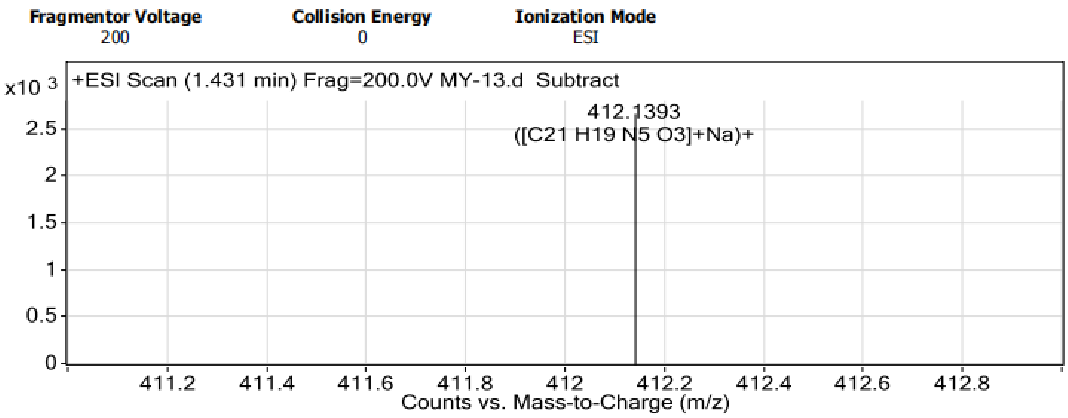

标准色谱图

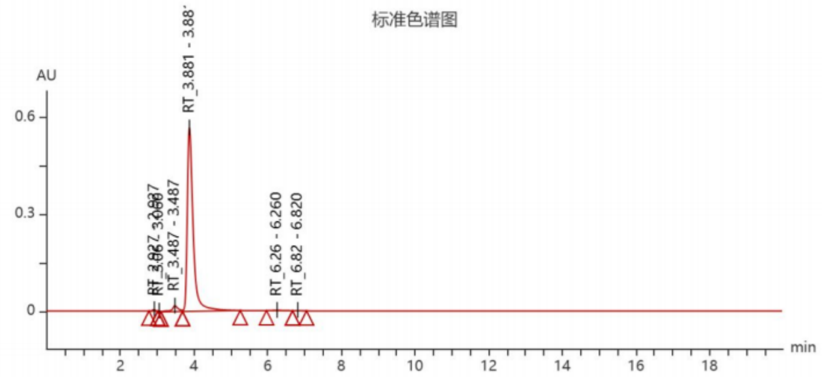

结果表

#	保留时间(min)	峰面积(μV*s)	峰高(μV)	百分面积(%)	百分高度(%)
1	2.927	24207.333	4048.945	0.366	0.681
2	3.060	5122.750	2221.981	0.077	0.374
3	3.487	229323.572	17990.265	3.466	3.026
4	3.881	6318929.687	568404.717	95.509	95.622
5	6.260	29275.103	1086.394	0.442	0.183
6	6.820	9213.547	675.693	0.139	0.114

Figure S25 The ^{1}HNMR, ^{13}CNMR, HRMS and HPLC spectrum of Compound MY-18.

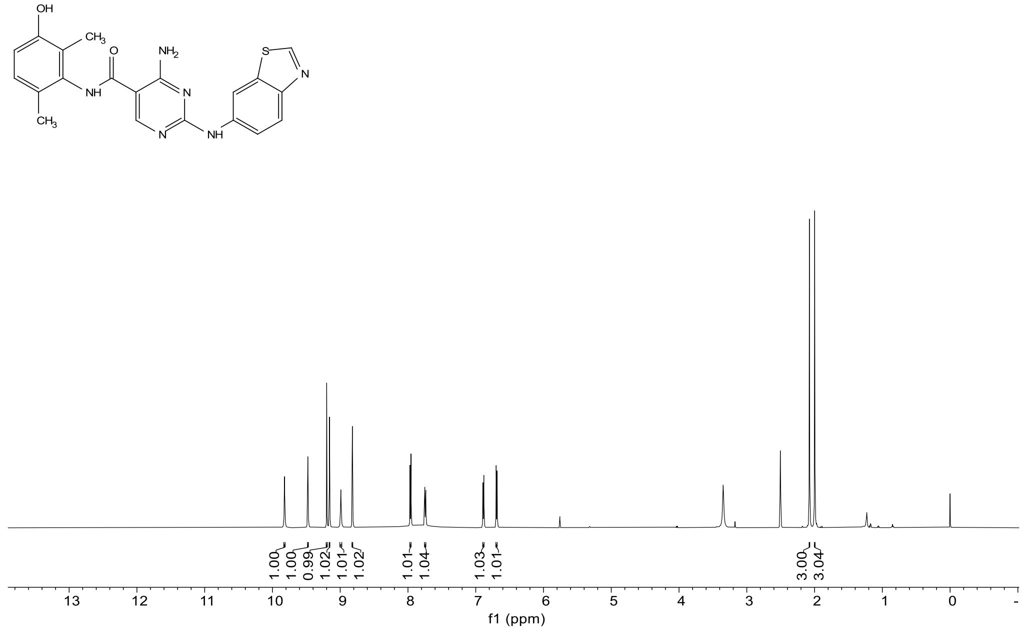

OH
CH3
O
NH2
N
S
N
NH
CH3
N
NH
1.00
1.00
0.99
1.02
1.01
1.02
1.01
1.04
1.03
1.01
3.00
3.04
13 12 11 10 9 8 7 6 5 4 3 2 1 0
f1 (ppm)

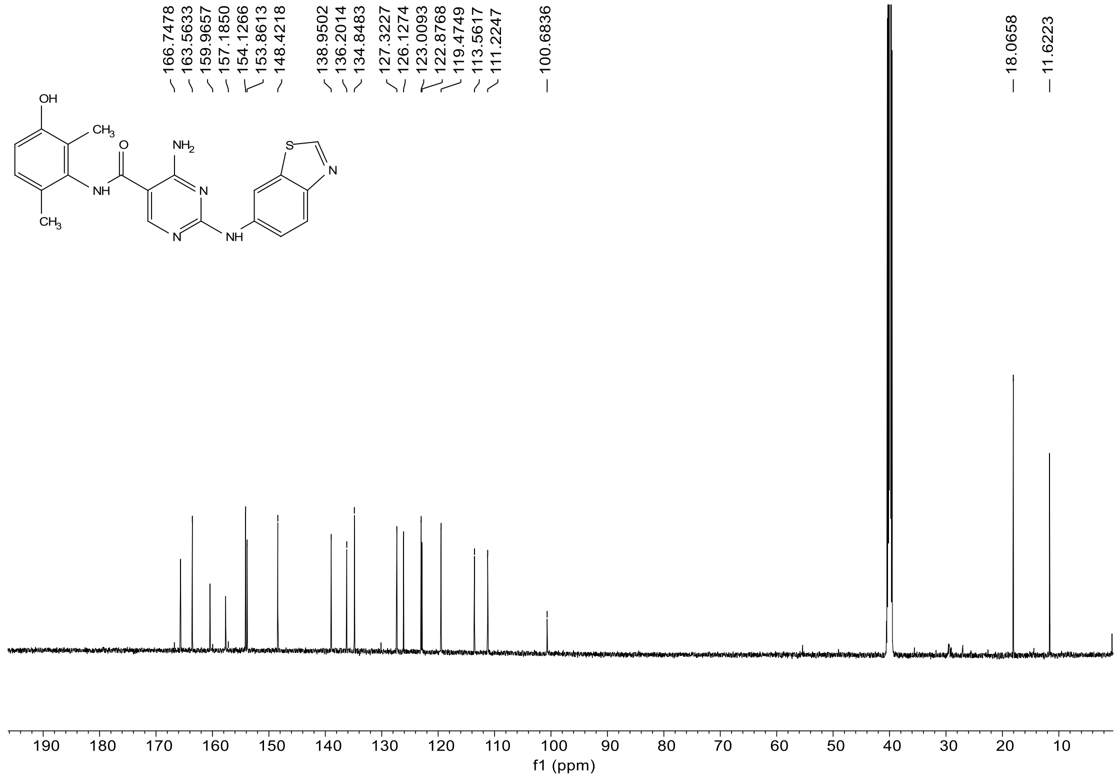

166.7478
163.5633
159.9657
157.1850
154.1266
153.8613
148.4218
138.9502
136.2014
134.8483
127.3227
126.1274
123.0093
122.8768
119.4749
113.5617
111.2247
100.6836
18.0658
11.6223
OH
CH3
O
NH2
N
S
N
NH
CH3
N
NH
190 180 170 160 150 140 130 120 110 100 90 80 70 60 50 40 30 20 10
f1 (ppm)

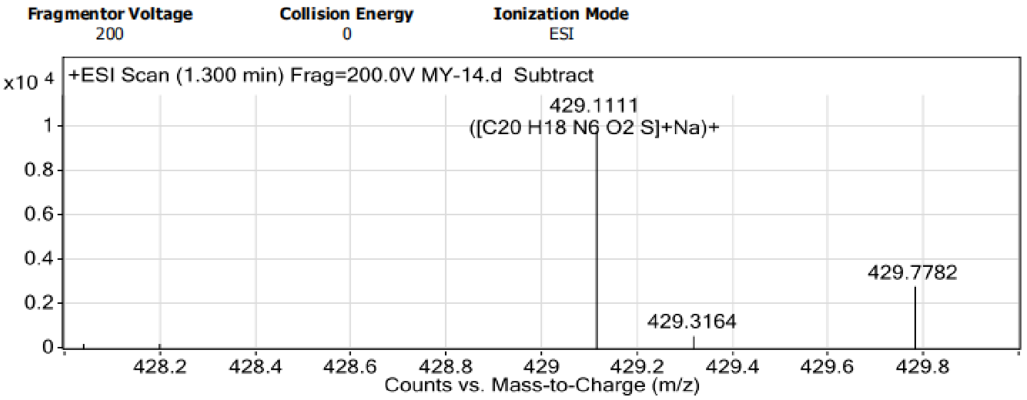

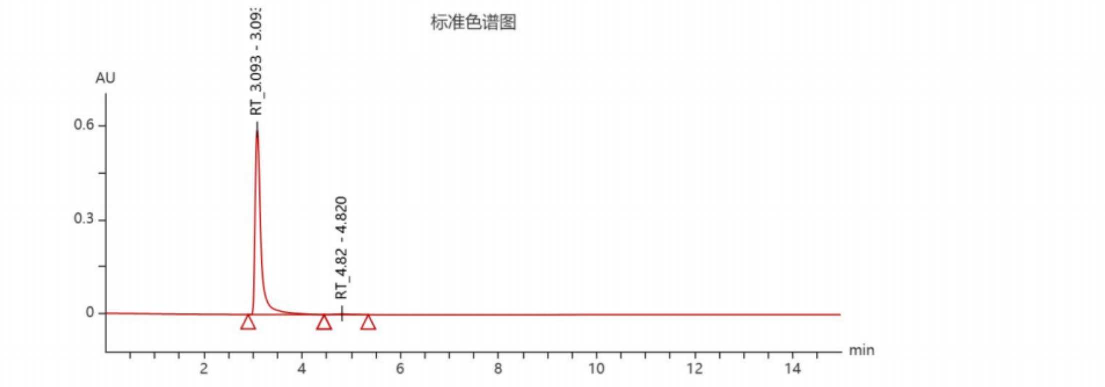

结果表

#	保留时间(min)	峰面积(μV*s)	峰高(μV)	百分面积(%)	百分高度(%)
1	3.093	4985697.138	591979.009	98.832	99.634
2	4.820	58908.971	2175.918	1.168	0.366

Figure S26 The ^{1}HNMR, ^{13}CNMR, HRMS and HPLC spectrum of Compound MY-19.

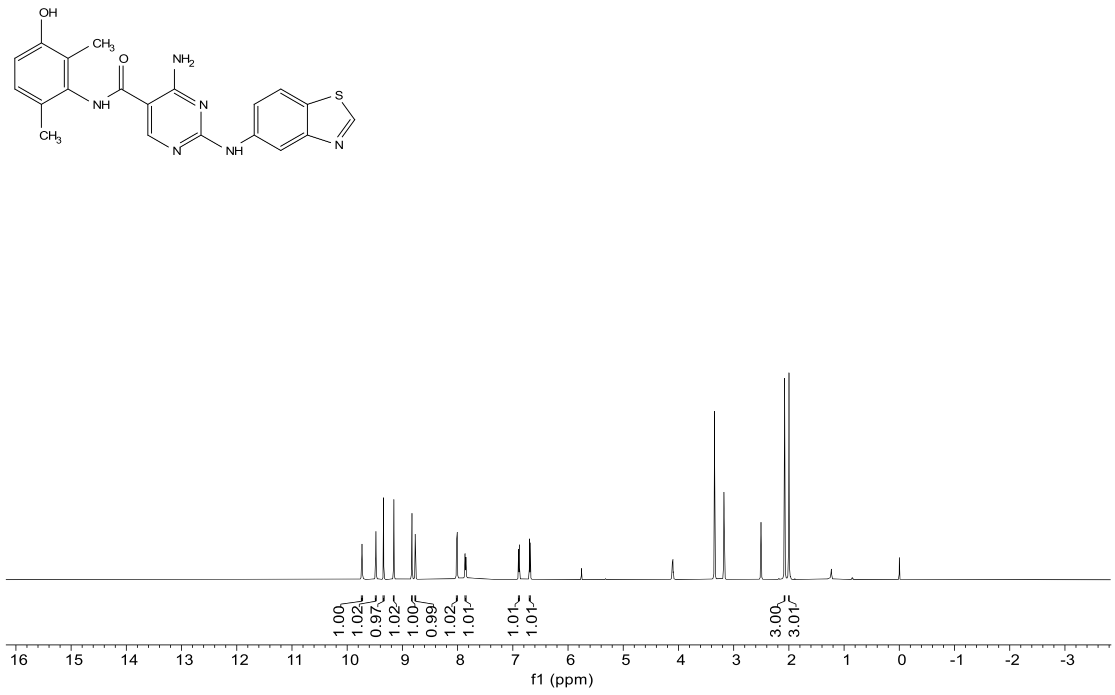

OH
CH3
O
NH2
NH
N
S
CH3
N
NH
N
1.00
1.02
0.97
1.02
1.00
0.99
1.02
1.01
1.01
1.01
3.00
3.01
16 15 14 13 12 11 10 9 8 7 6 5 4 3 2 1 0 -1 -2 -3
f1 (ppm)

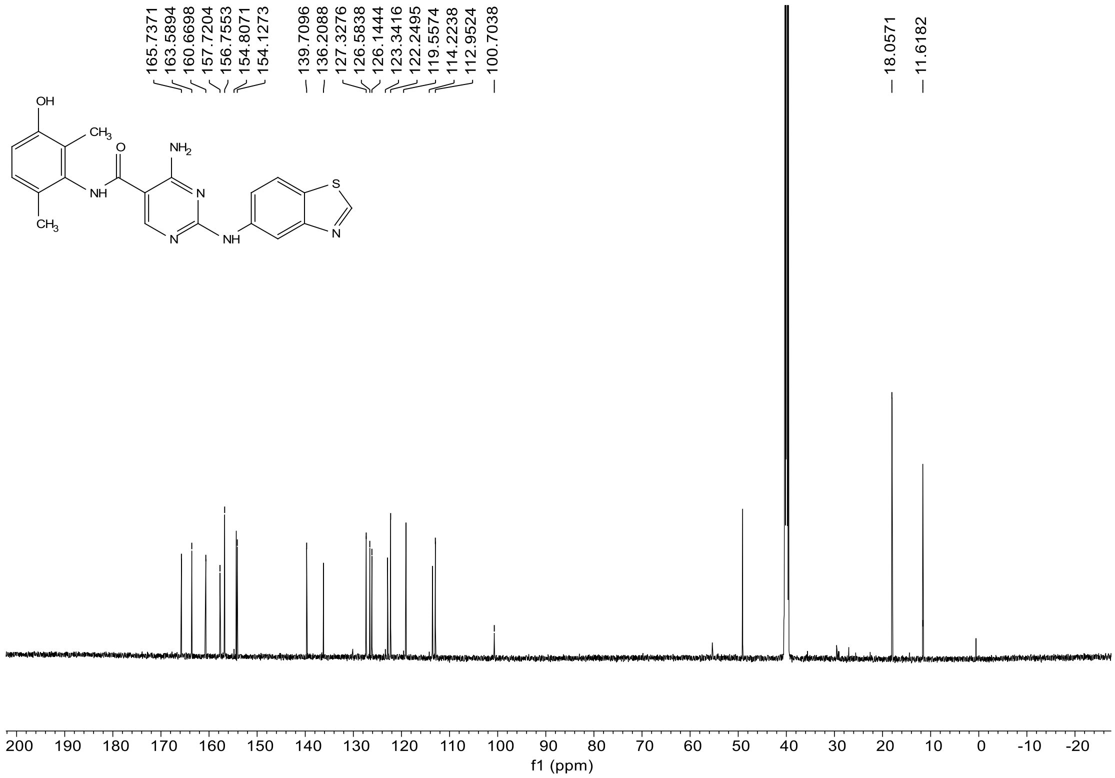

165.7371
163.5894
160.6698
157.7204
156.7553
154.8071
154.1273
139.7096
136.2088
127.3276
126.5838
126.1444
123.3416
122.2495
119.5574
114.2238
112.9524
100.7038
18.0571
11.6182
OH
CH3
O
NH2
NH
N
S
CH3
N
NH
N
200 190 180 170 160 150 140 130 120 110 100 90 80 70 60 50 40 30 20 10 0 -10 -20
f1 (ppm)

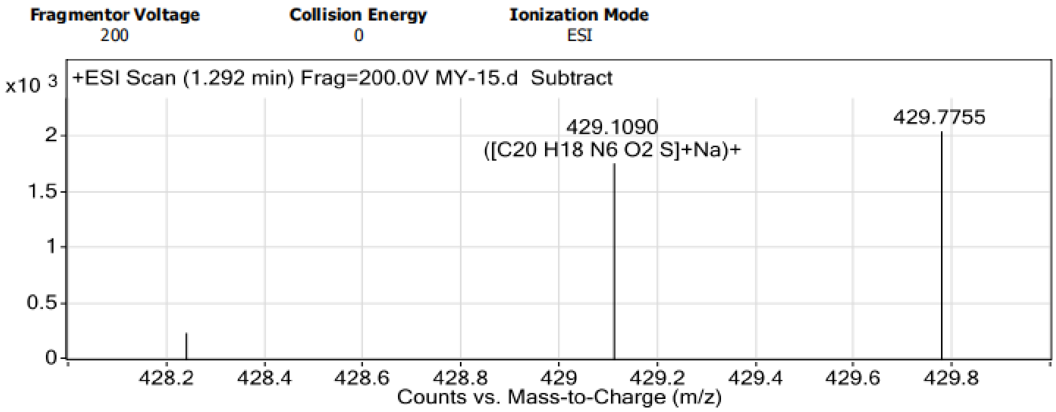

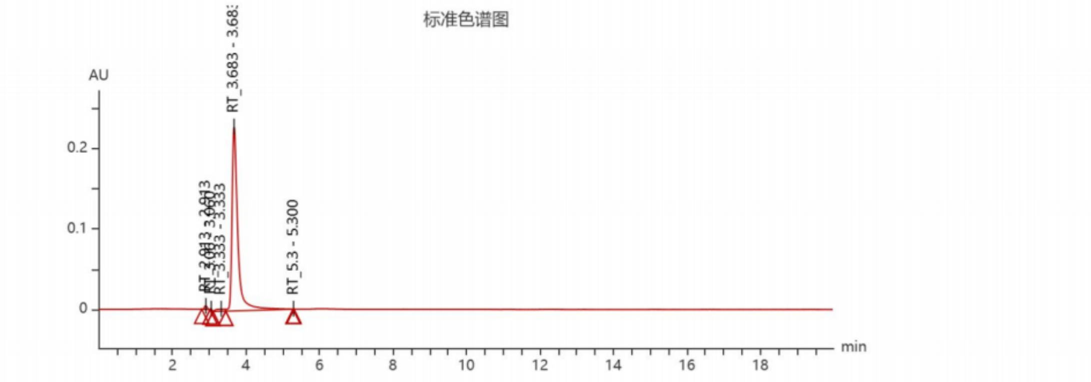

结果表

#	保留时间(min)	峰面积(μV*s)	峰高(μV)	百分面积(%)	百分高度(%)
1	2.913	22641.133	4058.500	0.878	1.708
2	3.060	4513.744	2074.771	0.175	0.873
3	3.333	45862.320	2653.829	1.777	1.117
4	3.683	2507004.962	228689.515	97.164	96.247
5	5.300	146.118	129.407	0.006	0.054

Figure S27 The ^{1}HNMR, ^{13}CNMR, HRMS and HPLC spectrum of Compound MY-20.

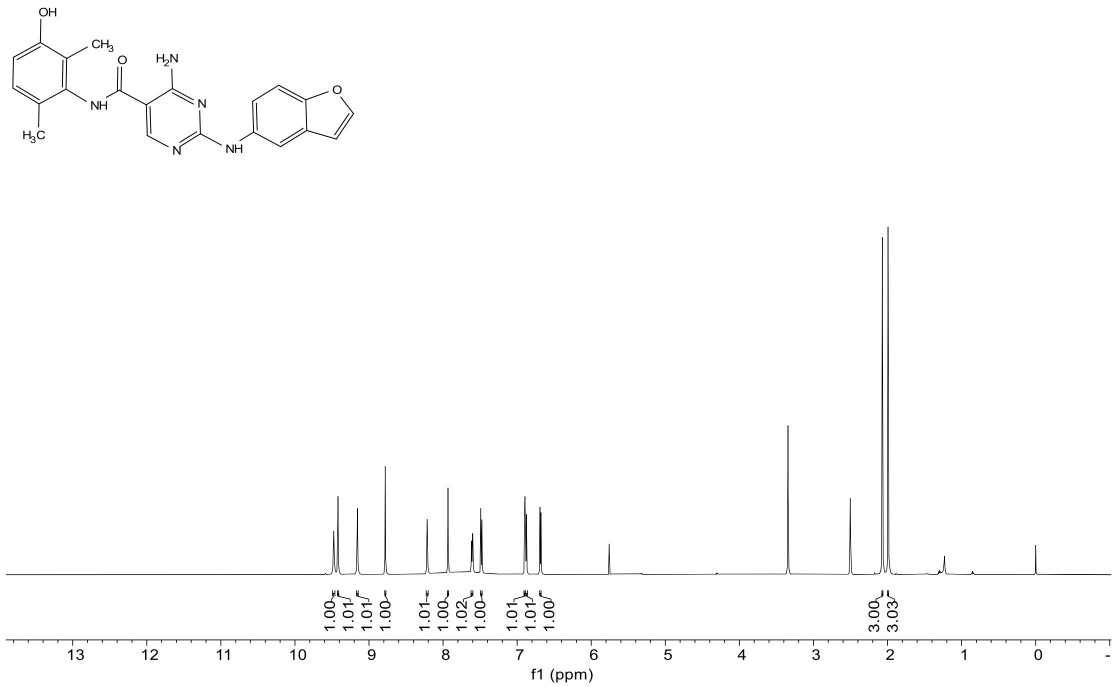

OH
CH3
O
H2N
N
NH
O
H3C
N
NH
1.00
1.01
1.01
1.00
1.01
1.00
1.02
1.00
1.01
1.01
1.00
3.00
3.03
13 12 11 10 9 8 7 6 5 4 3 2 1 0
f1 (ppm)

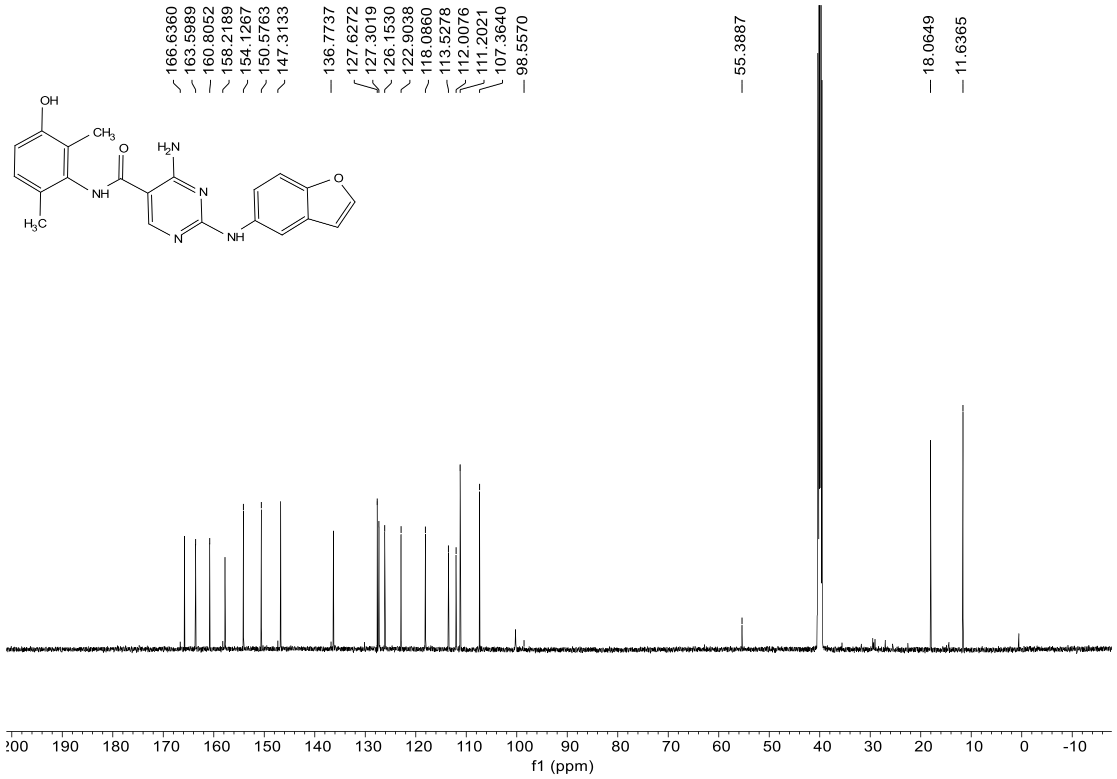

166.6360
163.5989
160.8052
158.2189
154.1267
150.5763
147.3133
136.7737
127.6272
127.3019
126.1530
122.9038
118.0860
113.5278
112.0076
111.2021
107.3640
98.5570
55.3887
18.0649
11.6365
OH
CH3
O
H2N
N
NH
O
H3C
N
NH
200 190 180 170 160 150 140 130 120 110 100 90 80 70 60 50 40 30 20 10 0 -10
f1 (ppm)

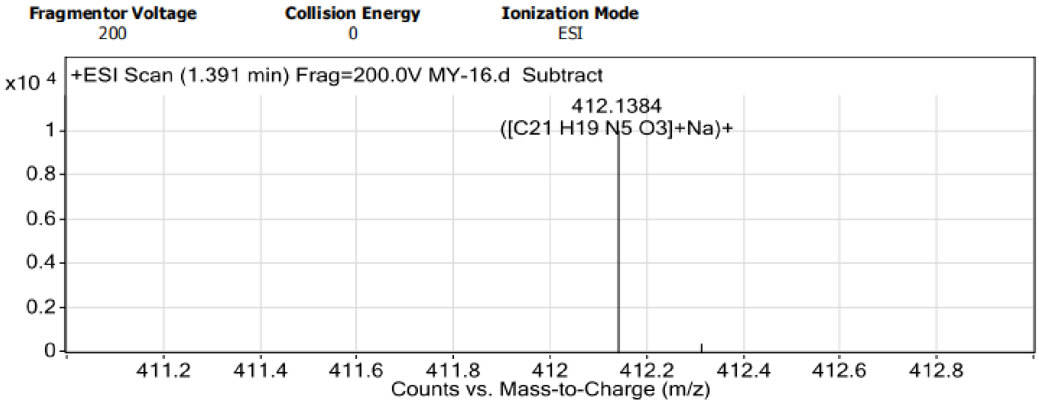

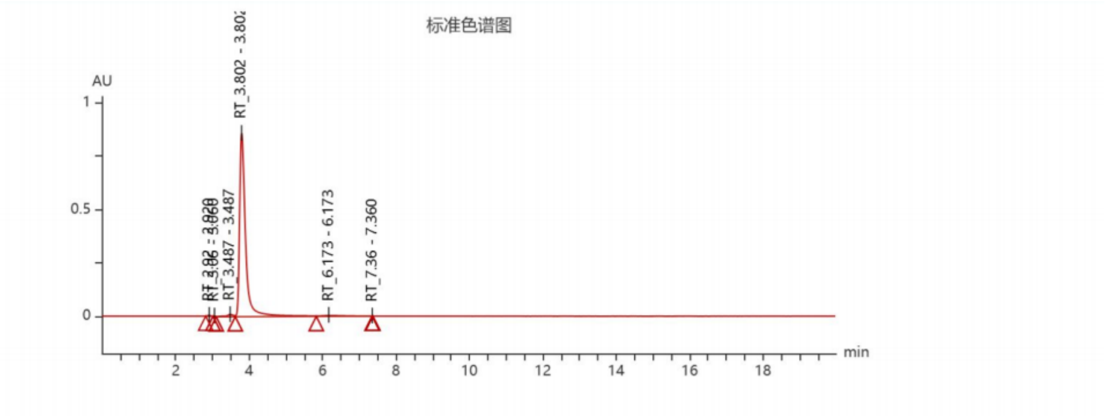

结果表

#	保留时间(min)	峰面积(μV*s)	峰高(μV)	百分面积(%)	百分高度(%)
1	2.920	22309.533	3785.375	0.221	0.430
2	3.060	5389.633	2335.923	0.053	0.265
3	3.487	148780.789	10780.697	1.473	1.224
4	3.802	9750627.183	860154.720	96.522	97.649
5	6.173	174739.327	3700.738	1.730	0.420
6	7.360	140.984	109.516	0.001	0.012

Figure S28 The ^{1}HNMR, ^{13}CNMR, HRMS and HPLC spectrum of Compound MY-21.
